# Population level consequences of facultatively cooperative behaviour in a stochastic environment

**DOI:** 10.1111/1365-2656.13618

**Published:** 2021-11-14

**Authors:** Michela Busana, Dylan Z. Childs, Terrence A. Burke, Jan Komdeur, David S. Richardson, Hannah L. Dugdale

**Affiliations:** ^1^ Groningen Institute for Evolutionary Life Sciences University of Groningen Groningen The Netherlands; ^2^ Department of Animal and Plant Sciences University of Sheffield Sheffield UK; ^3^ School of Biological Sciences University of East‐Anglia Norfolk UK; ^4^ Nature Seychelles Mahè Republic of Seychelles; ^5^ School of Biology Faculty of Biological Sciences University of Leeds Leeds UK

**Keywords:** Bayesian analyses, cooperative breeding, direct fitness benefits, environmental stochasticity, group living, life‐history tactics, matrix population model, population predictions

## Abstract

1. The social environment in which individuals live affects their fitness and in turn population dynamics as a whole. Birds with facultative cooperative breeding can live in social groups with dominants, subordinate helpers that assist with the breeding of others, and subordinate non‐helpers. Helping behaviour benefits dominants through increased reproductive rates and reduced extrinsic mortality, such that cooperative breeding might have evolved in response to unpredictable, harsh conditions affecting reproduction and/or survival of the dominants. Additionally, there may be different costs and benefits to both helpers and non‐helpers, depending on the time‐scale. For example, early‐life costs might be compensated by later‐life benefits. These differential effects are rarely analysed in the same study.
2. We examined whether helping behaviour affects population persistence in a stochastic environment and whether there are direct fitness consequences of different life‐history tactics adopted by helpers and non‐helpers.
3. We parameterised a matrix population model describing the population dynamics of female Seychelles warblers *Acrocephalus sechellensis,* birds that display facultative cooperative breeding. The stochastic density‐dependent model is defined by a (st)age structure that includes life‐history differences between helpers and non‐helpers and thus can estimate the demographic mechanisms of direct benefits of helping behaviour.
4. We found that population dynamics are strongly influenced by stochastic variation in the reproductive rates of the dominants, that helping behaviour promotes population persistence and that there are only early‐life differences in the direct fitness of helpers and non‐helpers.
5. Through a matrix population model, we captured multiple demographic rates simultaneously and analysed their relative importance in determining population dynamics of these cooperative breeders. Disentangling early‐life versus lifetime effects of individual tactics sheds new light on the costs and benefits of helping behaviour. For example, the finding that helpers and non‐helpers have similar lifetime reproductive outputs and that differences in reproductive values between the two life‐history tactics arise only in early life suggests that overall, helpers and non‐helpers have a similar balance of costs and benefits when analysing direct benefits. We recommend analysing the consequence of different life‐history tactics, during both early life and over the lifetime, as analyses of these different time frames may produce conflicting results.

## Graphical abstract

Cooperative breeding is widespread among passerine birds. Yet, the impact of environmental stochasticity on their population dynamics is poorly known. The authors looked at the Seychelles warblers (*Acrocephalus sechellensis*) as a case study. Helping behaviour had a positive effect on population dynamics because it could buffer fluctuations in reproductive success. Image credit: L. Lopera Doblas.

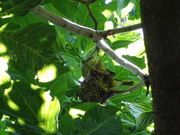

## 1 INTRODUCTION

Population dynamics of cooperatively breeding species are the consequence of individual demographic rates, the social structure in which individuals live and environmental drivers (e.g. Letcher et al., 1998; Ozgul et al., 2014). Because of the existence of costs and benefits of group living and cooperation, demographic rates are both negatively and positively influenced by the social environment (e.g. Berger et al., 2015; Ozgul et al., 2014). For example, birds with facultative cooperative breeding can live in groups characterised by a robust social structure: the dominants exhibit pair‐bonding behaviours, and dominance towards a variable number of subordinates. Some subordinates, called helpers, provide care to the young produced by the dominants. Less commonly, other subordinates, called non‐helpers, do not provide any help to the young (non‐helpers are observed in some species, such as white‐browed scrubwrens, Magrath & Whittingham, 1997, long‐tailed tits, Meade & Hatchwell, 2010; Tibetan ground tits, Cornwallis, 2018; and Seychelles warblers, Komdeur et al., 2016).

The presence of helpers can increase the fitness of the dominants by reducing the costs of parental care (Crick, 1992; Hammers, Kingma, van Boheemen, et al., 2021) or by increasing the survival of the offspring (Komdeur et al., 2016, but see, e.g. Griffin & West, 2002; Magrath & Yezerinac, 1997 for studies showing no effect of helping behaviour on reproductive success of the dominants). On the other hand, dominants influence the fitness of the helpers and non‐helpers by monopolising reproduction within the group (Hodge et al., 2008). Here, we define fitness from a population biology perspective and estimate fitness components from the survival of an individual and its expected production of offspring throughout its lifetime (Metcalf & Pavard, 2007). Therefore, fitness is determined by the combined patterns of survival and reproduction at each stage and age, st(age), of an individual. Helpers typically do not reproduce or have very low reproductive success (co‐breeding, Kaiser et al., 2019; Li & Brown, 2002; Nelson‐Flower et al., 2018), while subordinate non‐helpers do not reproduce (Clayton & Emery, 2007). Group relationships are complex and understanding the contribution of sociality to individual demographic rates is challenging but required if we are to fully comprehend the factors that impact the population dynamics of social species (Bateman et al., 2013; Ozgul et al., 2014).

The social environment is not the only factor driving population dynamics of cooperative species. Vital rates (e.g. survival and reproduction) are also influenced by resource availability, climate and/or density (Aars & Ims, 2002). Natural populations live in environments that are not constant but vary over time and space. Temporal variation in the environment might increase or decrease the amount of resources available. This variation translates into fluctuations in demographic rates and population dynamics (Frederiksen et al., 2014; Ohlberger et al., 2014). When food and space become limiting, individuals will compete to access these resources, resulting in density‐dependent demographic rates (Caswell, 2008). Density dependence and environmental variation might act simultaneously, and have a stronger impact on populations of limited size and localised distribution, such as populations living on islands (Lande et al., 2003).

In social species, total group size might positively impact survival and reproduction (Allee effects, Allee & Bowen, 1932; Angulo et al., 2018; Lerch & Abbott, 2020). For example, the presence of helpers is expected to buffer vital rates against fluctuations in population size due to environmental variation (Cornwallis et al., 2017; Walters et al., 2002). Indeed, cooperative breeding in passerines is driven by variable climatic conditions, such as precipitation levels (Guindre‐Parker & Rubenstein, 2020; Jetz & Rubenstein, 2011; but see Bourne et al., 2020 for the southern pied babbler where individual survival was reduced by adverse climatic conditions independently of group size and Gonzalez et al., 2013 for non‐passerines species where cooperative breeding can be favoured by environmental stability). Cooperation can be advantageous in temporally varying and unpredictable environments, because it allows sustained breeding during benign years, and reproduction in harsh years with low food availability (Rubenstein & Lovette, 2007). Additionally, density dependence could also favour the persistence of cooperation with cooperative species often persisting at carrying capacity (e.g. Brouwer et al., 2009) when space shortage forces some individuals to remain as subordinates (Koenig et al., 2011). Although environmental and social factors promote cooperative breeding (e.g. Jetz & Rubenstein, 2011; Rubenstein & Lovette, 2007), the demographic consequences of environmental variability and density dependence require further investigation. In fact, few data‐driven population models explicitly consider the simultaneous effects of environmental and social factors on individual fitness and population dynamics of cooperative breeders (e.g. Ozgul et al., 2014; Paniw et al., 2019). Few of these data‐driven population models have focused on birds (e.g. the pied babbler, Ridley et al., 2021; Wiley, 2017).

Birds with facultative cooperative breeding can reproduce as a pair or groups comprising a variable number of helpers and/or non‐helpers. In long‐lived species, both helpers and non‐helpers might wait for a few years before acquiring a dominant position (Downing et al., 2015). Their number of offspring produced often increases nonlinearly with age (Hammers et al., 2012). Being a helper or non‐helper may represent two alternative life‐history tactics that yield both costs and benefits. Non‐helpers do not incur the cost of raising the dominants' offspring and can devote more time to foraging or prospecting, to find a new territory. On the other hand, helping is costly but provides some indirect benefits (see Koenig & Dickinson, 2004). For example, helpers might gain valuable breeding experience (Komdeur, 1996), and increase their fitness through kin selection (Griffin & West, 2002). Helpers can also gain direct benefits when they reproduce (co‐breeding, Kingma, 2017; Richardson et al., 2002), although their reproductive success can be low (Hodge et al., 2008). However, comparative studies focus on the advantages of helping in the early life (i.e. first few years of life, Jennions & Macdonald, 1994; van de Crommenacker et al., 2011) and the lifetime fitness advantages for each individual tactic are unclear (Jennions & Macdonald, 1994). Given that cooperative breeders are long lived (Downing et al., 2015), it is important to understand if helping versus non‐helping tactics yield different lifetime fitness advantages or costs. Here, we tackled this question by focusing on direct fitness effects only.

Because the implications of facultative cooperative breeding on population dynamics are still poorly understood, we analyse how the presence of helpers and non‐helpers interacts with (st)age (stages [dominant, helper and non‐helper] and ages), environmental stochasticity and population density to influence population dynamics. We used the long‐term data from the Seychelles warblers *Acrocephalus sechellensis*, a small passerine with facultative cooperative breeding. In this species helper females are sometimes unrelated to the dominant breeders (Groenewoud et al., 2018) and there is a high rate of extra‐pair paternity (42% of the offspring are produced extra‐pair, Raj Pant et al., 2019). Helping females gain direct fitness advantages because they can lay an egg in the nest (about 47% of the helper females are co‐breeding and direct fitness benefits are about six times larger than indirect fitness benefits, Richardson et al., 2002). First, we test which demographic processes influence the population persistence of Seychelles warblers under stochastic environmental fluctuations. Second, we quantify the lifetime direct fitness differences between helpers and non‐helpers and their relative contribution to population dynamics. To this end we analysed the life cycle of Seychelles warblers and the probabilities that an individual will survive, reproduce and transition to dominant status in relation to age, stage, local social environment, environmental stochasticity and population density. These results were used to construct and parameterise a stochastic matrix population model (SMPM). With an adequate model, we predicted population dynamics under environmental fluctuations and investigated the fate of helpers and non‐helpers separately throughout their lifetime. Finally, applying a retrospective perturbation analyses, we identified how stochastic processes regulated population dynamics.

## 2 MATERIALS AND METHODS

### 2.1 Study species

Seychelles warblers are long‐lived passerines endemic to the Seychelles. Individuals are characterised by high adult survival (0.84 ± 0.04, Brouwer et al., 2006) and longevity is a primary determinant of reproductive output (Hammers et al., 2012). Individuals live in groups of two to five individuals (Brouwer et al., 2012), who defend the territory (Komdeur, 1992). Group living is mainly driven by a shortage of breeding vacancies (Komdeur, 1992; Komdeur et al., 1995) and ~50% of groups contain subordinates (Hammers, Kingma, Spurgin, et al., 2019). The status of each bird in a territory was determined by behavioural observations of at least an hour (van Boheemen et al., 2019): the dominants display pair‐bonding behaviours, helpers provide help at the nest by incubating and/or feeding, and non‐helpers are resident in a territory but do not incubate or feed the young (Richardson et al., 2002). Generally, when a subordinate was observed helping in a given season, it was also helping in the previous or following season(s). However, 2.9% of subordinates switched from being a helper to a non‐helper (or vice versa) during their lifetime (Supporting Information Appendix, Table A1). The group provides extended parental care to the offspring for 3–6 months during the major and minor reproductive seasons that correspond to the two moonson seasons (Brouwer et al., 2006; Komdeur, 1996). The presence of helpers increases the probability that the group produces offspring (Komdeur, 1996).

**FIGURE 1:**
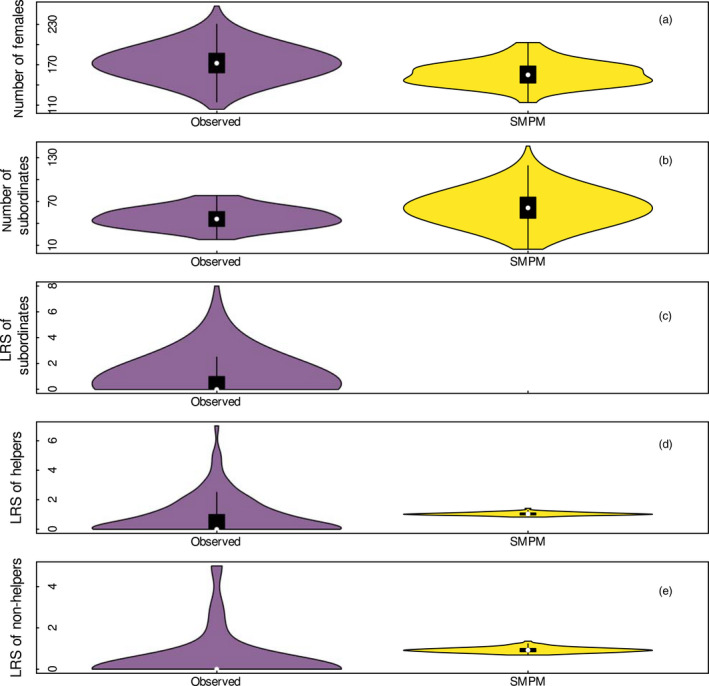
Comparison between observed population parameters (purple) for female Seychelles warblers on Cousin island, with those predicted by stochastic matrix population model (SMPM, yellow). Panels (a) and (b) show the total number of females and the number of subordinate females, respectively. The lifetime reproductive success (LRS) of subordinates is the LRS of all subordinates, including helpers, non‐helpers and subordinates without behavioural data to classify them as either helpers or non‐helpers (Panel c). The observed numbers of females and subordinates were calculated over 40 field seasons; while the sample sizes available to calculate the observed LRS were based on 570 subordinates, 114 helpers and 28 non‐helpers. Observations are noisy and driven by demographic and environmental stochasticity, while the SPMP includes only environmental stochasticity. This difference gives rise to some discrepancies between observations and predictions, most noticeably within the LRS analysis (Panel d for the LRS of helpers and Panel e for the LRS of non‐helpers). Data are plotted as a boxplot (with median and interquartile range) combined with a kernel density plot (R package vioplot 0.5)

We analysed data collected from 1994 to 2019 on Cousin island during the two reproductive seasons (Komdeur & Daan, 2005) on the life histories of marked females. Ten seasons were missing because field work was not conducted or was conducted for <30 days (see Supporting Information Appendix A3). Population parameters are reported in Figure 1 and Figure A1. Individuals were captured using mist nets, or as chicks in the nest and ringed at first capture with a metal ring (British Trust of Ornithology) and a unique combination of colour rings. About 97% of the population was individually recognisable since 1997 (Richardson et al., 2001). At capture, a small blood sample was collected to assign sex and parentage (Richardson et al., 2001; Sparks et al., 2020). Over 20 years of study we observed 958 females. A subset of 570 females was followed from birth to death. We inferred reproductive outputs from the pedigree (parentage assignment was completed using 30 microsatellite loci and three sexing markers with MasterBayes 2.52, Sparks et al., 2020). We define that an individual successfully reproduced when it produced an offspring that survived to at least 6 months of age. Females produced zero to eight female offspring in their lifetime, but the majority of females never successfully reproduced in their life (66.5%). Therefore, the distribution of lifetime reproductive success (LRS) in the population was not normally distributed, and both the median and mode were zero (Figure 1c–e).

To classify subordinates as helpers or non‐helpers, we performed nest observations of at least 1 hr during the incubation or feeding period. When a breeding attempt occurred when there was no fieldwork or when a reproductive attempt failed at early stages (Komdeur et al., 2016), we had no information to classify subordinates as helpers or non‐helpers. The number of individuals observed in each status, and how many of these individuals reached a dominant position, are reported in Table A1. Additional information about data collection can be found in Appendix A3.

All birds were captured and sampled under permission from the Seychelles Department of Environment and the Seychelles Bureau of Standards. All procedures were approved by the Ethical Review Committee at the University of East Anglia.

### 2.2 Data analyses

Statistical analyses were carried out in a Bayesian framework (Hamiltonian Monte Carlo sampler, Stan 2.19.0, Stan Development Team, 2020). We used *rethinking* (2.13, McElreath, 2019) and *cmdstanr* (0.1.3, Stan Development Team, 2020) as an interface to run Bayesian models from R 4.4.0 (R Core Team, 2020). We analysed 6‐month survival, reproduction and stage transition probabilities. Survival and reproduction probabilities of dominants and helpers/non‐helpers were estimated in two separate models. Statistical models were built using multilevel models (also known as mixed‐effects or hierarchical models with random effects or varying intercepts and slopes) with a binomial error distribution and a logit link function. The multilevel models estimated simultaneously both an intercept for each season and the variation among seasons. However, the probability of a dominant receiving help was modelled without varying intercepts because this strongly depends on the number of subordinates in the population in a given breeding season.

For each demographic probability, we analysed competing models containing a mixture of continuous and categorical explanatory variables: age (*a*), age squared (*a* ^2^), age cubic (*a* ^3^), status of a subordinate (either helper or non‐helper, *s*), standardised population size (*N*), helper presence (*q*), ratio between number of helpers and non‐helpers over the number of dominants (*r*), and if the mother was a dominant or a subordinate helper (*s_m_*). Detailed information of the variables considered for each vital rate is provided in Table A2. Not all variables were included in all models (Tables A2 and A3). The following variables included missing data: standardised population size, the ratio between the number of helpers and non‐helpers over the number of dominants, and the subordinate's status. Details on their imputation are reported in the Supporting Information (Supporting Information Appendix A6, Table A4, Figure A3). When analysing the reproduction probability of dominants, we tested if the effect of helper presence varied between seasons by allowing multilevel slopes for helper presence. Each competing model was ranked from lowest to highest using the Widely Applicable Information Criterion (WAIC, Watanabe, 2010) and the Akaike weight of each model (Gelman et al., 2014; McElreath, 2020). We also compared the 95% highest posterior density interval (HPDI) around parameter estimates among the set of competing models (McElreath, 2020). The most supported model was chosen to be the model with an Akaike weight of 0.8 or higher. When models had similar weights we considered the model with fewer parameters as the minimal adequate model (Burnham & Anderson, 2003).

All parameters were modelled using weakly regularising normal priors, except for variance parameters which were given exponential priors (see the R code on Gitlab for prior specifications and Supporting Information Appendix A7). For each model, we first simulated four separate chains of 30,000 iterations and then four separate chains of 60,000 iterations. The first 10,000 iterations were discarded to eliminate random variations associated with the initial conditions. To assess the autocorrelation and validity of the chains, we checked the effective number of samples with a confidence level and tolerance of both 0.05 using *mcmcse* (1.4‐1, Flegal et al., 2020) and ensured the Gelman Rubin convergence diagnostic $\hat{R}$ was <1.01. All chains were plotted and visually inspected to check for chain convergence (McElreath, 2020).

The transition probability from a helper or non‐helper to a dominant position was simulated in the demographic model as a weighted lottery where the probability depended on the number of territories with a vacant dominant position due to dominants' mortality, and helpers and non‐helpers had different probabilities to transition (Equation 9). Therefore, the transition probability is highly frequency dependent. The parameter values were estimated from a subset of the data (we only included subordinates that were classified as either helpers or non helpers, *n* = 648 data points). We omitted age from the analysis, because there is no evidence that age influenced this transition probability in females (Eikenaar et al., 2009). To estimate the difference between the probability (*β*) of helpers and non‐helpers transitioning to dominance from the data, we applied a generalised linear model with a binomial error distribution and a log link. The transitions of a helper and non‐helper acquiring a dominant position at time *t* followed the Bernoulli distribution with probabilities $p_{h}$ and $p_{u}$ respectively and were specified as such:

(1) $$\log\left(p_{h}\right)=\text{min}\left[1,\;\beta +\log\left(x\right)‐\log\left(e^{\beta }\;n_{h}+n_{u}\right)\right],$$

and

(2) $$\log\left(p_{u}\right)=\text{min}\left[1,\;\log\left(x\right)‐\log\left(e^{\beta }\;n_{h}+n_{u}\right)\right],$$

where $\text{min}\left[a,\;b\right]$ indicate the minimum of the numbers *a* and *b*, *x* is the number of vacant dominant positions in the population at time *t*, and $n_{h}$ and $n_{u}$ are the total number of surviving helpers and non‐helpers in the population respectively. We fitted the model using maximum likelihood and optimised it with the *optim* function from stats (R Core Team, 2020).

### 2.3 Population model

**FIGURE 2:**
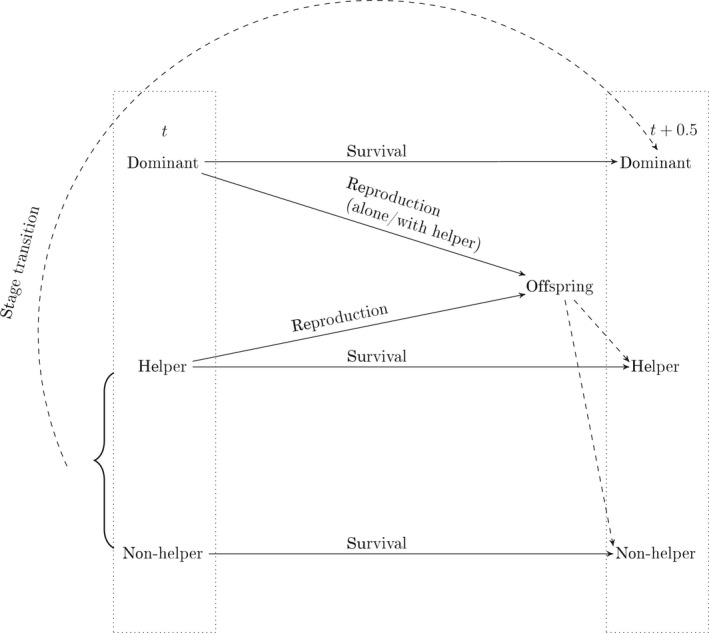
Life cycle of a female Seychelles warbler indicating census points, demographic processes (solid lines) and transitions between stages (dashed lines). At each time step, the population is censused before the next occurrence of reproduction. Individuals survive from time *t* to time t+0.5. Surviving helpers and non‐helpers can transition to a dominant position, represented by the curved dashed line. Helpers cannot transition to being a non‐helper or vice versa. Dominants reproduce either alone or with helpers. Reproduction is a two‐step process: the probability of a dominant or helper producing an offspring and the probability of the offspring entering the population as a helper or non‐helper at time t+0.5. Non‐helpers do not reproduce until they acquire a dominant position

The SMPM only tracks females, omits rare events such as broods of two offspring per mother (0.01% of the total number of reproductive events), subordinates switching individual tactics from helper to non‐helper during their lifetime (2.9% of subordinates), dominant females losing dominancy to become subordinates (5% of dominants), and assumes that the presence of helpers had a positive impact on the reproductive success of the dominant (Hammers et al., 2015; Komdeur et al., 2016). A graphical representation of the life cycle can be found in Figure 2.

The model consists of functions describing temporal dynamics of the age structure of dominants (*d*), helpers (*h*) and non‐helpers (*u*). The explanatory variables included are age (*a*), stage class (*s*), population size ($N_{t}=\sum_{a=0.5}^{m}d\left(a,\;t\right)+h\left(a,\;t\right)+u\left(a,\;t\right)$), ratio between the total number of helpers and non‐helpers over the total number of dominants in the population $\left(r_{t}=\frac{\sum_{a=0.5}^{m}\;h\left(a,\;t\right)+u\left(a,\;t\right)}{\sum_{a=0.5}^{m}\;d\left(a,\;t\right)}\right)$, the number of territories with a vacant dominant position (*x*) and helper presence in a territory (*q*). The associated functions are: (1) the 6‐month survival probability, $S\left(s,\;a,\;N,\;t\right)$ is the probability of an individual in stage *s* and age *a* surviving from time *t* to t+0.5; (2) the probability of a dominant receiving help, $p\left(a,\;r\right)$; (3) the probability of a dominant producing a female offspring (fledgling born at time *t* that survives to t+0.5), $R\left(d,\;a,\;N,\;q,\;t\right)$; (4) the probability of a helper producing a female offspring, $R\left(h,\;a,\;N,\;t\right)$; (5) the probability of a helper or non‐helper acquiring a dominant position between time *t* and t+0.5, $g_{h}\left(u,\;x,\;t\right)$, and $g_{u}\left(h,x,t\right)$ conditional on survival; and (6) the probability of an offspring entering the population as a helper, $f\left(a,\;N,\;t\right)$ or non‐helper, $\left(1‐f\left(a,\;N,\;t\right)\right)$. The terms $n\left(a,\;t\right)$, etc., denote the number of females, dominants, helpers and non‐helpers of age *a* at times *t* and t+0.5. The dynamics from time *t* to time t+0.5 are described by the following equations:

(3) $$\begin{array}{cc} d\left(a+0.5,\;t+0.5\right) & =g_{h}\left(u,\;x,\;t\right)S\left(h,\;a,\;N,\;t\right)h\left(a,\;t\right)\\ & \;+g_{u}\left(h,\;x,\;t\right)S\left(u,\;a,\;N,\;t\right)u\left(a,\;t\right)\\ & \;+S\left(d,\;a,\;N,\;t\right)d\left(a,\;t\right);\;a\geq 0.5, \end{array}$$

(4) $$\begin{array}{cc} h\left(a=0.5,\;t+0.5\right) & =\sum_{a=0.5}^{m}\;f\left(a,\;N,\;t\right)\;R\left(h,\;a,\;N,\;t\right)h\left(a,\;t\right)\\ & \;+\sum_{a=1}^{m}\;f\left(a,\;N,\;t\right)\;\left[p\left(a,\;r\right)\;R\left(d,\;a,\;N,\;q=1,\;t\right)\right.\\ & \;\left.+\left(1‐p\left(a,\;r\right)\right)\;R\left(d,\;a,\;N,\;q=0,\;t\right)\right]\;d\left(a,\;t\right), \end{array}$$

(5) $$h\left(a+0.5,\;t+0.5\right)=\left[1‐g_{h}\left(u,\;x,\;t\right)\right]\;S\left(h,\;a,\;N,\;t\right)\;h\left(a,\;t\right);\;a\geq 0.5,$$

(6) $$\begin{array}{cc} u\left(a=0.5,\;t+0.5\right) & =\sum_{a=0.5}^{m}\left[1‐f\left(a,\;N,\;t\right)\right]\;R\left(h,\;a,\;N,\;t\right)\;h\left(a,\;t\right)\\ & \;+\sum_{a=1}^{m}\left[1‐f\left(a,N,t\right)\right]\left[p\left(a,\;r\right)\;R\left(d,\;a,\;N,\;q=1,\;t\right)\right.\\ & \;\left.+\left(1‐p\left(a,\;r\right)\right)\;R\left(d,\;a,\;N,\;q=0,\;t\right)\right]\;d\left(a,\;t\right), \end{array}$$

(7) $$u\left(a+0.5,\;t+0.5\right)=\left[1‐g_{u}\left(h,\;x,\;t\right)\right]\;S\left(u,\;a,\;N,\;t\right)\;u\left(a,\;t\right);\;a\geq 0.5,$$

where *q* is a binary variable that equals one if the dominant receives help raising the offspring and zero otherwise; and *m* is the maximum age (15 years). Females can live up to 18 years of age (Hammers et al., 2015), but only 21 females reached the age of 16, so they were pooled together in a recursive maximum age class of 15 years. Only dominants and helpers can reproduce and the number of offsprings to be added to the population at time t+0.5 as helpers or non‐helpers is described in Equations (4) and (6) respectively. Equations (5) and (7) describe how the survival $S\left(s,\;a,\;N,\;t\right)$ and the transition to a dominant position $g_{h}\left(u,\;x,\;t\right)$ and $g_{u}\left(h,\;x,\;t\right)$ functions lower the number of helpers and non‐helpers from $h\left(a,\;t\right)$ and $u\left(a,\;t\right)$. Equation 3 describes how the survival function $S\left(d,\;a,\;N,\;t\right)$ lowers the number of dominants $d\left(a,\;t\right)$, while the transition probabilities from a helper/non‐helper stage $g_{h}\left(u,\;x,\;t\right)$ and $g_{u}\left(h,\;x,\;t\right)$ increase the number of dominants $d\left(a,\;t\right)$.

The probabilities to survive $S\left(s,\;a,\;N,\;t\right)$, reproduce $R\left(d,\;a,\;N,\;q,\;t\right)$, $R\left(h,\;a,\;N,\;t\right)$, be recruited into the population as a helper $f\left(a,\;N,\;t\right)$ vary with time *t* and are modelled by logistic functions of the form:

(8) $$pr_{t}=\frac{\exp\left(\beta_{0t}+\beta_{1}\;x_{1}+\cdots +\beta_{n}\;x_{n}\right)}{1+\exp\left(\beta_{0t}+\beta_{1}\;x_{1}+\cdots +\beta_{n}\;x_{n}\right)},$$

where *β*_1_, …, *β_n_* are slopes for different explanatory variables, *x*_1_, …, *x_n_*, such as age and population size; $\beta_{0t}$ are intercepts for each time step t. We assumed no temporal autocorrelation between time steps. To account for possible temporal covariation between vital rates we used a kernel resampling technique: we constructed separate kernels including all the parameter estimates from each season and sampled from these when simulating the data and building the SMPM (Ellner et al., 2016; Metcalf et al., 2015). The probability of receiving help $p\left(a,\;r\right)$ is also a binomial process but does not vary with time. The formula can be obtained from Equation 8 by substituting the intercepts $\beta_{0t}$ with a single intercept $\beta_{0}$.

Helpers and non‐helpers compete to acquire a dominant position. Subordinates mainly succeed in claiming a vacant territory after the dominant(s) dies (Eikenaar et al., 2009). Territory acquisition is a complex process and there are strong sex differences: older males were more likely to acquire a dominant position but age does not affect a female's probability of acquiring a territory (Eikenaar et al., 2009). Therefore, the probability of a helper $g_{h}\left(u,\;x,\;t\right)$ or non‐helper $g_{u}\left(h,\;x,\;t\right)$ acquiring a dominant position is modelled as a weighted lottery, where individuals can acquire a dominant position based on their stage and the presence of other individuals in the population. Let ${\text{ter}}_{\text{max}}$ be the maximum number of territories in the population, *x* the number of territories that are available for a helper or non‐helper to occupy at time *t* $\left(x={\text{ter}}_{\text{max}}‐\sum_{a=1}^{m}\left[S\left(d,\;a,\;N,\;t\right)\;d\left(a,\;t\right)\right]\right)$ and β the difference in the probability of helpers versus non‐helpers, then the probability of a helper acquiring a dominant position $g_{h}\left(u,\;x,\;t\right)$ is:

(9) $$g_{h}\left(u,\;x,\;t\right)=\text{min}\left[1,\;\frac{e^{\beta }\;x}{\sum \left[e^{\beta }\;S\left(h,\;a,\;N,\;t\right)\;h\left(a,\;t\right)\;+S\left(u,\;a,\;N,\;t\right)\;u\left(a,\;t\right)\right]}\right],$$

and the probability of a non‐helper acquiring a dominant position $g_{u}\left(h,\;x,\;t\right)$ is:

(10) $$g_{u}\left(h,\;x,\;t\right)=\text{min}\left[1,\frac{x}{\sum \left[e^{\beta }\;S\left(h,\;a,\;N,\;t\right)\;h\left(a,\;t\right)\;+S\left(u,\;a,\;N,\;t\right)\;u\left(a,\;t\right)\right]}\right],$$

Under reasonable parameter values the following identity is true:

(11) $$\sum \left[g_{h}\left(u,\;x,\;t\right)\;S\left(h,\;a,\;N,\;t\right)\;h\left(a,\;t\right)+g_{u}\left(h,\;x,\;t\right)\;S\left(u,\;a,\;N,\;t\right)\;u\left(a,\;t\right)\right]=x.$$

For convenience we can write Equations (3), (4), (5), (6), (7) in matrix notation (matrices are denoted with bold‐face uppercase letters, vectors with bold lowercase letters) as follow:

(12) $$n\left(t+0.5\right)=K\left(t\right)\;n\left(t\right),$$

where K is the nonlinear projection matrix and $n\left(t\right)$ is the stage distribution at time *t* (see Supporting Information Appendix A1). Age is a discrete variable ranging from 0.5 to a maximum age *m* of 15 years. Offspring enter the population at age 0.5 as either helper or non‐helper. The half‐year age increments are consistent with the 6‐month time steps implemented in the model. We numerically calculated the (st)age distributions of each cohort of individuals and derived the mean and normalised age distribution for each stage. The population was modelled with a maximum number of dominant individuals (${\text{ter}}_{\text{max}}=111.2$ individuals), which corresponds to the mean number of dominant positions observed. From the simulated population vectors, we also calculated the predicted mean number of dominants, helpers and non‐helpers, and their age distribution and the ratio between the number of helpers and non‐helpers over the number of dominants. In a (st)age structured population model the reproductive value is an estimate of the relative contribution of each stage and age to the future population size (Caswell, 1982). We calculated age‐specific reproductive value for helpers $v_{h}\left(t\right)$, and non‐helpers $v_{u}\left(t\right)$ to test for age‐specific differences between the two stages.

The model was constructed and analysed in R (R Core Team, 2020). We ran the stochastic simulation for 10,000 time steps with a burnin of 7,000 simulations. Matrix population models can be used to calculate life‐history descriptors such as LRS, a generation measure of fitness (Caswell, 2001). The performance of a cohort is tracked in terms of survivorship and reproduction. The survivorship function is derived from the survival function $S\left(s,a,N,t\right)$ and gives the fraction of individuals of a cohort alive through time. The total number of offspring produced by a cohort is the sum of all offspring produced through time by the cohort (Caswell, 2001). To better describe the fate of helpers and non‐helpers, we define two separate survivorship functions for each stage. From these functions we derived the mean LRS of helpers LRS_h_ and non‐helper LRS_u_. Because our model is stochastic and density dependent, we followed the fate of a hundred cohorts of helpers and non‐helpers through time and numerically calculated their survivorship and reproduction (see Supporting Information Appendices A1 and S2 for mathematical equations). By estimating the median (and interquartile range) LRS for cohorts of helpers and non‐helpers in a SMPM, we accounted for the fact that a cohort experienced stochastic variations in their vital rates through time steps. For example, a cohort could experience low reproductive rates in one season but higher rates in the next season. As such, the LRS of helpers and non‐helpers are normally distributed around their means and do not contain zeros. The variation around the means is due to environmental stochasticity. These calculations do not account for stochastic demographic processes that could affect a single individual (e.g. an individual could be predated and die before reproducing). Demographic stochasticity is common in observed populations, where LRS is often skewed towards zero (e.g. Tuljapurkar et al., 2020). To overcome this limitation, we also calculated the moments of LRS using Markov chains with rewards (Caswell, 2011; van Daalen & Caswell, 2017). To numerically solve the SMPM, we included the parameter estimates obtained from statistical analyses of the observed dataset. To assess the fit of the SMPM, we compared the observed data to the model predictions. A good fit corresponded to predictions that were close approximations of the data.

### 2.4 Perturbation analyses

To investigate how variations in the vital rates impacted the dynamics of the population models, we applied different forms of perturbation analyses. Our SMPM is defined by a mixture of time‐varying and fixed parameters. Concerning the effect of time‐varying parameters, we applied a life table response experiment (LTRE) with a random design (Caswell, 2010). LTRE aims to quantify the contribution of a vital rate *x* with mean $\mu_{x}$ and standard deviation $\sigma_{x}$ to population parameters *y* under different conditions (Caswell, 2001). We defined conditions as small perturbations ($\Delta =\varepsilon^{1/3}$, where ε corresponds to the machine precision) of the intercepts of survival, reproduction and the probability of an offspring becoming a helper. Assuming no temporal autocorrelation, the sensitivity of *y* to changes in *x* is given by $dy=\frac{\partial y}{\partial \mu_{x}}\;d\mu_{x}+\frac{\partial y}{\partial \sigma_{x}}\;d\sigma_{x}$ (Vindenes, 2010). The variance of a demographic statistic *y*, $Var\left(\hat{y}\right)$ can be approximated with a nonparametric approach (Ellner et al., 2016) through a machine‐learning algorithm (randomForest 4.6‐12, Liaw & Wiener, 2002). The random decision forest algorithm creates an extensive collection of uncorrelated decision trees and ranks the relative importance of the time‐varying parameters on changes in $\hat{y}$. We applied LTRE to the mean population size, $\hat{N}$ because the SMPM is both stochastic and density dependent. Further details to calculate LTRE can be found in the Supporting Information Appendix A10.

In addition to LTRE we calculated elasticities of the mean $\hat{N}$ with respect to different parameters (Table 1) using the following expression (Grant & Benton, 2000):

(13) $$\frac{\partial \log\hat{N}}{\partial \log x};$$

where *x* represents a parameter in the model. LTRE and elasticity analysis answer complementary questions: LTRE tackles how stochastic variation in vital rates is driving variations in population size, while the elasticity analysis predicts how population dynamics respond to proportional changes in vital rates (Caswell, 2001). To understand how the population would respond to changes in the demographic functions and to the parameters governing cooperative breeding, we perturbed the intercepts of the demographic functions and additionally the parameter for helper presence in the reproduction of dominants.

### 2.5 Posterior predictive checks

In the observed dataset, 565 subordinate individuals had no behavioural information to classify them as helper or non‐helper, and for six seasons we had no information on the total population size or the ratio between the number of helpers and non‐helpers over the number of dominants. We imputed missing data in the statistical analysis in a Bayesian framework (see the data analyses section). However, imputing missing data introduces uncertainty in the parameter estimates used to parameterise the SMPM. To validate the imputation and perform posterior predictive checks, we used an individual‐based model (IBM) and simulated 1,000 artificial datasets (Gelman et al., 2013). An IBM limits issues with the effects of unknown variables and helps obtain artificial data that can be compared to the wild population (Ellner et al., 2016; Rees et al., 2014). The IBM was constructed based on survival, reproduction and stage transition described in the data analysis sub‐section. In the IBM, we classified all subordinates as either helper or non‐helper. The total number of subordinates was derived from the number of all observed subordinates irrespective of type, while the proportion of helpers and non‐helpers within the population was estimated based on the observed number of helpers and non‐helpers. Therefore, the IBM was comparable to the wild population.

To numerically solve the IBM, we used the posterior predictive distribution of the statistical analyses. The posterior distribution accounts for uncertainty in the parameter estimates. If the statistical analyses are a good fit, we should generate data via the IBM that resembles the observations (Gabry et al., 2019; Gelman et al., 2013). To make sure we obtained predictions resembling the observed metrics from the real population, we inspected the artificial data (after randomly excluding 10% of the data) with density plots and metric summaries of the population parameters.

## 3 RESULTS

### 3.1 Data analyses

The number of dominants was stable on Cousin island, while the number of subordinates fluctuated between 18 and 80 individuals (Figure A1; Figure 1). The fact that the population was relatively stable suggests that there is high density dependence but the lack of fluctuations reduces our statistical power to detect it (Figure A1). The error estimate around the parameter estimates for the standardised population size was large and overlapped zero. Despite this limitation, we retained the models including population size to account for density dependence in reproduction and survival of helpers and non‐helpers.

**FIGURE 3:**
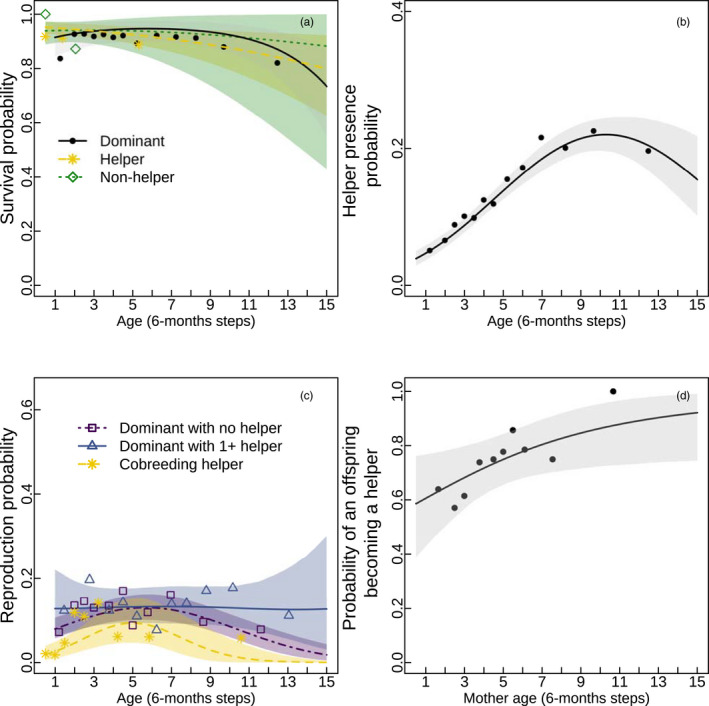
Posterior predictions for the observed demographic rates in the life cycle of Seychelles warblers. The graphs describe: (a) the survival probability of dominants (black), helpers (yellow) and non‐helpers (green), (b) the probability that a dominant receives help, and (c) the probability of successfully producing an offspring as a function of age for dominants (purple), dominants with helper(s) (blue) and helpers (yellow); and (d) the probability that a offspring is a helper as a function of the age of its mother. The variables included in the model but omitted in the graph (e.g. population size) are kept constant and equal to their mean. The bordering shaded areas are the 95% percentile intervals of the expected mean intercepts. These areas are very wide because they were calculated by marginalising over the varying intercepts, except for graph (b), which did not include varying intercepts. The dots, crosses, squares and triangles are fractions of the data defined as a function of mean age for a series of age classes calculated from the percentiles of the age distribution

Survival probability declined with age and increasing population size for helpers and non‐helpers, but was relatively stable for dominants until a decline after 9 years of age (Figure 3a; Figures A4 and A5). The probability of a dominant receiving help had a quadratic relationship with age, peaking at 10 years of age (Figure 3b), and it increased as the ratio of the total number of subordinates over the number of dominants present in the population increased (Figure A12). In general, reproduction probability declined as the population size increased (density dependence, Figures A8 and A10). Reproduction probability of dominants without helpers and reproduction of helpers increased with age followed by a decline later in life, whereas it was constant for dominants with one or more helpers (Figure 3c; Figures A7 and A9). Dominants had higher success rates than helpers (higher intercepts, Figure 3c). When a dominant reproduced with one or more helpers, its probability of success was independent of age (Figure 3c; Figure A7), and this was independent of seasonal variation. The probability of an offspring being a helper increased as its mother's age increased (Figure 3d; Figure A11). Parameter estimates for all models including their 95% highest posterior density interval can be found in Tables A3 and A4. Density plots of the parameter estimates are also shown in the Figures A13–A21.

Helpers were less likely to acquire a dominant position than non‐helpers (*β* = −0.549; 95% confidence intervals [−0.262, −0.836]). Because there were more helpers than non‐helpers in the population, the absolute number of helpers recruiting into the dominant class was higher than non‐helpers.

### 3.2 Population model

The predicted number of females and of subordinates are close approximations of the observations (the median, upper and lower quartiles, Figure 1a,b). However, the distribution shape of the data differs between predictions and observations (Figure 1a,b): predictions are normally distributed, while the corresponding observations are skewed towards the median. Observations are noisy and driven by both demographic and environmental stochasticity. The SMPM includes only environmental stochasticity assuming that all individuals within a cohort experience identical vital rates throughout their lifetime. This assumption gives rise to small discrepancies. Figure A1 compares the observed and predicted number of dominants versus helpers and non‐helpers. Because many behavioural observations were missing, the plot also shows the observed total number of subordinates (helpers, non‐helpers, and subordinates with unknown stage).

**FIGURE 4:**
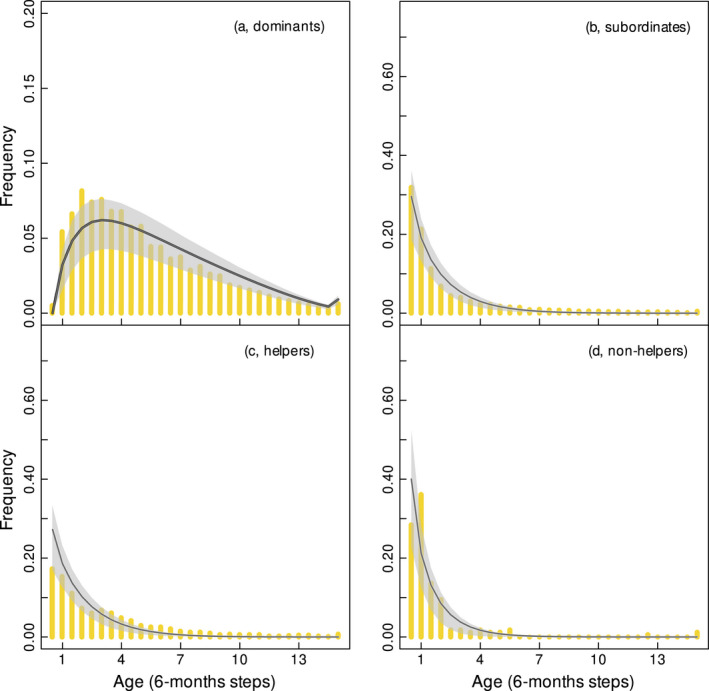
Mean age distributions (lines) and interquantile range (grey shaded polygons) for dominants (a), subordinates (b), helpers (c) and non‐helpers (d) derived from the stochastic matrix population model (SMPM). Yellow vertical bars indicate the observed distribution of age over the entire study. Subordinates (b) include different groups in the SMPM versus the observed data. In the SMPM they represent helpers and non‐helpers, while in the observations they include helpers, non‐helpers and those subordinates that could not be classified as either helpers or non‐helpers because of a lack of behavioural observations. Therefore, the observed age distribution of the helpers and the non‐helpers combined differs from the age distribution of all the observed subordinates (Figure A2). Sample size differs in the four graphs (*n* = 5,200 for dominant, *n* = 2,341 for subordinates, *n* = 822 for helpers, *n* = 169 for non‐helpers). The range of the *y‐*axis differs in plot (a) (0.00–0.20) versus the other plots (0.00–0.70)

**FIGURE 5:**
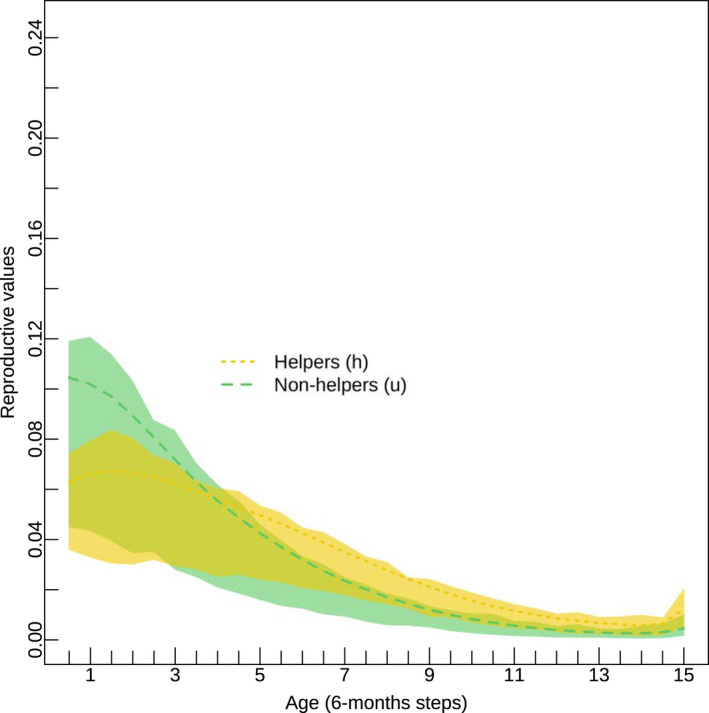
Age‐specific reproductive values distributions calculated from the stochastic matrix population model (SMPM). Reproductive values measure the relative contribution of each (st)age to future generations. Reproductive values of helpers (yellow dashed line) slowly decline through time, while the reproductive values of non‐helpers (green longdashed line) show a steep decline with age. Shaded polygons represent the interquartile ranges around the mean values

The predicted age distribution of dominants (Figure 4a), and of subordinates (Figure 4b) captured the observed distribution of ages. The age distribution of helpers (Figure 4c) and non‐helpers (Figure 4d) was plotted separately and shows that ~74% of the non‐helpers were predicted to be <2 years old, versus ~59% of the helpers. These plots show some discrepancies with the observed data. Discrepancies likely arise from the differences in sample size between the graphs. In fact, Figure 4b includes a larger dataset with additional 1,350 data points than Figure 4c,d (due to missing behavioural data), so there is more power to estimate age distributions accurately. For helpers and non‐helpers, reproductive values declined with age, but this decline assumed different shapes (Figure 5; Figure A25). At young ages (<4 years) non‐helpers yielded a higher reproductive value, but this declined rapidly; after 4 years of age, the reproductive value of helpers was higher. Because the survival probabilities of helpers and non‐helpers were identical, differences in the reproductive values arose from differences in reproduction and transition probability to a dominant class.

The predicted LRS of helpers and of non‐helpers were overlapping (median and interquartile range are $1.04\left[0.94,1.14\right]$ and $0.92\left[0.82,1.05\right]$ respectively, Figure 1d,e; Figure A26) indicating that helpers and non‐helpers have, on average, similar reproductive outputs over their lifetime. However, the predicted values and the kernel density plots differ with the observed values. The observed LRS values are not normally distributed, with significant variations between individuals, because the majority of females (66.5%) never successfully reproduced in their life. On the other hand, the SMPM did not account for variations in individual quality or stochastic demographic processes at the individual level. The predicted LRS is estimated by following the fate of cohorts of helpers and non‐helpers throughout their lifetime. As such, the predicted LRS values are normally distributed, and their range of variation is less pronounced than in the observed LRS. The moments of LRS account for individual stochasticity and show that the remaining LRS of helpers and non‐helpers were similar in their mean, variance, coefficient of variation and skewness (Figure A27).

### 3.3 Perturbation analyses

**FIGURE 6:**
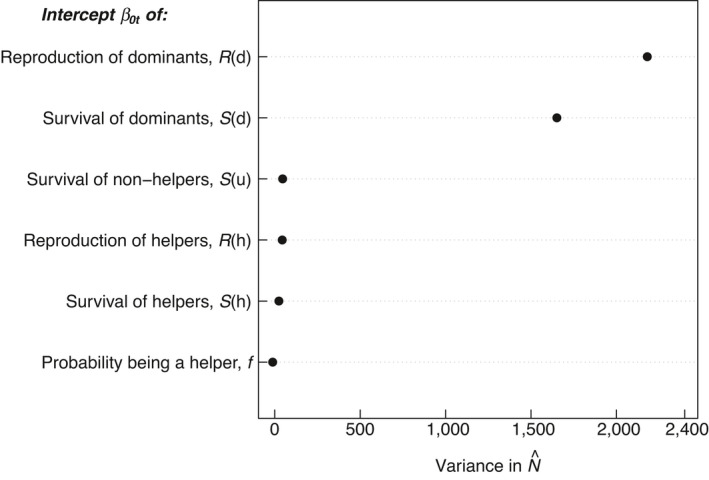
Results of life table response experiment (LTRE) analysis showing the relative importance of time‐varying parameters to changes in the mean population size, $\hat{N}$. The analysis suggests that variation in the intercepts of reproduction of dominants (*R*(*d*)) was the most important, followed by variation in the intercepts for the survival of dominants (S(*d*)). Variation in the reproduction probability of helpers (*R*(*h*)), in the survival of helpers (*S*(*h*)) and non‐helpers (*S*(*u*)) and of the probability of an offspring becoming a helper (probability being a helper, *f*) did not cause substantial variation in population size

The LTRE analysis showed how the variance in $\hat{N}$ is partitioned between variations in the underlying stochastic parameters: the intercepts of survival, reproduction and the probability of an offspring entering into the population as a helper. The LTRE accounted for 99% of the variation in $\hat{N}$. Based on the random forest algorithm, the most important term influencing population stability was variation in the intercepts of reproduction probability of the dominants (57%), followed by survival of dominants (40%; Figure 6). Variation in the intercepts of survival of helpers and non‐helpers, reproduction of helpers and of the probability of offspring becoming a helper did not explain much of the variation observed in population size (1%, 1%, 1% and ~0% respectively; Figure 6). Therefore, population fluctuations were driven by demographic effects, and in particular by variation in the reproductive output of dominants.

The elasticity analysis shows how the mean $\hat{N}$ could increase if selected parameter values in the population were to change. Proportional increments in the probability that a dominant received help had the largest positive effect on $\hat{N}$, followed by the survival probability of helpers and non‐helpers, the survival probability of dominants and the advantage conferred to a dominant when reproducing with a helper present (Table 1). The other parameters yielded smaller effects. According to the elasticity analysis, potential changes in the social and demographic effects could have a substantial impact on determining the mean $\hat{N}$.

**TABLE 1:** Results of the elasticity analysis to specific parameters of the stochastic matrix population model in the following functions: survival probabilities of dominants $S\left(d,\;a,\;N,\;t\right)$, survival probabilities of helpers and non‐helpers $S\left(h/u,\;a,\;N,\;t\right)$, probability of reproduction for dominants $R\left(d,\;a,\;N,\;q,\;t\right)$, probability of reproduction for helpers $R\left(h,\;a,\;N,\;t\right)$, probability of offspring being helpers $f\left(a,\;N,\;t\right)$ and probability of dominants to receive help $p\left(a,\;r\right)$

Function	Parameter	Type	$\frac{\partial \log\hat{N}}{\partial \log x}$
$S\left(d,\;a,\;N,\;t\right)$	Intercept	Multilevel	0.043
$S\left(h,\;a,\;N,\;t\right)$	Intercept	Multilevel	0.022
$S\left(u,\;a,\;N,\;t\right)$	Intercept	Multilevel	0.005
$R\left(d,\;a,\;N,\;q,\;t\right)$	Intercept	Multilevel	0.012
$R\left(d,\;a,\;N,\;q,\;t\right)$	Contrast helper presence, *q*	One level	0.043
$R\left(h,\;a,\;N,\;t\right)$	Intercept	Multilevel	0.001
$f\left(a,\;N,\;t\right)$	Intercept	Multilevel	0.001
$p\left(a,\;r\right)$	Intercept	One level	0.071

### 3.4 Posterior predictive checks

The simulation of artificial data through the IBM starting from the posterior distribution of the statistical analyses generated populations that resembled the observations (Supporting Information Appendix A11, Figures A22–A24). This result suggests that the statistical analyses are a good fit despite the missing data.

## 4 DISCUSSION

By integrating (st)age‐specific demographic rates, life‐history tactics, direct fitness advantages of cooperation, density dependence and environmental stochasticity in the SMPM, we modelled the population dynamics of Seychelles warblers and showed how extrinsic drivers interact with individual life‐history tactics to drive the population dynamics of this species. Our findings support the idea that cooperative behaviour in passerines contributes positively to the reproductive success of the dominants, despite stochastic variations in environmental conditions (Jetz & Rubenstein, 2011). Although there is substantial variation in survival and reproduction probabilities by age, the presence of both helpers and non‐helpers in the population is likely maintained because both life‐history strategies have similar lifetime direct advantages in terms of survival and reproduction probabilities. Helpers increase their direct fitness by attempting to reproduce while waiting to obtain a dominant position elsewhere. On the other hand, non‐helpers maximise their reproduction by finding a dominant position.

### 4.1 Population dynamics

The perturbation analyses of the SMPM showed that Seychelles warbler dynamics were governed mainly by the reproduction of dominants in the population. The stochastic population size $\hat{N}$ is an index of the effects of environmental stochasticity on population persistence. The LTRE results demonstrate that most of the variation found in $\hat{N}$ were explained by contributions from the reproduction probability of dominants. Additionally, the results of the elasticity analysis suggest that increasing the probability that a dominant is helped yields a substantial increase in the mean of $\hat{N}$ (Table 1). When we varied the likelihood of dominants receiving help, the total population size increased because more offspring were produced seasonally. Previous empirical analyses confirm that the presence of helpers improves the reproductive success of the dominants (Richardson et al., 2002, but a negative effect can occur in territories of low and medium quality when there are two or more helpers, Komdeur, 1994). Throughout the study period, the number of offspring that were produced varied, but the number of dominants observed was stable. We modelled the population to reflect the observed conditions on Cousin island and limited the population size by setting a maximum number of territories. Seychelles warblers live in an enclosed island, and a shortage of territories regulates population dynamics (Komdeur, 1992). However, climate change will impact the conditions on the island and increased sea level might reduce the space available for species to breed (Han et al., 2010). Future work could investigate the minimum territory number required to support a viable population by simulating scenarios with a decreasing number of dominant positions.

### 4.2 Life‐history tactics

Thanks to the SMPM, we could describe the direct fitness differences between helpers and non‐helpers. The rapid decline in the reproductive value of non‐helpers after 4 years of age suggests that non‐helpers could acquire a dominant position earlier in their life to maximise their contribution to the next generation. In terms of recruiting to the dominant class, non‐helpers had a relative advantage over helpers. Therefore it was more likely that non‐helpers rather than helpers transitioned to a dominant position and potentially started to reproduce. Helpers took, on average, a longer time to acquire a dominant position, but while helpers, they reproduced at low rates compared to dominants. In contrast, non‐helpers only reproduce when they became dominants. Despite the possibility of co‐breeding, helpers did not have a lifetime reproduction advantage over non‐helpers suggesting that the population has reached an evolutionarily stable state. The similarity in LRS of helpers and non‐helpers might seem puzzling at first because we would expect that, by helping at the nest, helpers could gain greater direct fitness benefits than the costs incurred and so have an advantage over non‐helpers (McGowan et al., 2003; van de Crommenacker et al., 2011). Our findings contradict this expectation, but they refer to direct fitness benefits only. Indirect fitness effects through kin selection might also contribute to selection on helping behaviour (West et al., 2007).

Indirect fitness benefits can be gained by subordinates that provide care to relatives who share genes inherited from a common ancestor or with young that carry a gene for cooperation. The role of kinship in the evolution of cooperation has been shown in social insects (e.g. Nonacs, 2011), but it is more controversial within bird species where helping is directed to non‐relatives in 45% of the species (Riehl, 2013). In the Seychelles warblers, the level of relatedness between helpers and nondescendent offspring is low (0.13 ± 0.23 or 0.08 ± 0.25 for female and male subordinates respectively) due to a high level of extra‐pair paternity (Richardson et al., 2002). Because helpers can reproduce by co‐breeding, their direct benefits are, on average, six times more than their indirect benefits (Richardson et al., 2002).

Following the recommendations of Richardson et al. (2002), we only included direct fitness advantages in the SMPM, and we showed that the LRS of helpers and non‐helpers are similar. However, it is worth noting that there were more helpers (*n* = 49.3 ± 16.5 individuals) than non‐helpers (*n* = 11.9 ± 5.7 individuals) in the SMPM. Why would that be if helping is costly, and there are no lifetime direct fitness advantages of helping behaviour, and indirect fitness benefits are relatively small? Each life‐history tactic might be advantageous, depending on the local circumstances. For example, individuals differ in body condition, and only individuals in good condition help (van de Crommenacker et al., 2011), which may explain why we observed 2.9% individuals switching between being a helper and a non‐helper in different seasons. On the other hand, group‐level pressures (pay to stay hypothesis, Gaston, 1978) or habitat quality (Covas t al., 2004; Komdeur, 1992) might also influence individuals in their decisions to help. In long‐lived species displaying complex social systems, it is crucial to analyse the costs and benefits of decisions throughout the lives of the individuals and to also account for the effect of the local environment on these decisions. Our work investigated the vital rates of individuals through their lives, but future work should test how including multiple sources of individual variation and local drivers impacts our predictions on population dynamics.

### 4.3 Combining data with the SMPM

Building on previous work showing the ecological significance of age, life‐history tactics, and density dependence on Seychelles warblers (Brouwer et al., 2009; Hammers et al., 2012), we analysed seasonal variations in the main demographic parameters driving population dynamics of the system. As Ellner et al. (2016) have indicated, these analyses combined with the SMPM provide a better understanding of the system by capturing all the key demographic rates simultaneously. The population level predictions of our SMPM overlapped with the observed data. This suggests that the models capture population dynamics of the birds. Using IBM exclusively would have allowed inclusion of variations in individual quality and spatial structure (Letcher et al., 1998). Similarly, the implementation of integral projection models would have allowed modelling individual variation (Ellner et al., 2016). We appreciate that by projecting the population over the mean values with a population‐level model, we might have lost information about individual differences (DeAngelis et al., 1992). By excluding spatial structure, we failed to incorporate the complex spatial interactions between neighbouring territories that regulated competition, dispersal and territory acquisition. Territory acquisition was modelled as a simplified weighted lottery where all helpers and non‐helpers competed simultaneously for the vacant positions. This might be not realistic, since the majority of subordinates disperse and settle in a territory close to their natal one (Eikenaar et al., 2009).

Finally, we focused exclusively on females, because helpers are mainly females (Richardson et al., 2002). However, there are strong sex‐specific differences in life‐history tactics among Seychelles warblers. For example, males and females have different dispersal tactics (Komdeur, 1992; Kingma et al., 2016) and age‐specific differences in territory acquisition (Eikenaar et al., 2009). Our model could be improved by including both sexes, spatial structure and individual variations. We expect that this amelioration would improve the quantitative results of our model. Our aim here was not to replicate all the demographic processes but rather to find the minimal adequate demographic functions that describe population dynamics. Our methodology could also be applied to other cooperative breeding species, which represent 8.9% of the non‐marine birds (Jetz & Rubenstein, 2011) to test how demographic processes interact with life‐history tactics in different systems.

### 4.4 Conclusions and recommendations for future research

Our results provide evidence that it is possible to describe the population dynamics of cooperative breeding by simply integrating the demographic and social effects regulating the probabilities of transition between st(ages) in a variable environment. Moreover, we also clarified how differences between individual life‐history tactics are maintained within the Seychelles warbler population. Failing to account for the lifetime consequences of different life‐history tactics on the demographic rates might bias our understanding of how variation in life‐history traits is preserved in a population, and we recommend caution interpreting data where only early‐life effects are reported.

## CONFLICT OF INTEREST

We confirm that there is no actual or potential conflict of interest that could inappropriately influence our work.

## AUTHORS' CONTRIBUTIONS

M.B., D.Z.C., T.A.B. and H.L.D. conceived the study, designed hypotheses and methodology; M.B. analysed the data and wrote the first draft; T.A.B., J.K., D.S.R. and H.L.D. maintain the long‐term dataset. All authors contributed critically to the interpretation of the results, reviewed the manuscript and gave final approval for publication.

## Supporting information

Supplementary Material

## Data Availability

Data and data description are available from Dataverse https://doi.org/10.34894/UWUGZH UWUGZH_2021 (Busana et al., 2021). Code is available on GitLab https://gitlab.com/michebio/smpm_syechelles_warbler.
