# Supplementary material for: Population level consequences of facultatively cooperative behaviour in a stochastic environment
Source: J Anim Ecol. 2021 Nov 14;91(1):224–40. doi: 10.1111/1365-2656.13618 (PMC9299144; doi:10.1111/1365-2656.13618)
Supplement: Supplementary file 1 — Supplementary Material [file JANE-91-224-s001.pdf]

Appendix to Population level consequences of facultatively cooperative behavior in a stochastic environment

M Busana[*], DZ Childs, TA Burke, J Komdeur, DS Richardson, HL Dugdale

*Corresponding author: m.busana@rug.nl

Contents

Contents 1

List of Figures 2

List of Tables 3

A1 Equations and projection matrix 4

A2 Life history 10

A3 Data collection and data files 14

A4 Statuses of the observed females 17

A5 All Explanatory variables Considered 18

A6 Minimal statistical models 20

A7 Priors in Bayesian statistics 28

[*]m.busana@rug.nl

A8 Posterior predictive checks: predictive plots **28**

A9 Posterior predictive checks: density plots **57**

A10 Life table response experiment **80**

A11 Results individual based model **81**

A12 Additional results for fitness components **84**

Bibliography **87**

List of Figures

A1 Observed and predicted number of individuals 16

A2 Age distributions by status 18

A3 Missing data mechanism 25

A4 Survival probability of dominant females as a function of age 29

A5 Survival probability of helpers and non-helpers as a function of age 34

A6 Survival probability of helpers and non-helpers as a function of population size 40

A7 Reproduction probability of dominants as a function of age 41

A8 Reproduction probability of dominants as a function of population size 45

A9 Reproduction probability of helpers as a function of age 46

A10 Reproduction probability of helpers as a function of population size 51

A11 Probability of an offspring becoming a helper as a function of the age of its mother 52

A12 Probability that a helper is present in a territory 56

A13 Posterior distribution of the survival probability of dominants 57

A14 Posterior distribution of the survival probability of helpers and non-helpers . . 58

A15 Posterior distribution of the varying intercepts of the survival probability 59

A16 Posterior distribution of the reproduction probability of dominants 64

A17 Posterior distribution of the varying intercepts of the reproduction probability of dominants 65

A18 Posterior distribution of the reproduction probability of helpers 69
A19 Posterior distribution of the reproduction probability of helpers 70
A20 Posterior distribution of the probability of an offspring becoming a helper . . . 75
A21 Posterior distribution of the varying intercepts of the probability of an offspring becoming a helper 76
A22 Predicted number of individuals in the IBM 81
A23 Comparison between observations and the IBM population parameters 82
A24 Mean age-distributions in the IBM 83
A25 Difference in the reproductive values of helpers and non-helpers 84
A26 Comparison between predicted LRS of helpers and non-helpers 85
A27 Statistics of remaining LRS of helpers and non-helpers as a function of age . . . 86

List of Tables

A1 Statuses of the females 17
A2 Explanatory variables considered building the statistical models 19
A3 Minimal statistical models 21
A4 Parameter estimates for imputed data 26

A1 EQUATIONS AND PROJECTION MATRIX

The baseline equation describing the stochastic matrix population model (SMPM) is:

$$\boldsymbol{n}(t+0.5) = \boldsymbol{K}(t) \times \boldsymbol{n}(t) \tag{1}$$

where the age a terms from 0.5 to m, $\boldsymbol{K}$ is the projection matrix and $\boldsymbol{n}(t)$ is the right eigenvector associated with the leading eigenvalue of the matrix $\boldsymbol{K}(t)$. In other words $\boldsymbol{n}(t)$ is a vector of ages arranged within the three stages (in order dominant, helper and non-helper female). The above equation can be rewritten as such:

$$\begin{bmatrix} d(0.5, t+0.5) = 0 \\ d(1, t+0.5) \\ \vdots \\ d(m-0.5, t+0.5) \\ d(m, t+0.5) \\ \hline h(0.5, t+0.5) \\ h(1, t+0.5) \\ \vdots \\ h(m-0.5, t+0.5) \\ h(m, t+0.5) \\ \hline u(0.5, t+0.5) \\ u(1, t+0.5) \\ \vdots \\ u(m-0.5, t+0.5) \\ u(m, t+0.5))x_i \end{bmatrix} = \boldsymbol{K}(t) \times \begin{bmatrix} d(0.5, t) = 0 \\ d(1, t) \\ \vdots \\ d(m-0.5, t) \\ d(m, t) \\ \hline h(0.5, t) \\ h(1, t) \\ \vdots \\ h(m-0.5, t) \\ h(m, t) \\ \hline u(0.5, t) \\ u(1, t) \\ \vdots \\ u(m-0.5, t) \\ u(m, t))x_i \end{bmatrix} \tag{2}$$

The matrix $\boldsymbol{K}(t)$ can be written in extended form as such:

$$
\boldsymbol{K}(t)=\begin{bmatrix}
0 & 0 & \cdots & \cdots & 0 & 0 & 0 & 0 & \cdots & \cdots & 0 & 0 & 0 & 0 & \cdots & \cdots & 0 & 0 \\
0 & 0 & \cdots & \cdots & 0 & 0 & g_{\mathrm{h}}(h,x,t)S(h,0.5,N,t) & 0 & \cdots & \cdots & 0 & 0 & g_{\mathrm{u}}(u,x,t)S(u,0.5,N,t) & 0 & \cdots & \cdots & 0 & 0 \\
\vdots & S(d,1,N,t) & 0 & 0 & \cdots & 0 & 0 & g_{\mathrm{h}}(h,x,t)S(h,1,N,t) & 0 & \cdots & 0 & 0 & 0 & g_{\mathrm{u}}(u,x,t)S(u,1,N,t) & 0 & \cdots & 0 & 0 \\
\vdots & 0 & \ddots & 0 & \cdots & 0 & \vdots & 0 & \ddots & 0 & \cdots & \vdots & \vdots & 0 & \ddots & 0 & \cdots & \vdots \\
0 & \vdots & 0 & \ddots & 0 & 0 & 0 & \vdots & 0 & \ddots & 0 & 0 & 0 & \vdots & 0 & \ddots & 0 & 0 \\
0 & 0 & \cdots & 0 & S(d,m-0.5,N,t) & S(d,m,N,t) & 0 & 0 & 0 & 0 & g_{\mathrm{h}}(h,x,t)S(h,m-0.5,N,t) & g_{\mathrm{h}}(h,x,t)S(h,m,N,t) & 0 & 0 & 0 & 0 & g_{\mathrm{u}}(u,x,t)S(u,m-0.5,N,t) & g_{\mathrm{u}}(u,x,t)S(u,m,N,t) \\
0 & f(1,N,t)[p(1,r)R(d,1,N,1,t)+(1-p(1,r))R(d,1,N,0,t)] & \cdots & \cdots & f(m-0.5,N,t)[p(m-0.5)R(d,m-0.5,N,1,t)+(1-p(m-0.5))R(d,m-0.5,N,0,t)] & f(m,N,t)[p(m,r)R(d,m,N,1,t)+(1-p(m,r))R(d,m,N,0,t)] & f(0.5,N,t)R(h,0.5,N,t) & f(1,N,t)R(h,1,N,t) & \cdots & \cdots & f(m-0.5,N,t)R(h,m-0.5,N,t) & f(m,N,t)R(h,m,N,t) & 0 & 0 & \cdots & \cdots & 0 & 0 \\
0 & 0 & \cdots & \cdots & 0 & 0 & (1-g_{\mathrm{h}}(h,x,t))S(h,0.5,N,t) & 0 & \cdots & \cdots & 0 & 0 & 0 & 0 & \cdots & \cdots & 0 & 0 \\
\vdots & \cdots & \cdots & \cdots & \cdots & \vdots & \vdots & (1-g_{\mathrm{h}}(h,x,t))S(h,1,N,t) & 0 & \cdots & \cdots & \cdots & \cdots & \cdots & \cdots & \cdots & \cdots & \vdots \\
0 & 0 & \cdots & \cdots & 0 & 0 & 0 & 0 & \ddots & 0 & 0 & 0 & 0 & 0 & \cdots & \cdots & 0 & 0 \\
0 & 0 & \cdots & \cdots & 0 & 0 & 0 & 0 & 0 & \ddots & 0 & 0 & 0 & 0 & \cdots & \cdots & 0 & 0 \\
0 & 0 & \cdots & \cdots & 0 & 0 & 0 & 0 & \cdots & \cdots & (1-g_{\mathrm{h}}(h,x,t))S(h,m-0.5,N,t) & (1-g_{\mathrm{h}}(h,x,t))S(h,m,N,t) & 0 & 0 & \cdots & \cdots & 0 & 0 \\
0 & (1-f(1,N,t))[p(1,r)R(d,1,N,1,t)+(1-p(1,r))R(d,1,N,0,t)] & \cdots & \cdots & (1-f(m-0.5,N,t))[p(m-0.5,r)R(d,m-0.5,N,1,t)+(1-p(m-0.5,r))R(d,m-0.5,N,0,t)] & (1-f(m,N,t))[p(m,r)R(d,m,N,1,t)+(1-p(m,r))R(d,m,N,0,t)] & (1-f(0.5,N,t))R(h,0.5,N,t) & (1-f(1,N,t))R(h,1,N,t) & \cdots & \cdots & (1-f(m-0.5,N,t))R(h,m-0.5,N,t) & (1-f(m,N,t))R(h,m,N,t) & 0 & 0 & \cdots & \cdots & 0 & 0 \\
0 & 0 & \cdots & \cdots & 0 & 0 & 0 & 0 & \cdots & \cdots & 0 & 0 & (1-g_{\mathrm{u}}(u,x,t))S(u,0.5,N,t) & 0 & \cdots & \cdots & 0 & 0 \\
\vdots & \cdots & \cdots & \cdots & \cdots & \cdots & \cdots & \cdots & \cdots & \cdots & \cdots & 0 & 0 & (1-g_{\mathrm{u}}(u,x,t))S(u,1,N,t) & 0 & \cdots & \cdots & \vdots \\
0 & 0 & \cdots & \cdots & 0 & 0 & 0 & 0 & \cdots & \cdots & \cdots & \cdots & 0 & 0 & \ddots & 0 & 0 & 0 \\
0 & 0 & \cdots & \cdots & 0 & 0 & 0 & 0 & \cdots & \cdots & \cdots & \cdots & \cdots & \cdots & 0 & \ddots & 0 & 0 \\
0 & 0 & \cdots & \cdots & 0 & 0 & 0 & 0 & \cdots & \cdots & 0 & 0 & 0 & 0 & \cdots & \cdots & (1-g_{\mathrm{u}}(u,x,t))S(u,m-0.5,N,t) & (1-g_{\mathrm{u}}(u,x,t))S(u,m,N,t)
\end{bmatrix} \quad (3)
$$

Because this format cannot be easily read unless the reader zooms in the page, we also report below a magnified version of different sections of the projection matrix:

$$\boldsymbol{K}(t) = \begin{bmatrix} A1 & B1 & C1 \\ A2 & B2 & C2 \\ A3 & B3 & C3 \end{bmatrix} \tag{4}$$

where

$$\begin{bmatrix} A1 \\ A2 \\ A3 \end{bmatrix} = \begin{bmatrix}
0 & 0 & \cdots & \cdots & 0 & 0 \\
0 & 0 & \cdots & \cdots & 0 & 0 \\
\vdots & S(d,1,N,t) & 0 & 0 & \cdots & 0 \\
\vdots & 0 & \ddots & 0 & \cdots & 0 \\
0 & \vdots & 0 & \ddots & 0 & 0 \\
0 & 0 & \cdots & 0 & S(d,m-0.5,N,t) & S(d,m,N,t) \\
0 & f(1,N,t)[p(1,r)R(d,1,N,1,t)+(1-p(1,r))R(d,1,N,0,t)] & \cdots & \cdots & f(m-0.5,N,t)[p(m-.5)R(d,m-0.5,N,1,t)+(1-p(m-.5))R(d,m-0.5,N,0,t)] & f(m,N,t)[p(m,r)R(d,m,N,1,t)+(1-p(m,r))R(d,m,N,0,t)] \\
0 & 0 & \cdots & \cdots & 0 & 0 \\
\vdots & \cdots & \cdots & \cdots & \cdots & \vdots \\
0 & 0 & \cdots & \cdots & 0 & 0 \\
0 & 0 & \cdots & \cdots & 0 & 0 \\
0 & 0 & \cdots & \cdots & 0 & 0 \\
0 & (1-f(1,N,t))[p(1,r)R(d,1,N,1,t)+(1-p(1,r))R(d,1,N,0,t)] & \cdots & \cdots & (1-f(m-0.5,N,t))[p(m-.5,r)R(d,m-0.5,N,1,t)+(1-p(m-.5,r))R(d,m-0.5,N,0,t)] & (1-f(m,N,t))[p(m,r)R(d,m,N,1,t)+(1-p(m,r))R(d,m,N,0,t)] \\
0 & 0 & \cdots & \cdots & 0 & 0 \\
\vdots & \cdots & \cdots & \cdots & \cdots & \cdots \\
0 & 0 & \cdots & \cdots & 0 & 0 \\
0 & 0 & \cdots & \cdots & 0 & 0 \\
0 & 0 & \cdots & \cdots & 0 & 0
\end{bmatrix}; \quad (5)$$

and

$$
\begin{bmatrix} B1 \\ B2 \\ B3 \end{bmatrix} =
\begin{bmatrix}
0 & 0 & \cdots & \cdots & 0 & 0 & \\
g_{\mathrm{h}}(h,x,t)S(h,0.5,N,t) & 0 & \cdots & \cdots & 0 & 0 & \\
 & 0 & g_{\mathrm{h}}(h,x,t)S(h,1,N,t) & 0 & \cdots & 0 & 0 \\
\vdots & 0 & \ddots & 0 & \cdots & \vdots & \\
0 & \vdots & 0 & \ddots & 0 & 0 & \\
0 & 0 & 0 & 0 & g_{\mathrm{h}}(h,x,t)S(h,m-0.5,N,t) & g_{\mathrm{h}}(h,x,t)S(h,m,N,t) & \\
f(0.5,N,t)R(h,0.5,N,t) & f(1,N,t)R(h,1,N,t) & \cdots & \cdots & f(m-0.5,N,t)R(h,m-0.5,N,t) & f(m,N,t)R(h,m,N,t) & \\
0 & 0 & \cdots & \cdots & 0 & 0 & \\
\vdots & (1-g_{\mathrm{h}}(h,x,t))S(h,1,N,t) & 0 & \cdots & \cdots & \cdots & \\
0 & 0 & \ddots & 0 & 0 & 0 & \\
0 & 0 & 0 & \ddots & 0 & 0 & \\
0 & 0 & \cdots & \cdots & (1-g_{\mathrm{h}}(h,x,t))S(h,m-0.5,N,t) & (1-g_{\mathrm{h}}(h,x,t))S(h,m,N,t) & \\
(1-f(0.5,N,t))R(h,0.5,N,t) & (1-f(1,N,t))R(h,1,N,t) & \cdots & \cdots & (1-f(m-0.5,N,t))R(h,m-0.5,N,t) & (1-f(m,N,t))R(h,m,N,t) & \\
0 & 0 & \cdots & \cdots & 0 & 0 & \\
\cdots & \cdots & \cdots & \cdots & \cdots & 0 & \\
0 & 0 & \cdots & \cdots & \cdots & \cdots & \\
0 & 0 & \cdots & \cdots & \cdots & \cdots & \\
0 & 0 & \cdots & \cdots & 0 & 0 &
\end{bmatrix}; \tag{9}
$$

and

$$\begin{bmatrix} C1 \\ C2 \\ C3 \end{bmatrix} = \begin{bmatrix}
0 & 0 & \cdots & \cdots & 0 & 0 \\
g_{\mathrm{u}}(u,x,t)S(u,0.5,N,t) & 0 & \cdots & \cdots & 0 & 0 \\
0 & g_{\mathrm{u}}(u,x,t)S(u,1,N,t) & 0 & \cdots & 0 & 0 \\
\vdots & 0 & \ddots & 0 & \cdots & \vdots \\
0 & \vdots & 0 & \ddots & 0 & 0 \\
0 & 0 & 0 & 0 & g_{\mathrm{u}}(u,x,t)S(u,m-0.5,N,t) & g_{\mathrm{u}}(u,x,t)S(u,m,N,t) \\
0 & 0 & \cdots & \cdots & 0 & 0 \\
0 & 0 & \cdots & \cdots & 0 & 0 \\
\cdots & \cdots & \cdots & \cdots & \cdots & \vdots \\
0 & 0 & \cdots & \cdots & 0 & 0 \\
0 & 0 & \cdots & \cdots & 0 & 0 \\
0 & 0 & \cdots & \cdots & 0 & 0 \\
0 & 0 & \cdots & \cdots & 0 & 0 \\
(1-g_{\mathrm{u}}(u,x,t))S(u,0.5,N,t) & 0 & \cdots & \cdots & 0 & 0 \\
0 & (1-g_{\mathrm{u}}(u,x,t))S(u,1,N,t) & 0 & \cdots & \cdots & \vdots \\
0 & 0 & \ddots & 0 & 0 & 0 \\
\cdots & \cdots & 0 & \ddots & 0 & 0 \\
0 & 0 & \cdots & \cdots & (1-g_{\mathrm{u}}(u,x,t))S(u,m-0.5,N,t) & (1-g_{\mathrm{u}}(u,x,t))S(u,m,N,t)
\end{bmatrix} \tag{7}$$

A2 LIFE HISTORY

We report mathematical equations for the survivorship and fertility functions of a cohort of female helpers (h) and non-helpers (u). The intercepts of the vital rates vary stochastically between seasons. Therefore, cohorts experience different environments after birth, and there will be differences in the survivorship and fertility functions between cohorts. Below, we explain how we calculate lifetime reproductive success of helpers, LRS_{h}, and non-helpers, LRS_{u}.

The number of helpers in a cohort $n_{\mathrm{h}}(0.5, t = t_0)$ born at time t_0 is given by

$$n_{\mathrm{h}}(0.5, t = t_0) = f(a, N, t_0 - 0.5)\,[\sum_{a=1}^{m} R(d, a, N, t_0 - .5) + \sum_{a=.5}^{m} R(h, a, N, t_0 - .5)] \tag{8}$$

and the survivorship function of helpers at age .5 is given by

$$L_{\mathrm{h}}(.5, t_0) = I \tag{9}$$

where I is the identity matrix. In the following years, helpers can survive and transition to a dominant position or survive and stay in the same stage as helpers. We denote the survivorship to age a of helpers that do not transition to a dominant position with $L_{\mathrm{hh}}(a, t_0)$, and of helpers that transition to a dominant position with $L_{\mathrm{hd}}(a, t_0)$. For example, the survivorship to age 1 of helpers born at time t_0, that remain helpers is:

$$L_{\mathrm{hh}}(1, t_0 + .5) = (1 - g_{\mathrm{h}}(h, x, t_0))\, S(h, .5, N, t_0)\, L_{\mathrm{hh}}(.5, t_0) \tag{10}$$

while the survivorship of helpers that transition to a dominant position at age 1 is:

$$L_{\mathrm{hd}}(1, t_0 + .5) = g_{\mathrm{h}}(h, x, t_0)\, S(h, .5, N, t_0)\, L_{\mathrm{hh}}(.5, t_0) \tag{11}$$

Moreover, for any $a > 1$ and $a \le m$ the probability that helpers survive to age a and remain

helpers is:

$$L_{\mathrm{hh}}(a, t_0 + a) = (1 - g_{\mathrm{h}}(h, x, t_0 + a - .5))\, S(h, a - .5, N, t_0 + a - .5)\, L_{\mathrm{hh}}(a - .5, t_0 + a - .5) \tag{12}$$

and the probability that a dominant, that was previously a helper, survives to age a or that a helper transition to a dominant position at age a is:

$$\begin{aligned} L_{\mathrm{hd}}(a, t_0 + a) =& S(d, a - .5, N, t_0 + a - .5)\, L_{\mathrm{hd}}(a - .5, t_0 + a - .5) + \\ & g_{\mathrm{h}}(h, x, t_0 + a - .5)\, S(h, a - .5, N, t_0 + a - .5)\, L_{\mathrm{hh}}(a - .5, t_0 + a - .5) \end{aligned} \tag{13}$$

Therefore the survivorhip function for the helpers is:

$$L_{\mathrm{h}}(.5, t = t_0) = I \tag{14}$$

$$L_{\mathrm{h}}(1, t = t_0 + .5) = S(h, .5, N, t_0) \tag{15}$$

For $1.5 \leq a \geq m$ at intervals of .5:

$$\begin{aligned} L_{\mathrm{h}}(a, t_0 + a) =& (1 - g_{\mathrm{h}}(h, x, t_0 + a - .5))\, S(h, a - .5, N, t_0 + a - .5)\, L_{\mathrm{hh}}(a - .5, t_0 + a - .5) \\ & S(d, a - .5, N, t_0 + a - .5)\, L_{\mathrm{hd}}(a - .5, t_0 + a - .5) + \\ & g_{\mathrm{h}}(h, x, t_0 + a - .5)\, S(h, a - .5, N, t_0 + a - .5)\, L_{\mathrm{hh}}(a - .5, t_0 + a - .5) \end{aligned} \tag{16}$$

We can now write the equation for the helpers in a cohort as such:

$$L_{\mathrm{h}}(.5, t_0)[n_{\mathrm{h}}(.5, t_0)] = n_{\mathrm{h}}(.5, t_0) \tag{17}$$

and

$$L_{\mathrm{h}}(a, t_0 + a)[n_{\mathrm{h}}(.5, t_0)] =: n_{\mathrm{h}}(a, t_0 + a - .5) \tag{18}$$

The expected number density of offspring produced at age a by a helper born at time t_0,

$M(a, t_0 + a - .5)$ is given by:

$$M_\mathrm{h}(.5, t_0) = R(h, .5, N, t_0)\, L_\mathrm{hh}(.5, t_0) \tag{19}$$

$$M_\mathrm{h}(a, t_0{+}a) = R(h, a, N, t_0{+}a{-}.5)\, L_\mathrm{hh}(a, t_0{+}a{-}.5){+}R(d, a, N, t_0{+}a{-}.5)\, L_\mathrm{hd}(a, t_0{+}a{-}.5) \tag{20}$$

The lifetime reproduction LRS_h of a cohort of helpers born at time t_0 is given by:

$$LRS_\mathrm{h}(t_0) = \frac{\sum_{a=.5}^{A} M_\mathrm{h}(a, t_0 + a - .5)}{n_\mathrm{h}(0.5, t_0)} \tag{21}$$

where A is a large maximum age, which is larger than the maximum age m and t increases accordingly. In the SMPM, we allowed individuals of age m to continue to survive and reproduce at a low rate. To improve our estimate of LRS_h, we allowed a mother to reproduce until a maximum age of 100.

Similarly, we defined the survivorship function, and lifetime reproductive of non-helpers born in a cohort. The number of non-helpers (u) in a cohort born at time t_0, $n_\mathrm{u}(.5, t_0)$ is given by the identity:

$$n_\mathrm{u}(.5, t_0) = [1 - f(t_0 - .5)]\,[\sum_{a=1}^{m} R(d, a, N, t_0 - .5) + \sum_{a=.5}^{m} R(h, a, N, t_0 - .5)] \tag{22}$$

The survivorhip function for the non-helpers is:

$$L_\mathrm{u}(.5, t = t_0) = I \tag{23}$$

$$L_\mathrm{u}(1, t = t_0 + .5) = S(u, .5, N, t_0) \tag{24}$$

For $1.5 \leq a \geq m$ at intervals of .5:

$$\begin{aligned} L_\mathrm{u}(a, t_0 + a) =&(1 - g_\mathrm{u}(u, x, t_0 + a - .5))\, S(u, a - .5, N, t_0 + a - .5)\, L_\mathrm{uu}(a - .5, t_0 + a - .5) \\ & S(d, a - .5, N, t_0 + a - .5)\, L_\mathrm{ud}(a - .5, t_0 + a - .5) + \\ & g_\mathrm{u}(u, x, t_0 + a - .5)\, S(u, a - .5, N, t_0 + a - .5)\, L_\mathrm{uu}(a - .5, t_0 + a - .5) \end{aligned} \tag{25}$$

We can now write the equation for the non-helpers in a cohort as such:

$$L_{\mathrm{u}}(.5, t_0)[n_{\mathrm{u}}(.5, t_0)] = n_{\mathrm{u}}(.5, t_0) \tag{26}$$

and

$$L_{\mathrm{u}}(a, t_0 + a)[n_{\mathrm{u}}(.5, t_0)] =: n_{\mathrm{u}}(a, t_0 + a - .5) \tag{27}$$

Non-helpers cannot reproduce until they acquire a dominant position. The expected density of offspring produced by a cohort of non-helpers born at time t_0 is defined as follow:

$$M_{\mathrm{u}}(a, t_0 + a - .5) = R(d, a, N, t_0 + a - .5)\, L_{\mathrm{ud}}(a, t_0 + a - .5) \tag{28}$$

The lifetime reproduction LRS_{u} of non-helpers born at time t_0 is defined as:

$$LRS_{\mathrm{u}}(t_0) = \frac{\sum_{a=.5}^{A} M_{\mathrm{u}}(a, t_0 + a - .5)}{n_{\mathrm{u}}(0.5, t_0)} \tag{29}$$

where a, A and t_0 are defined as above.

A3 DATA COLLECTION AND DATA FILES

The study site, Cousin island is located in the Republic of Seychelles (29 km^2, 4°20′ S, 55°40′ E). Data on Seychelles warblers were extracted from the warbler database version 0.56.1. There are two main breeding seasons: the main breeding season between June-September during the southeast monsoon season when food availability is high, and the minor breeding season between January-March during the northwest monsoon season (Hammers *et al.*, 2012). Therefore, birds were monitored in each season each year from 1994 till 2015 (except between January-March 1995, 1996, 1997, 2001, 2002, 2003, 2006, 2007, 2010 and 2011 when fieldwork was not conducted and/or was conducted for a short period of time, i.e. < 1 month). The total number of data points is 7541.

The population is at carrying capacity of ~ 320 individuals (Komdeur & Pels, 2005) and migration/emigration to/from other islands is negligible (Komdeur *et al.*, 2016). Population size was reduced after 2004 and 2011. These fluctuations were due to the translocation of 24 and 23 females (58 and 59 individuals including males, respectively) to the islands of Denis and Fregate, respectively (Wright *et al.*, 2014). Translocations were carried out for conservation purposes to expand the range of the species.

Individuals were captured using mist nets and unringed individuals were ringed with a metal ring (British Trust of Ornithology) and colour-banded (Komdeur & Pels, 2005). A small blood sample of $\sim 25\,\mu l$ was collected to determine the sex of each individual and assign parentage (Hadfield, Richardson & Burke, 2006; Edwards, Burke & Dugdale, 2017). The age at 1st capture was estimated by a mixture of behavioural and morphological characteristics. The maximum age observed in the population was 18 years.

Each year after 1996 *ca* 97% of all mature individuals (> 6 months) were color-banded (Richardson *et al.*, 2001, between 1994 and 1996 the number of unringed individuals was not recorded in the warbler database (version 0.56.1) so we cannot provide a proportion). The re-sighting probability of dominant individuals is very high (0.98 $\pm$ 0.01 SE; Brouwer *et al.*, 2006). When an individuals was not observed for 1 year and during the subsequent years, we assumed that it had died in the first year it was missing (Hammers *et al.*, 2013).

We recorded the status of each individual in each season, whether it survived to the fol-

lowing season, whether it produced a female offspring that survived to at least 6 months of age (recruit), the status of the offspring, and whether each group included helpers and/or non-helpers. Over the 824 individuals that were observed at least once in their life as subordinates, 19 were classified as helpers or non-helpers in different seasons. In 35 cases dominant females produced two recruits per season (0.01% of the total number of reproductive events, $n = 3762$). It is also common to have a female helper per group (99.99% of cases). However, 0.01% of the dominant females had two or more female helpers. Moreover, *ca* 5% of dominant females lost dominancy and become subordinates later in their life.

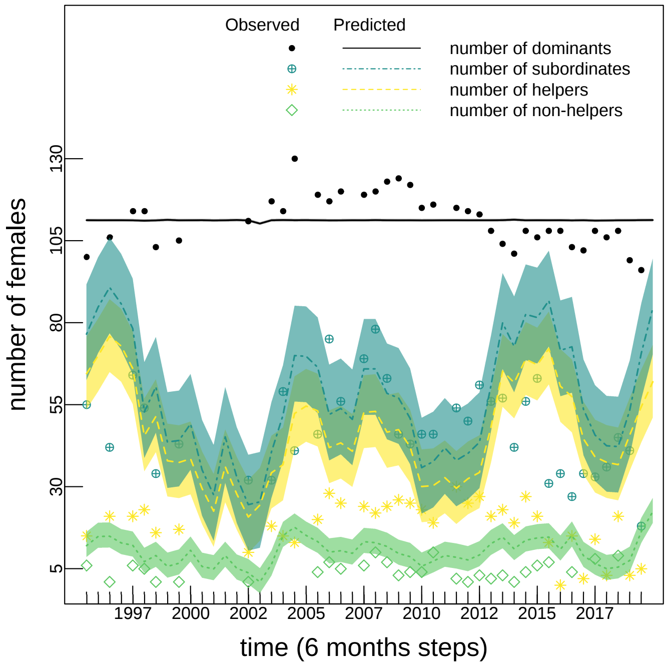

Figure A1. Observed number of dominants (black circles), helpers (yellow stars), non-helpers (green diamonds) and total number of subordinates (blue circle plus) in the female population. Subordinates include helpers, non-helpers and subordinates without behavioural data that could not be classified as either helpers or non-helpers. The lines represent the number predicted by the stochastic matrix population model (SMPM), while shaded areas represent the interquartile ranges. The observed reduction in population size after 2004 and 2011 corresponds to the translocation of 24 and 23 females to other islands for conservation purposes (Wright *et al.*, 2014). In the SMPM the fraction helpers over non-helpers was 4.6 ± 0.01 (mean and standard error, 95% confidence intervals (CI) $[4.4, 4.5]$). The observed fraction of helpers over non-helpers in different seasons was 7.8 ± 1.1 (mean and standard error, 95% CI $[5.67, 9.79]$). However, the observed data are not normally distributed and the median was 5.0 which is roughly similar to the mean value predicted by the SMPM.

A4 STATUSES OF THE OBSERVED FEMALES

Table A1. Statuses of the 958 females observed at least once during the study, and of the 570 females observed seasonally from birth to death during the study. Statuses include helper (h), non-helper (u), and dominant (d). Only 2.9% of the 958 individuals were observed as both helper and non-helper in different seasons (both h and u), while 565 individuals were classified as generic subordinate because of lack of behavioural data necessary for identification of the status. In the table, we report not only the number of individuals observed in a specific status in early life (i.e. six months of life) but also how many of these individuals acquired a dominant position later in their life. Only 13 individuals were able to acquire a dominant position (d) at 6-months of age, so they were ignored in the construction of the population models. Individuals that were observed as both helpers and non-helpers in different seasons and individuals classified as generic subordinates were treated as the status was missing in the statistical analyses.

		h	u	both h and u	Generic subordinate	d
females observed at least in one year	early life	185	68	25	565	115
	acquired dominance	125	52	14	372	
females observed from birth to death	early life	114	28	9	406	13
	acquired dominance	71	21	5	258	

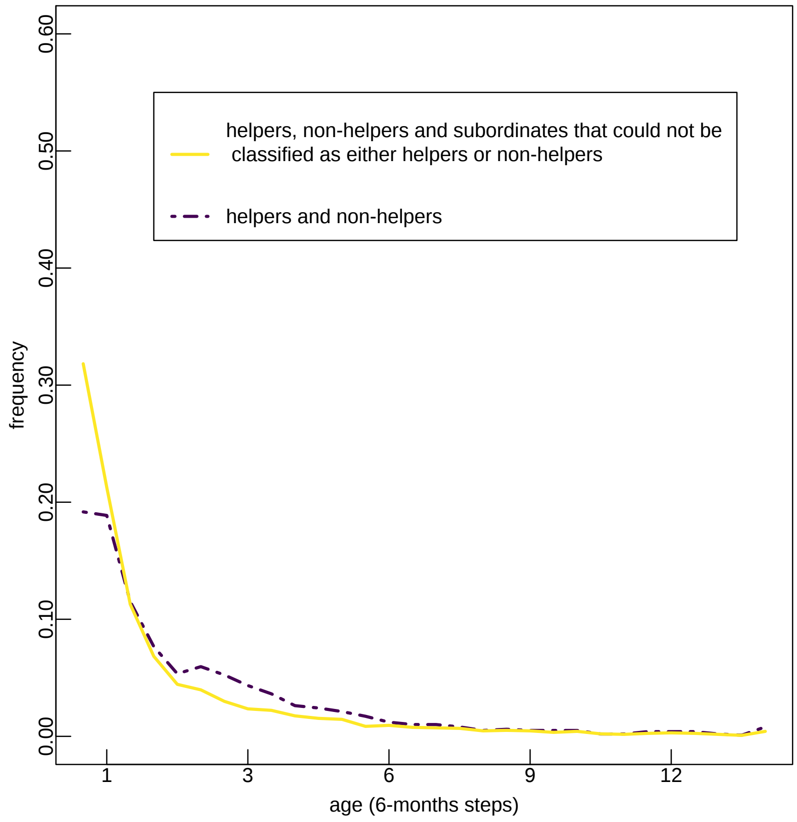

Figure A2. Observed age-distributions of all the observed subordinates (including helpers, non-helpers and subordinates that could not be classified as either helpers or non-helpers because of lack of behavioural observations, yellow line) and of only the individuals that were classified as helpers or non-helpers (purple line). The yellow line has been derived from 2 042 observations, while the purple line from 887 observations.

A5 ALL EXPLANATORY VARIABLES CONSIDERED

In this section we show a list of all the explanatory variables considered to build the statistical analyses of the observed dataset. We used as an explanatory variable the ratio between the number of subordinates over the number of dominants rather than the actual number of individuals because we had missing information on many observed subordinate individuals' status.

Table A2. Explanatory variables considered building the statistical models describing the vital rates. The explanatory variables are: age (a), age squared (a^2), age cubic (a^3), stage class of the subordinate (s either helper or non-helper), standardized population size (N), helper presence (q), ratio between number of helper and non-helpers over the number of dominants (r), stage class of the mother (s_m either dominant or helper), and number of territories with a vacant dominant position (x). When an explanatory variable was considered in the analysis of a vital rate, the corresponding cell includes a checkmark (✓). In the probability of an offspring becoming a helper, age indicates the age of the mother that successfully produced the offspring. Competing models were built with different combinations of the explanatory variables and compared. The final minimal statistical models selected are shown in Table A3. In the reproduction of dominants, we also tested if the effect of helper presence varied between seasons by including multilevel slopes.

	Explanatory variables considered								
Vital Rate	a	a^2	a^3	s	N	q	s_m	r	x
Survival of dominants	✓	✓	✓		✓				
Survival of helpers and non-helpers	✓	✓	✓	✓	✓				
Reproduction of dominants	✓	✓			✓	✓			
Reproduction of helpers	✓	✓			✓				
Probabiliyy of an offspring becoming an helper	✓	✓			✓	✓	✓	✓	✓
Probability of dominants to receive help	✓	✓						✓	
Probability of a helper to acquire a dominant position	✓							✓	✓
Probability of a non-helper to acquire a dominant position	✓							✓	✓

A6 MINIMAL STATISTICAL MODELS

In this section, we report the statistical models describing the vital rates in the observed data and the individual-based model (IBM). Code used to estimate the parameter values can be found on Gitlab and Dataverse.

Table A3. Minimal statistical models and parameter estimates describing the relationship between vital rates and explanatory variables in the observed data. The model type indicates if the model was evaluated with time-varying intercepts (multilevel) or with a time-invariant intercept (one level). The predicted rates are the survival probability of dominants $S(d,a,N,t)$, survival probability of helpers and non-helpers $S(s,a,N,t)$, recruitment probability of the dominants $R(d,a,N,p,t)$, recruitment probability of the helpers $R(h,a,N,t)$, probability of an offspring becoming an helper $f(a,N,t)$, and the probability of a dominant to receive help during reproduction $p(a,r)$. High-posterior density intervals (HPDI) at 89% interval are reported between parenthesis in the subscripts for each estimate. n is the corresponding sample size. The term ne is the minimum effective number of samples with a confidence level and tolerance of 0.05, and it was calculated for each model with the function minESS from the mcmcse library (Flegal *et al.*, 2020). $Logit(y)$ indicates binomial regression using the logit link. The explanatory variables are: age (a), age squared (a^2), standardized population size (N), helper presence (q), ratio between number of helper and non-helpers over the number of dominants (r), and stage class (s). For the multilevel models, we report the mean intercepts and their variance ($\beta_0[t] \sim normal(\mu,\sigma)$).

**Function**	**Model**	**Type**	**Fitted model**	**n**	**ne**
Survival (dominants)	$S(d,a,N,t) \sim binom(1,p_{S_t})$	multilevel	$logit(p_{S_t}) = \beta_0[t] + 0.26_{[0.17,0.34]} * a + -0.02_{[-0.03,-0.02]}) * a^2$ where $\beta_0[t] \sim normal(1.84_{[1.55,2.14]}, .65_{[0.50,0.82]})$	4489	8550
Survival (helpers and non-helpers)	$S(s,a,N,t) \sim binom(1,p_{S_t})$	multilevel	$logit(p_{S_t}) = \beta_0[t] + -0.28_{[-1.12,0.58]} * s + (0.13_{[-0.13,0.47]} * s - 0.11_{[-0.18,-0.03]}) * a + -0.07_{[-0.19,0.04]}) * N$ where $\beta_0[t] \sim normal(3.08_{[2.61,3.61]}, 0.93_{[0.56,1.40]})$	2108	

Continued on next page

where n=sample size; a=age, a^2=age^2; r=ratio between number of h & u over the number of d; q= binary variable binomially distributed as $p(a,r)$ (q=1 if the dominant receives help, q=0 otherwise); s=binary variable indicating if an individual is h (s=0) or u (s=1); N=population size.

Table A3 – *Continued from previous page*

Function	Model	Type	Fitted model	n	ne
Reproduction (dominants)	$R(d,a,N,q,t) \sim binom(1,p_{Rd_t})$	multilevel	$logit(p_{Rd_t}) = \beta_0[t] + 0.73_{[-0.10,1.53]} * q + (-0.26_{[-0.52,0.01]} * q + 0.29_{[0.17,0.41]}) * a + (0.02_{[0.00,0.04]} * q - 0.03_{[-0.04,-0.02]}) * a^2 - 0.20_{[-0.414,0.02]} * N$ where $\beta_0[t] \sim normal(-2.66_{[-3.02,-2.30]}, 0.62_{[0.46,0.80]})$	3762	8461
Reproduction (helpers)	$R(h,a,N,t) \sim binom(1,p_{Rh_t})$	multilevel	$logit(p_{Rh_t}) = \beta_0[t] + 0.86_{[0.47,1.28]} * a - 0.09_{[-0.13,-0.04]} * a^2 - 0.16_{[-0.57,0.28]} * N$ where $\beta_0[t] \sim normal(-4.40_{[-5.35,-3.54]}, 0.96_{[0.50,1.48]})$	709	8426
Offspring becoming a helper	$f(a,N,t) \sim binom(1,p_{f_t})$	multilevel	$logit(p_{f_t}) = \beta_0[t] + 0.17_{[0.04,0.30]} * a$ where $\beta_0[t] \sim normal(0.27_{[-0.39,0.96]}, 0.65_{[0.15,1.20]})$	164	8485
Probability of dominants to receive help	$p(a,r) \sim binom(1,p_p)$	onelevel	$logit(p_p) = -4.83_{[-5.21,-4.45]} + 0.42_{[0.33,0.50]} * a - 0.02_{[-0.03,-0.01]} * a^2 + 7.04_{[5.86,8.23]} * r$	4700	8431

Continued on next page

where n=sample size; a=age, a^2=age^2; r=ratio between number of h & u over the number of d; q= binary variable binomially distributed as $p(a,r)$ (q=1 if the dominant receives help, q=0 otherwise); s=binary variable indicating if an individual is h (s=0) or u (s=1); N=population size.

Table A3 – *Continued from previous page*

**Function**	**Model**	**Type**	**Fitted model**	**n**	**ne**
Probability of a helper to acquire a dominant position	$g_{\text{h}}(h,x,t) \sim binom(1,p_h)$	onelevel	$log(p_h) = min\left[1, -0.55_{[-0.26,-0.84]} + log(x) - log\left[e^{-0.55_{[-0.26,-0.84]}} \sum(S(h,a,N,t)\,h(a,t)) + \sum(S(u,a,N,t)\,u(a,t))\right]\right]$	648	-
Probability of a non-helper to acquire a dominant position	$g_{\text{u}}(u,x,t) \sim binom(1,p_u)$	onelevel	$log(p_u) = min\left[1, log(x) - log\left[e^{-0.55_{[-0.26,-0.84]}} \sum(S(h,a,N,t)\,h(a,t)) + \sum(S(u,a,N,t)\,u(a,t))\right]\right]$	648	-

where n=sample size; a=age, a^2=age^2; r=ratio between number of h & u over the number of d; q= binary variable binomially distributed as $p(a,r)$ (q=1 if the dominant receives help, q=0 otherwise); s=binary variable indicating if an individual is h (s=0) or u (s=1); N=population size.

Some models included variables with missing data points. Population size and the ratio between the number of helpers and non-helpers over the number of dominants were missing in six seasons. They were imputed as if they were missing at random. We assumed that these missing data points were normally distributed.

Because of a lack of behavioural information, we could not record the individuals' stage in the model of the survival probability of subordinate helpers and non-helpers for 565 individuals (Fig. A3). These missing data were binomially distributed because they could only take two possible values (either the individual was a helper (0) or a non-helper (1)). The likelihood of an individual to survive S, unconditional on the stage s was:

$$\begin{aligned} Pr(S) = &(\text{probability an individual is a non-helper}) \times \\ &(\text{probability to survive when an individual is a non-helper}) \\ &+ (\text{probability an individual is a helper}) \times \\ &(\text{probability to survive when an individual is a helper}) \end{aligned} \quad (30)$$

In the analysis, the probability that the status was non-helper when the status was known followed the Bernoulli distribution. This value informs the prior for the missing information.

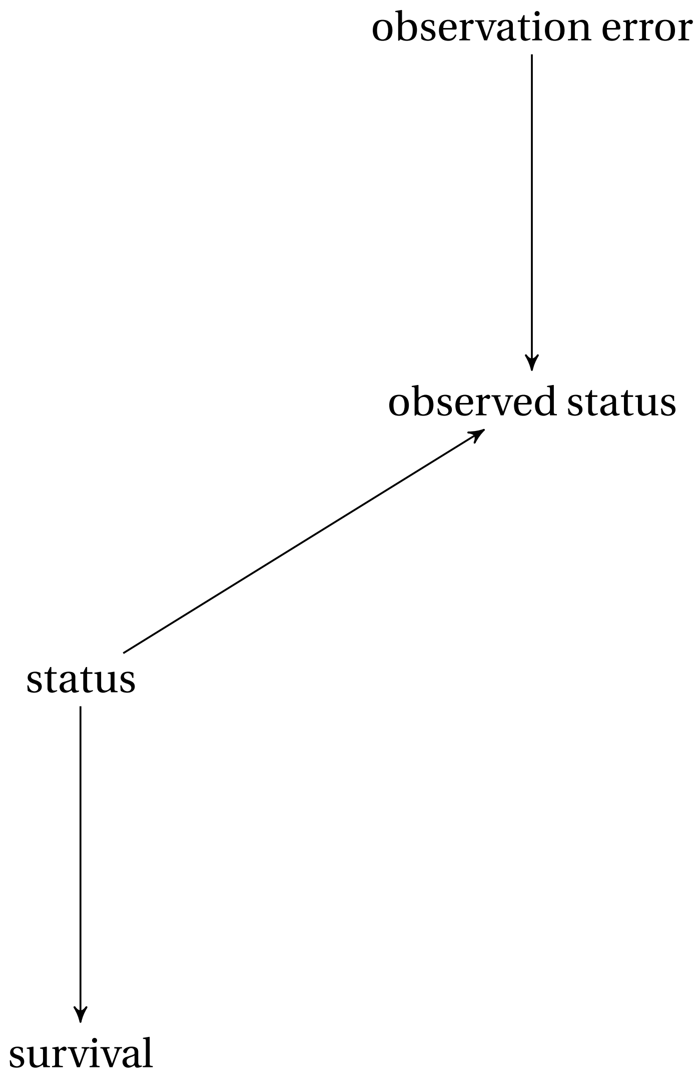

Figure A3. Mechanism describing why there are missing data in the status of subordinates (helpers and non-helpers). The status of an individual affects the survival probability. Because of observation error, we were often unable to determine if a subordinate was a helper or a non-helper. Therefore the observed status was determined both by our observation error and the status of the individual. Unknown statuses arose mainly after nest failures during nest-building or incubation or because field-work was not conducted. We could determine which individual was dominant in a territory by observing pair-bonding behaviour with the dominant male. Therefore, when the status was unobserved, we could assume it was either a helper or non-helper (i.e. a binomial variable). The unobserved status could influence the survival probability because helpers could have lower mortality risks (Kingma (2017), but see Crick (1992), which reports that helpers could incur higher mortality).

Table A4. Parameter estimates for imputed data in the statistical models describing the relationship between vital rates and explanatory variables. Variables including missing data points were the population size (N), ratio between number of h & u over the number of d (r), and the status of subordinate individuals (s). We assumed that data were missing at random. The predicted rates containing missing data are the survival probability of helpers and non-helpers $S(s,a,N,t)$, recruitment probability of the dominants $R(d,a,N,p,t)$, recruitment probability of the helpers $R(h,a,N,t)$, and the probability of a dominant to receive help during reproduction $p(a,r)$. The terms n_{obs} and n_{miss} are the corresponding number of observed and missing data points. The term $norm$ indicates that imputed data are normally distributed, while $binom$ indicates the binomial distribution. High-posterior density intervals (HPDI) at 89% interval are reported between parenthesis in the subscripts for each estimate.

**Function**	**Model**	**Variable**	**Imputation**	n_{obs}	n_{miss}
Survival (helpers and non-helpers)	$S(s,a,N,t)$	N	$\sim norm(\mu = 0.24_{[0.20,0.27]}, \sigma = 1.00_{[0.98,1.03]})$	1964	144
Survival (helpers and non-helpers)	$S(s,a,N,t)$	s	$\sim binom(1, p = 0.17_{[0.15,0.20]})$	885	1223
Reproduction (dominants)	$R(d,a,N,q,t)$	N	$\sim norm(\mu = 0.34_{[0.31,0.36]}, \sigma = 0.91_{[0.90,0.93]})$	3597	165
Reproduction (helpers)	$R(h,a,N,t)$	N	$\sim norm(\mu = 0.35_{[0.30,0.41]}, \sigma = 0.90_{[0.86,0.94]})$	699	10

Continued on next page

where n_{obs}=number of observed data points, n_{miss}=number of missing data points; r=ratio between number of h & u over the number of d; s=binary variable indicating if an individual is h (s=0) or u (s=1); N=population size.

Table A4 – *Continued from previous page*

**Function**	**Model**	**Variable**	**Imputation**	n_{obs}	n_{miss}
Probability of dominants to receive help	$p(a,r)$	r	$\sim norm(\mu = 0.20_{[0.19,0.20]}, \sigma = 0.08_{[0.08,0.08]})$	4091	609

where n_{obs}=number of observed data points, n_{miss}=number of missing data points; r=ratio between number of h & u over the number of d; s=binary variable indicating if an individual is h (s=0) or u (s=1); N=population size.

A7 PRIORS IN BAYESIAN STATISTICS

Bayesian analyses incorporate prior information of the data via conditional probabilities (Bayes' rule). This prior information is updated thanks to the information given by the actual data. The algorithm then computes a posterior distribution, which represents the output of the statistical analyses. How to choose priors has been a matter of debate among statisticians. Regularizing or weakly regularizing priors for a parameter reduce overfitting while the model incorporates information from the sampling. Therefore, inferences for parameters are kept in a reasonable range of the parameter space. Regularizing or weakly regularizing priors can be preferred to non-informative priors because they can mitigate type I and type M errors (Lemoine, 2019). For a thorough explanation of the use of priors in Bayesian statistics, see Gelman *et al.* (2013).

We also tested non-informative priors but the results were comparable. We do not report these results for brevity.

A8 POSTERIOR PREDICTIVE CHECKS: PREDICTIVE PLOTS

Plots of the raw data against model predictions to assess model fit. We added random noise to the observed data points to facilitate the interpretation of the plots.

Figure A4. Raw data and predictions of the survival probability of dominant females as a function of age. Solid black lines with circles represent the maximum *a posteriori* estimate of the mean survival of dominants. The shaded regions represent 89% prediction interval in the population at each age. Age is measured at intervals of six-months time steps. Breeding seasons occur every six-months each year. The main breeding seasons correspond to the months of June-September of a given year, while the minor breeding season corresponds to January-March. Please note that we used the function jitter (from base R Core Team, 2020) to add a little random vertical noise to the data to see individual data points more clearly. All observed values equal 0 or 1 with respect to the y-axis, even if they appear larger or smaller.

(a)

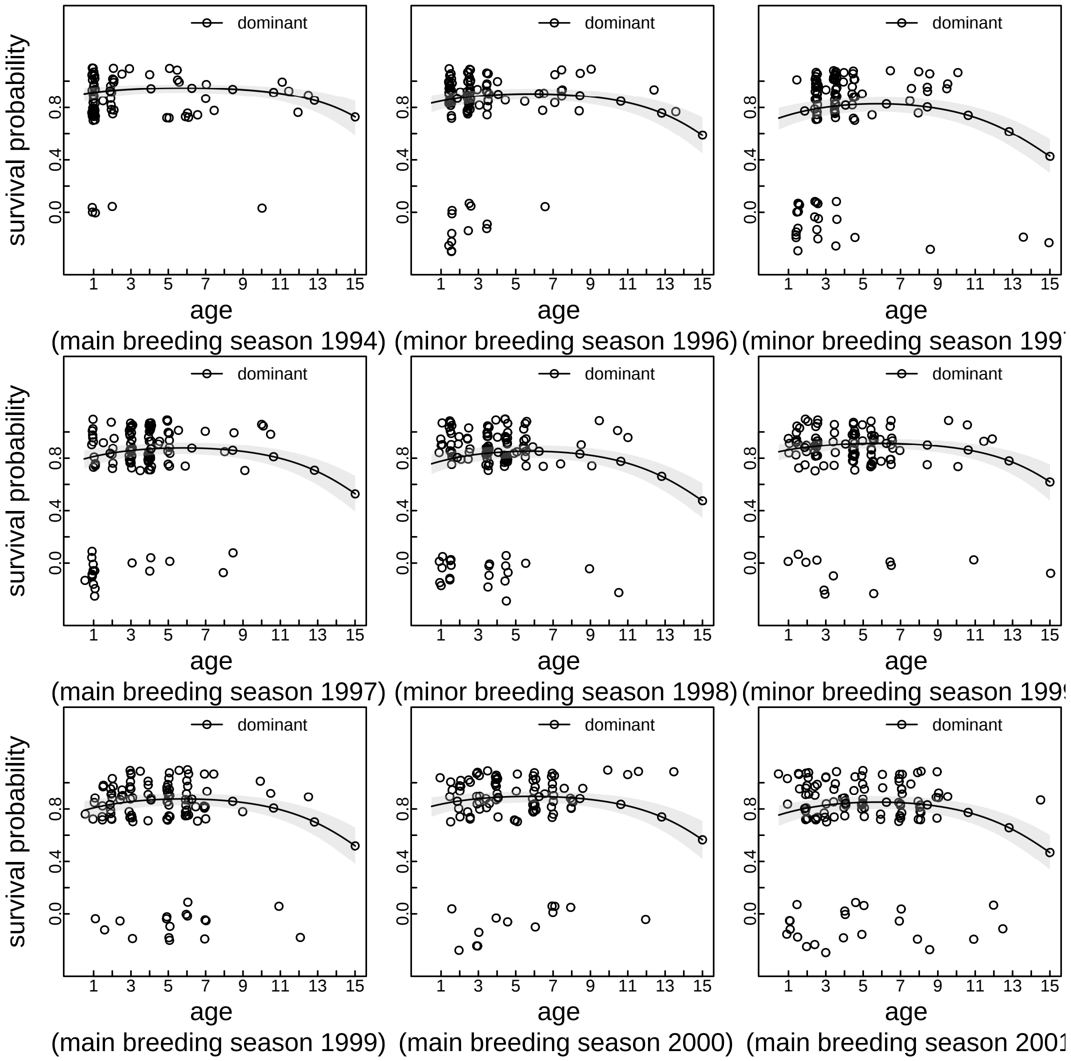

(b)

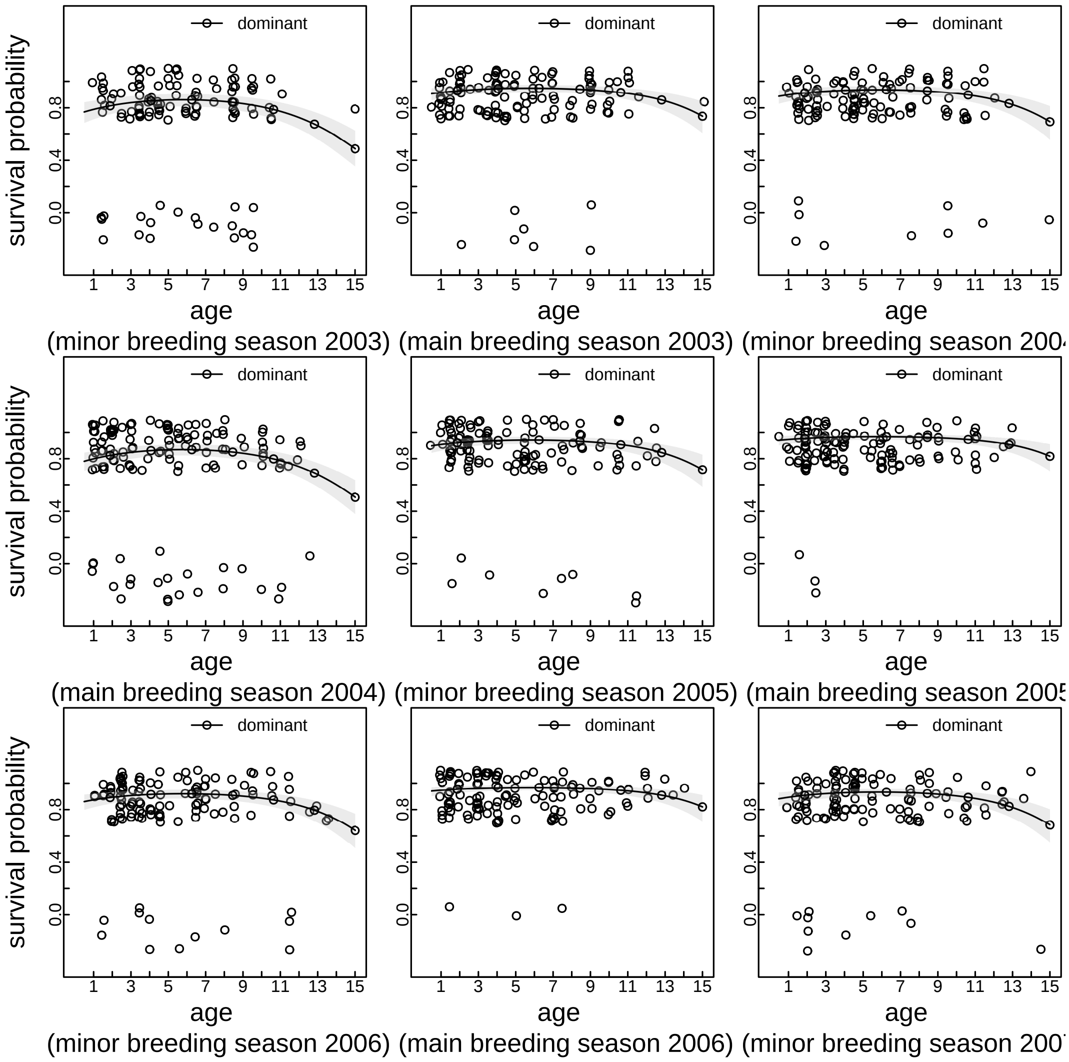

survival probability
dominant
0.0
0.4
0.8
1 3 5 7 9 11 13 15
age
(minor breeding season 2003)
(main breeding season 2003)
(minor breeding season 200
(main breeding season 2004)
(minor breeding season 2005)
(main breeding season 200
(minor breeding season 2006)
(main breeding season 2006)
(minor breeding season 200

(c)

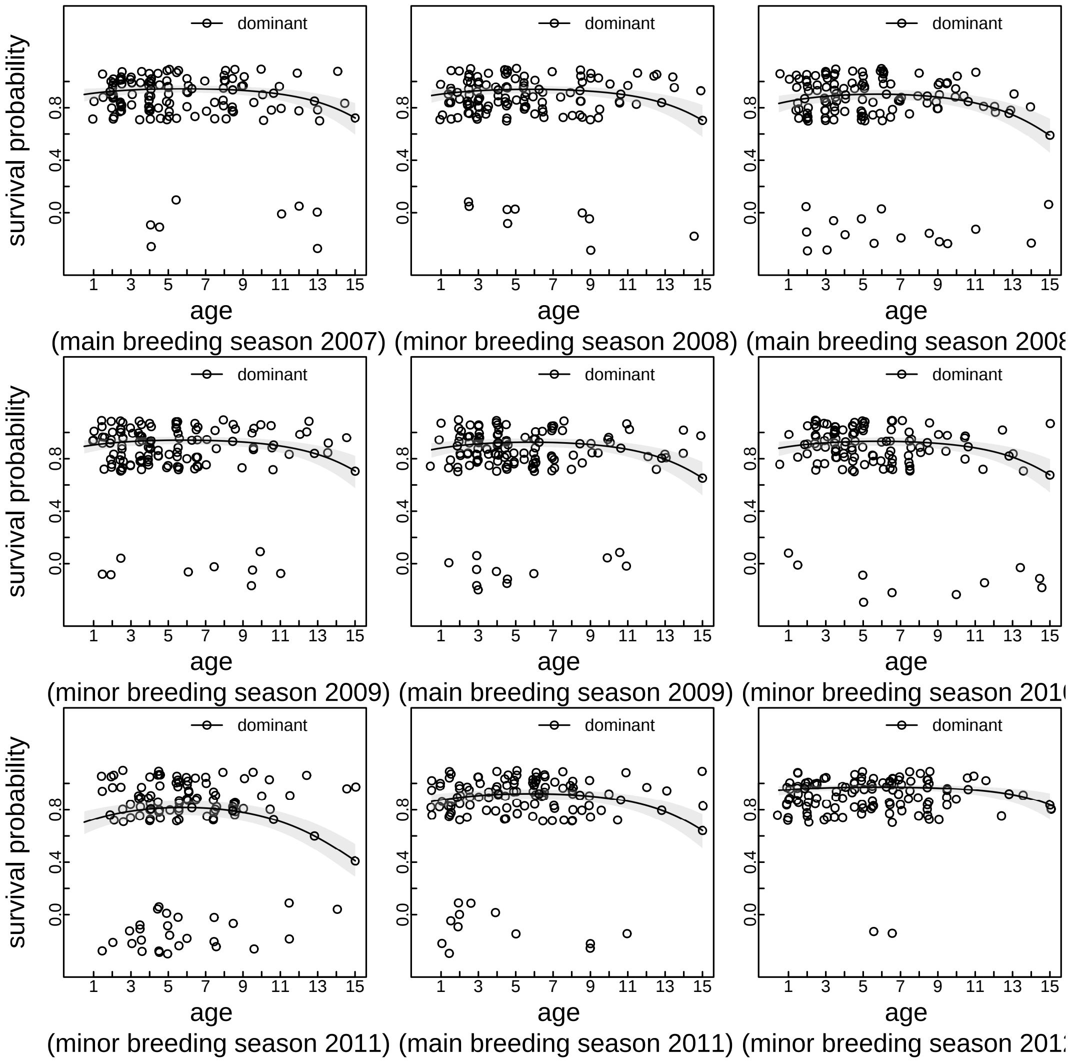
dominant
survival probability
age
(main breeding season 2007)
(minor breeding season 2008)
(minor breeding season 2009)
(main breeding season 2009)
(minor breeding season 2011)
(main breeding season 2011)

(d)

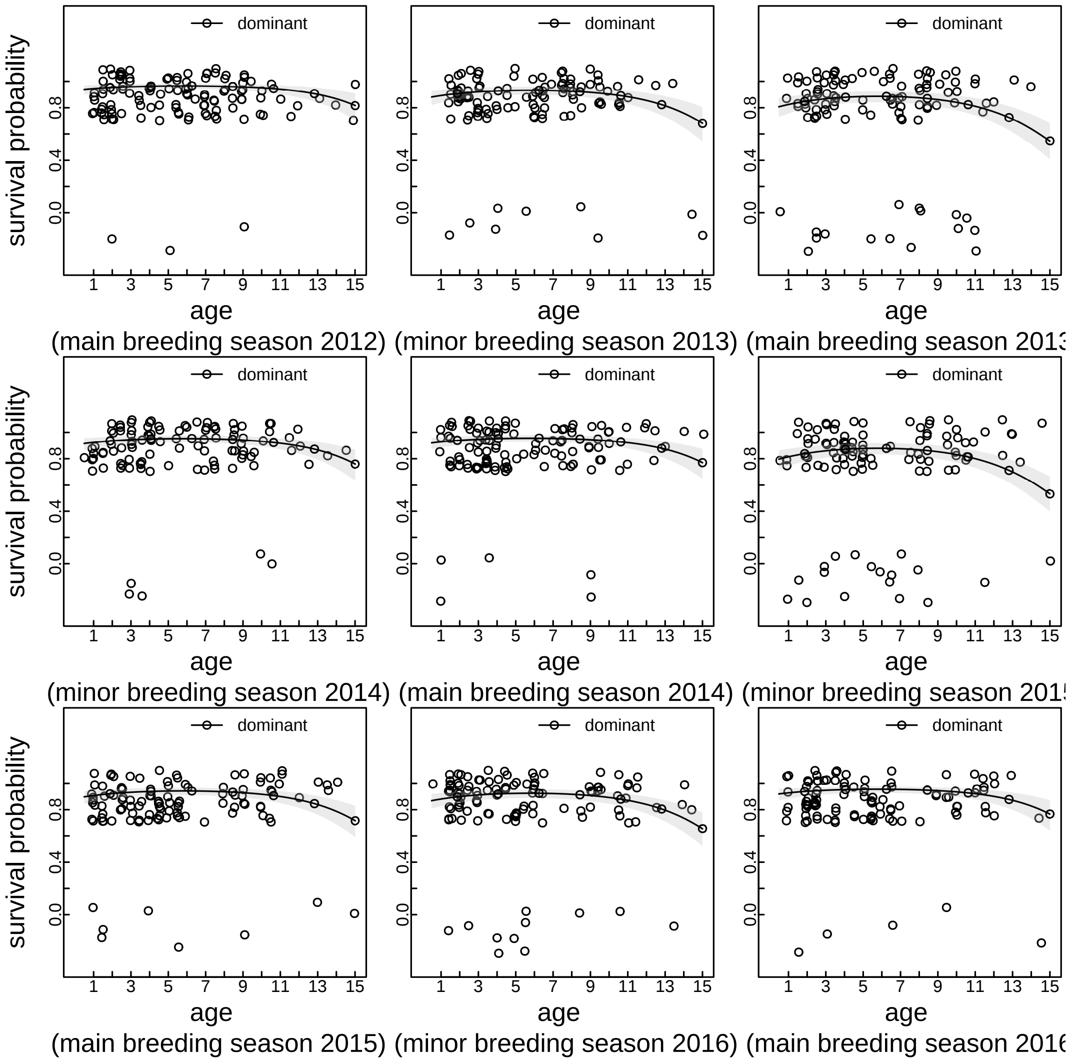

dominant
survival probability
0.0
0.4
0.8
1
3
5
7
9
11
13
15
age
(main breeding season 2012)
(minor breeding season 2013)
(main breeding season 2013
(minor breeding season 2014)
(main breeding season 2014)
(minor breeding season 201
(main breeding season 2015)
(minor breeding season 2016)
(main breeding season 2016

(e)

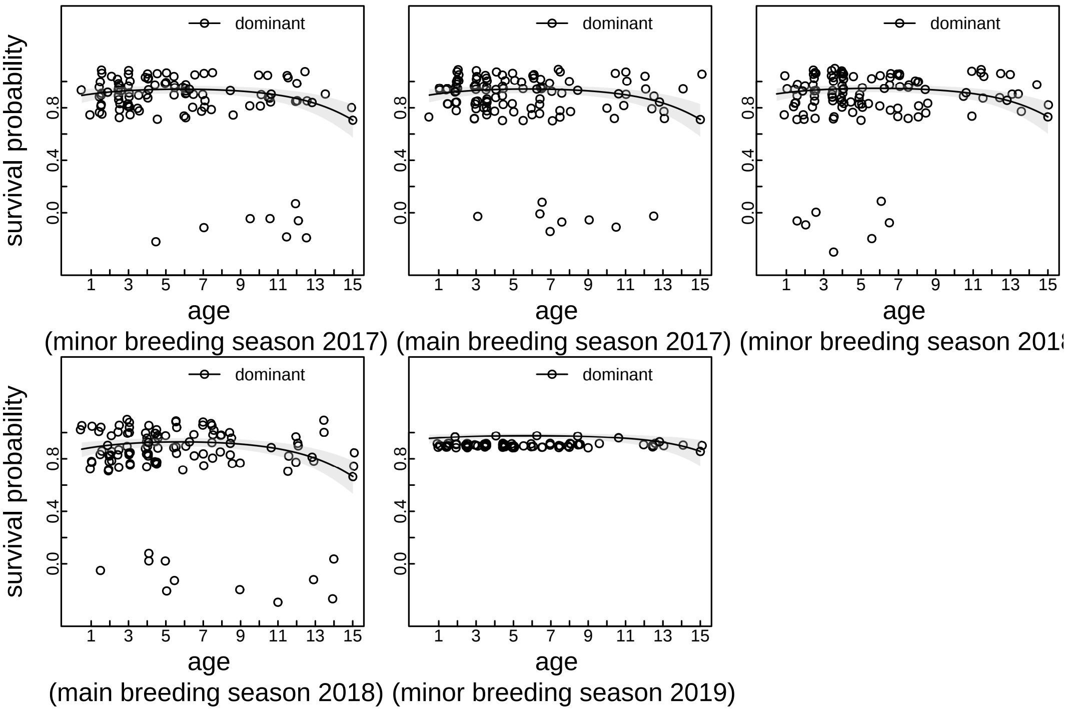
dominant
survival probability
0.0
0.4
0.8
1 3 5 7 9 11 13 15
age
(minor breeding season 2017)
(main breeding season 2017)
(minor breeding season 201
(main breeding season 2018)
(minor breeding season 2019)

Figure A5. Raw data and predictions of the survival probability of helpers and non-helpers as a function of age. Yellow crosses represent helpers, blue diamonds represent non-helpers and purple circles represent subodinates with unknown status. Longdashed yellow lines and dashed blue lines represent the maximum *a posteriori* estimate of the mean survival of helpers, and non-helpers respectively. The shaded regions represent 89% prediction interval in the population at each age. In the plots, the standardized population size was fixed at 0. Age is measured at intervals of six-months time steps. Breeding seasons occur every six-months each year. The main breeding seasons correspond to the months of June-September of a given year, while the minor breeding season corresponds to January-March. Please note that we used the function jitter (from base R Core Team, 2020) to add a little random vertical noise to the data to see individual data points more clearly. All observed values equal 0 or 1 with respect to the y-axis, even if they appear larger or smaller.

(a)

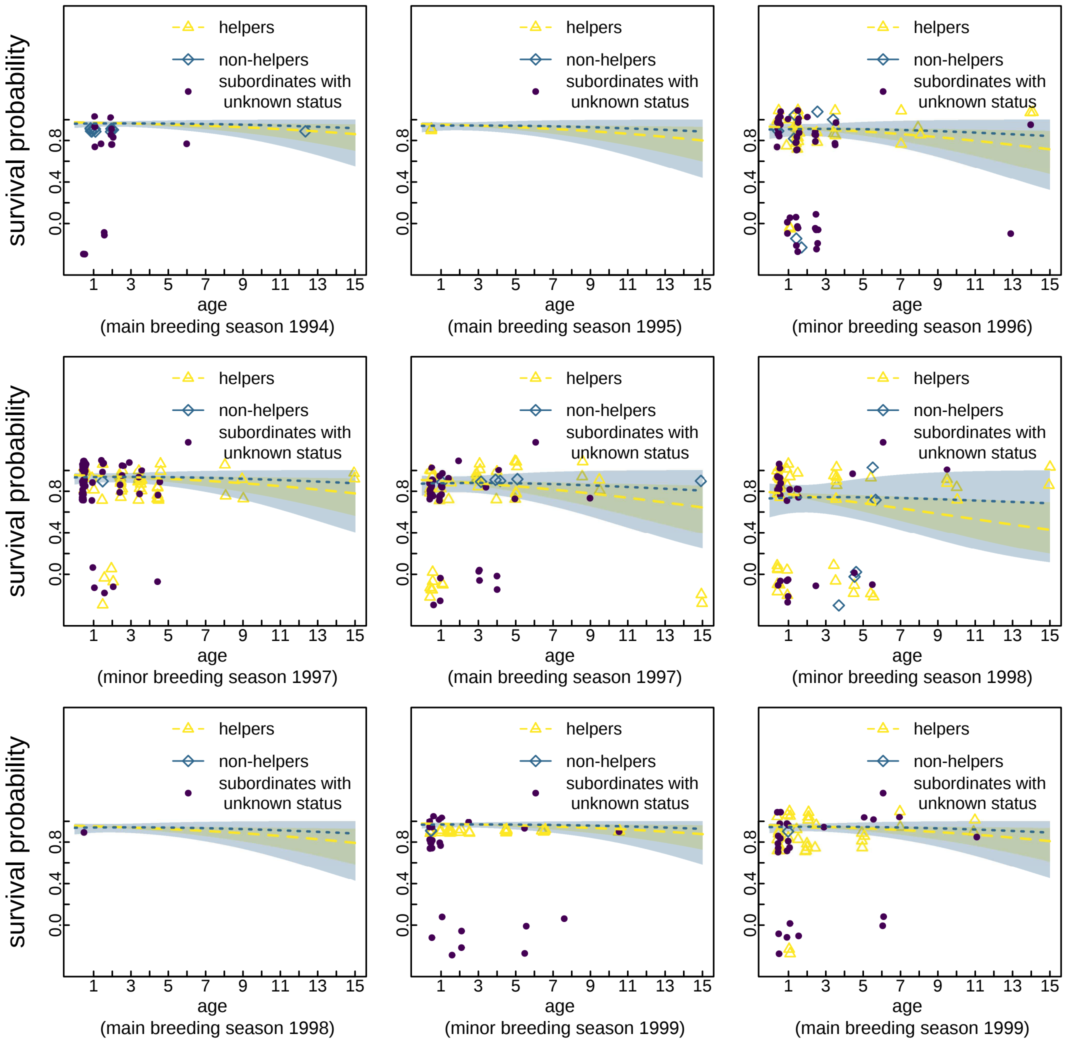

(b)

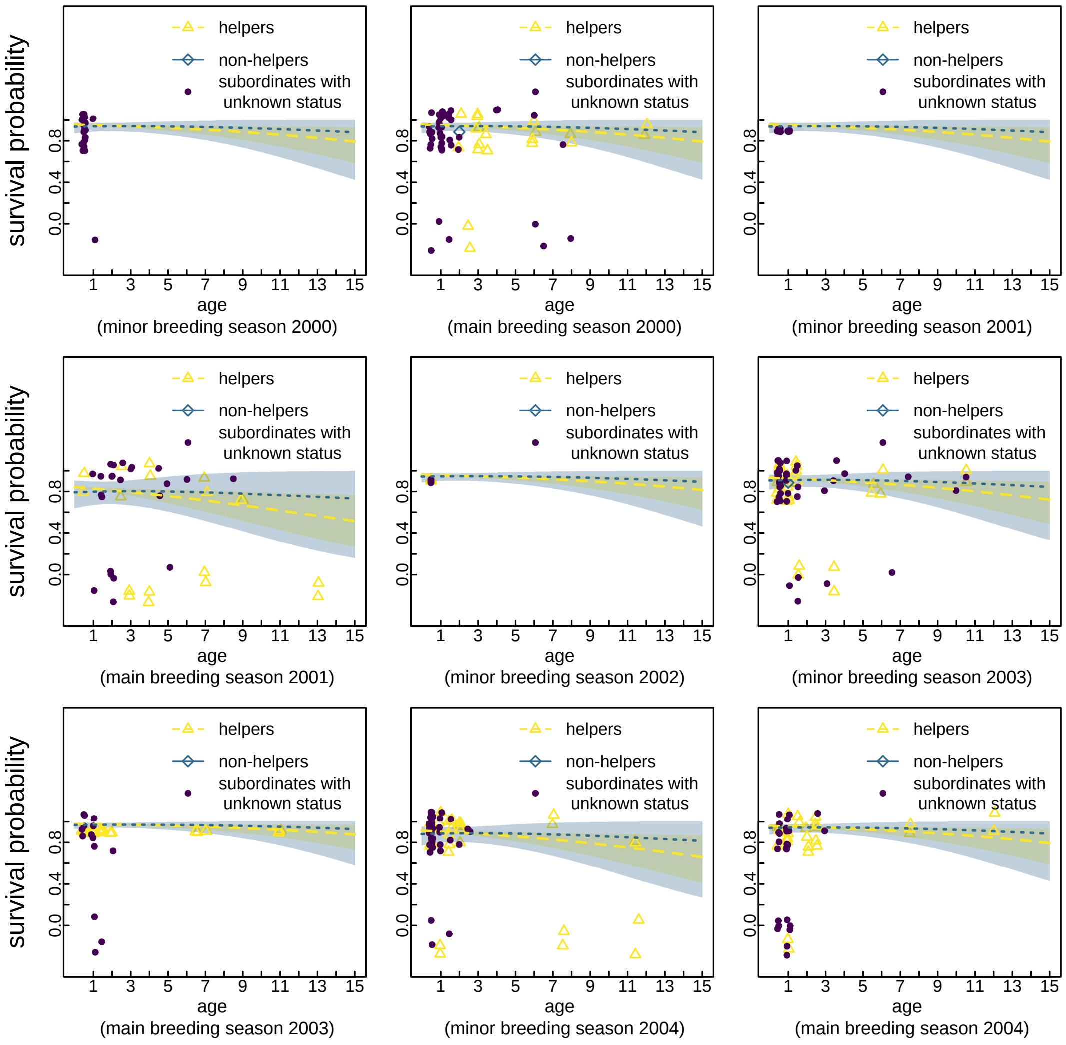

helpers
non-helpers
subordinates with unknown status
survival probability
0.0
0.4
0.8
1 3 5 7 9 11 13 15
age
(minor breeding season 2000)
(main breeding season 2000)
(minor breeding season 2001)
(main breeding season 2001)
(minor breeding season 2002)
(minor breeding season 2003)
(main breeding season 2003)
(minor breeding season 2004)
(main breeding season 2004)

(c)

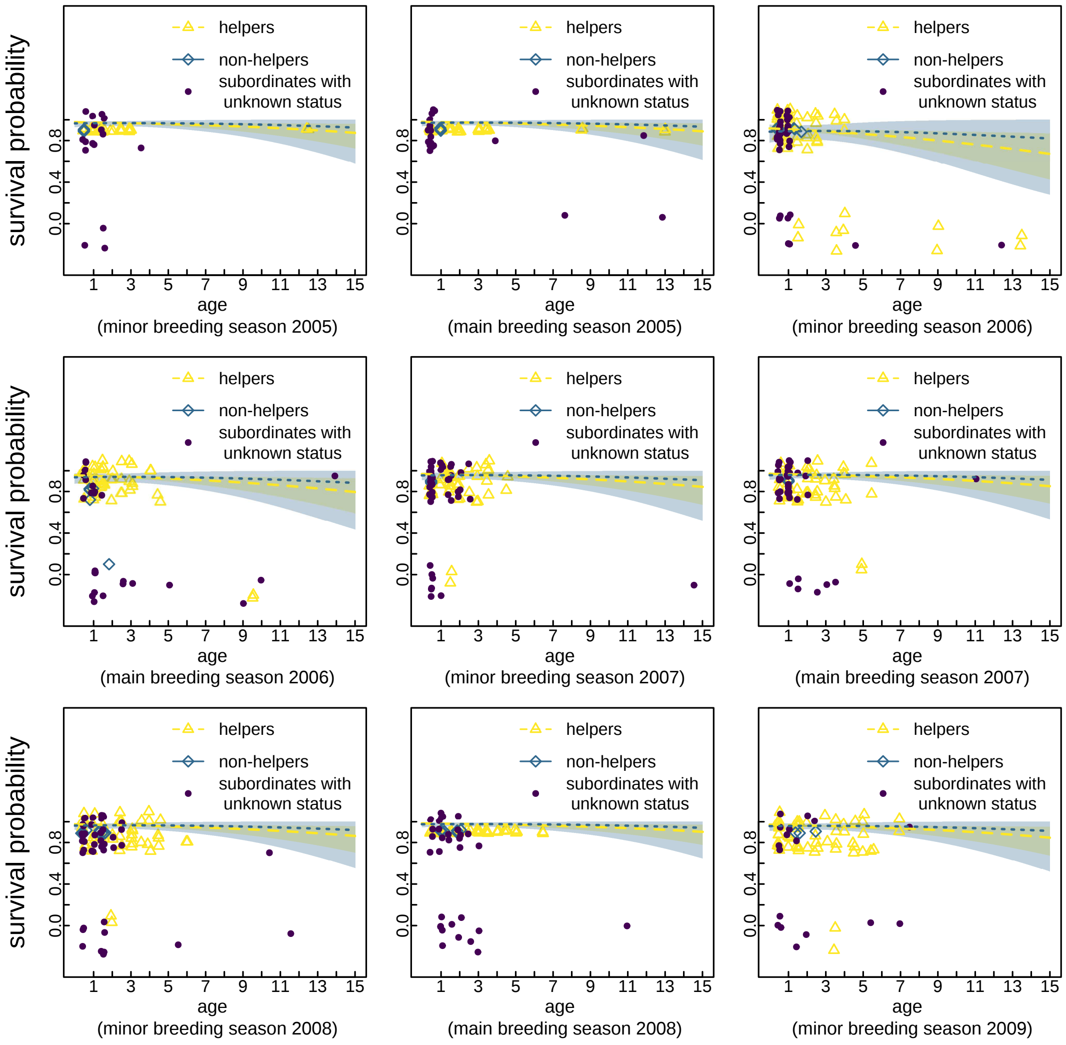
helpers
non-helpers
subordinates with unknown status
survival probability
0.0
0.4
0.8
1
3
5
7
9
11
13
15
age
(minor breeding season 2005)
(main breeding season 2005)
(minor breeding season 2006)
(main breeding season 2006)
(minor breeding season 2007)
(main breeding season 2007)
(minor breeding season 2008)
(main breeding season 2008)
(minor breeding season 2009)

(d)

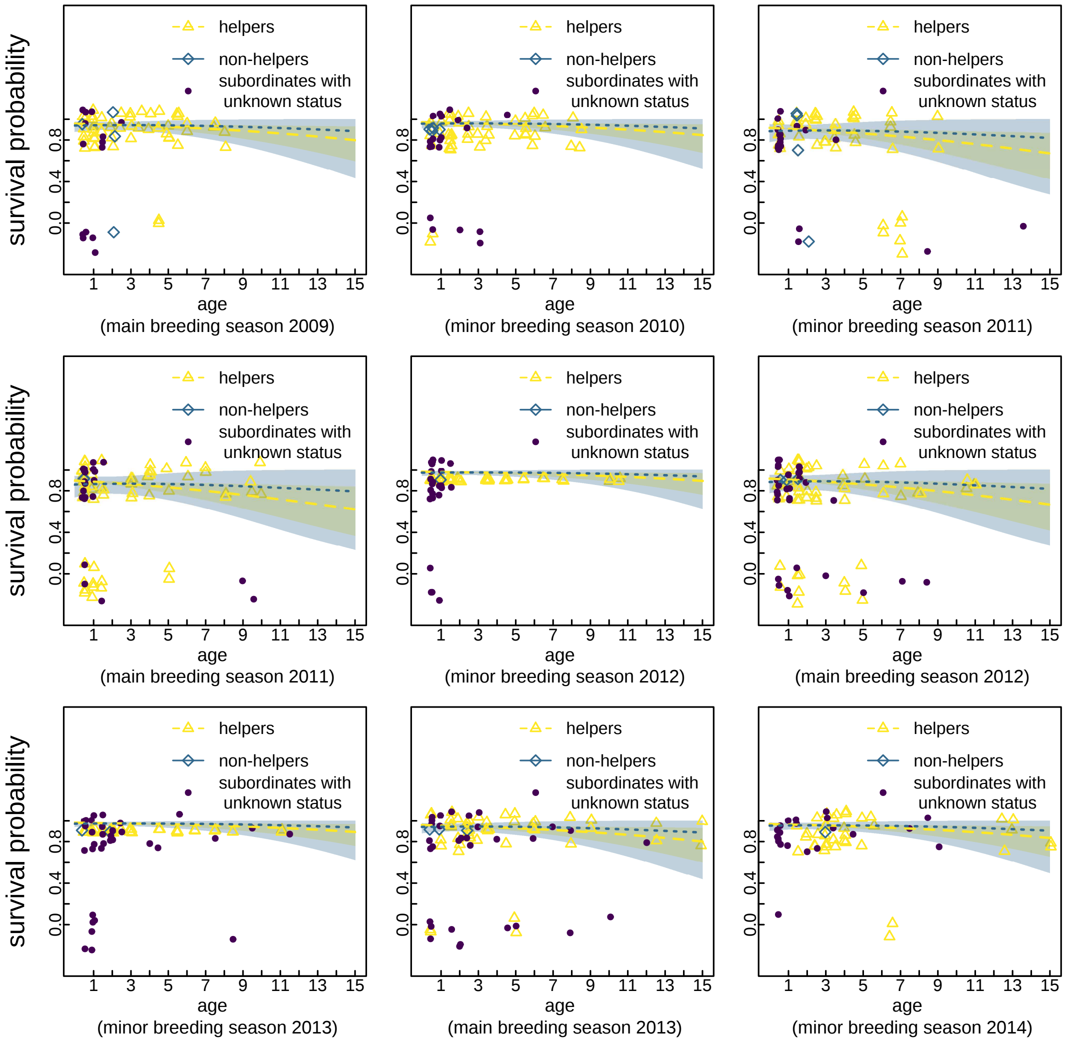
helpers
non-helpers
subordinates with unknown status
survival probability
0.0
0.4
0.8
age
1
3
5
7
9
11
13
15
(main breeding season 2009)
(minor breeding season 2010)
(minor breeding season 2011)
(main breeding season 2011)
(minor breeding season 2012)
(main breeding season 2012)
(minor breeding season 2013)
(main breeding season 2013)
(minor breeding season 2014)

(e)

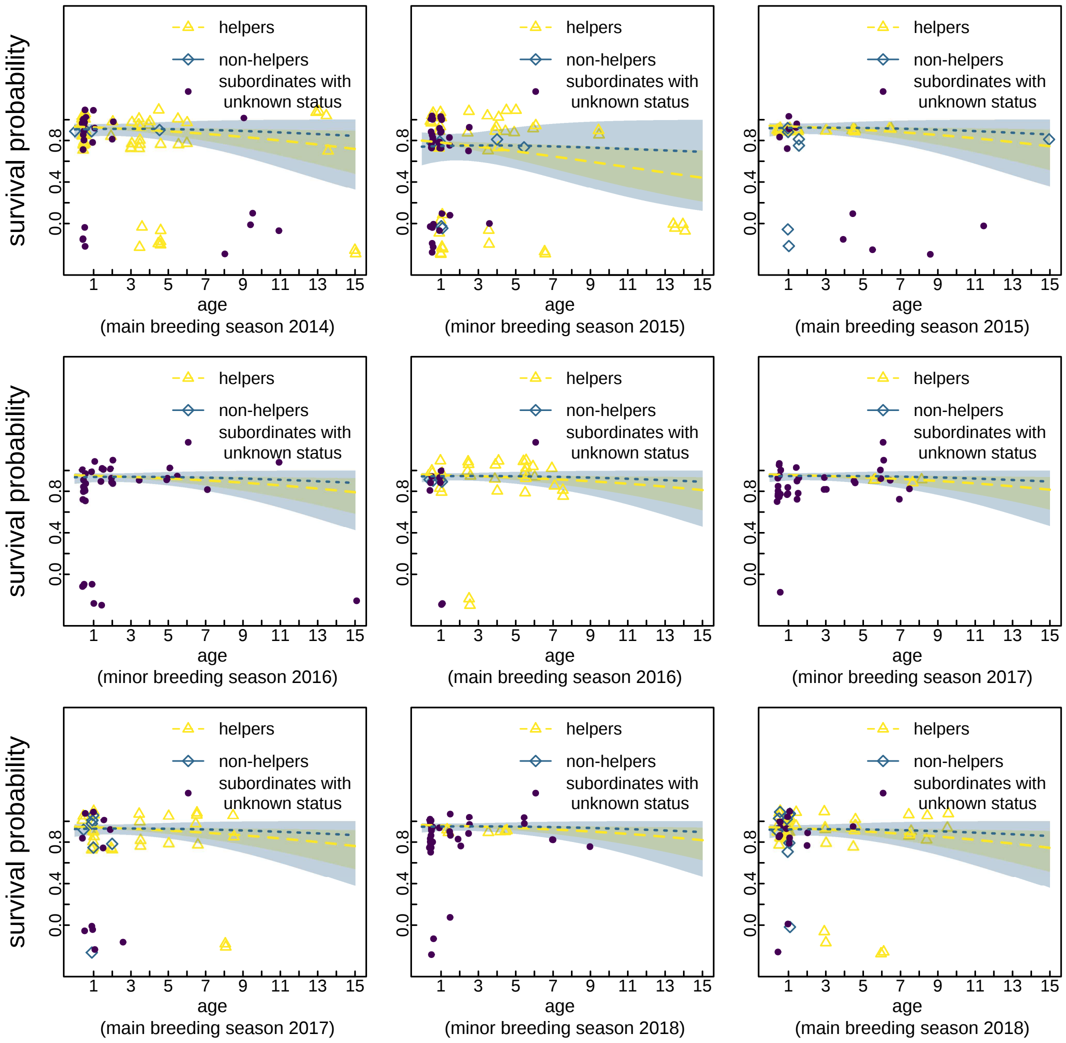
helpers
non-helpers
subordinates with unknown status
survival probability
0.0
0.4
0.8
1
3
5
7
9
11
13
15
age
(main breeding season 2014)
(minor breeding season 2015)
(main breeding season 2015)
(minor breeding season 2016)
(main breeding season 2016)
(minor breeding season 2017)
(main breeding season 2017)
(minor breeding season 2018)
(main breeding season 2018)

(f)

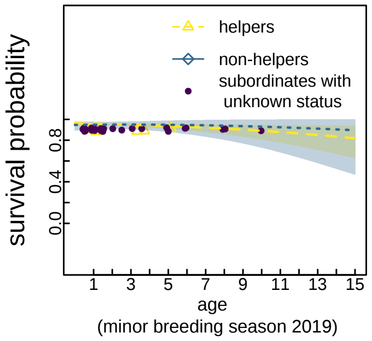
helpers
non-helpers
subordinates with unknown status
survival probability
0.0
0.4
0.8
1
3
5
7
9
11
13
15
age
(minor breeding season 2019)

Figure A6. Raw data and predictions of the survival probability of helpers and non-helpers as a function of the standardized population size. Solid black lines represent the maximum a posteriori estimate of the mean survival of helpers and non helpers during their lifetime. In the plots the age was fixed at two years. Please note that we used the function jitter (from base R Core Team, 2020) to add a little random vertical noise to the data to see individual data points more clearly. All observed values equal 0 or 1 with respect to the y-axis, even if they appear larger or smaller.

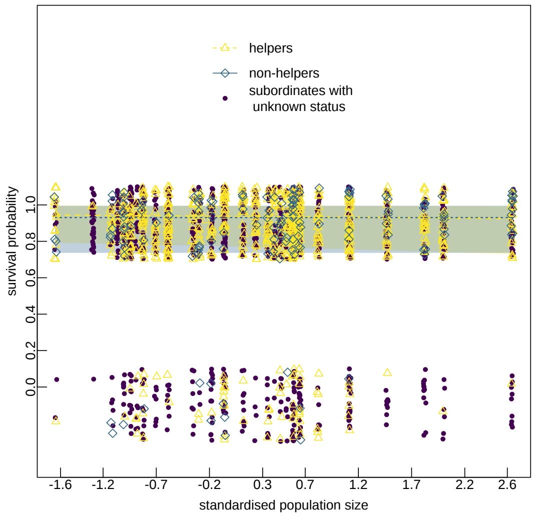

Figure A7. Raw data and predictions of the reproduction probability as a function of age of dominants that do not receive help from a subordinate helper as a function of age (purple squares and dot-dashed lines) and of dominants that receive help from at least a subordinate helper (green triangles and solid lines). Solid lines represent the maximum a posteriori estimate of the mean reproduction probability. The shaded regions represent 89% prediction interval in the population at each age. The standardized population size was fixed at 0. Please note that we used the function jitter (from base R Core Team, 2020) to add a little random vertical noise to the data to see individual data points more clearly. All observed values equal 0 or 1 with respect to the y-axis, even if they appear larger or smaller.

(a)

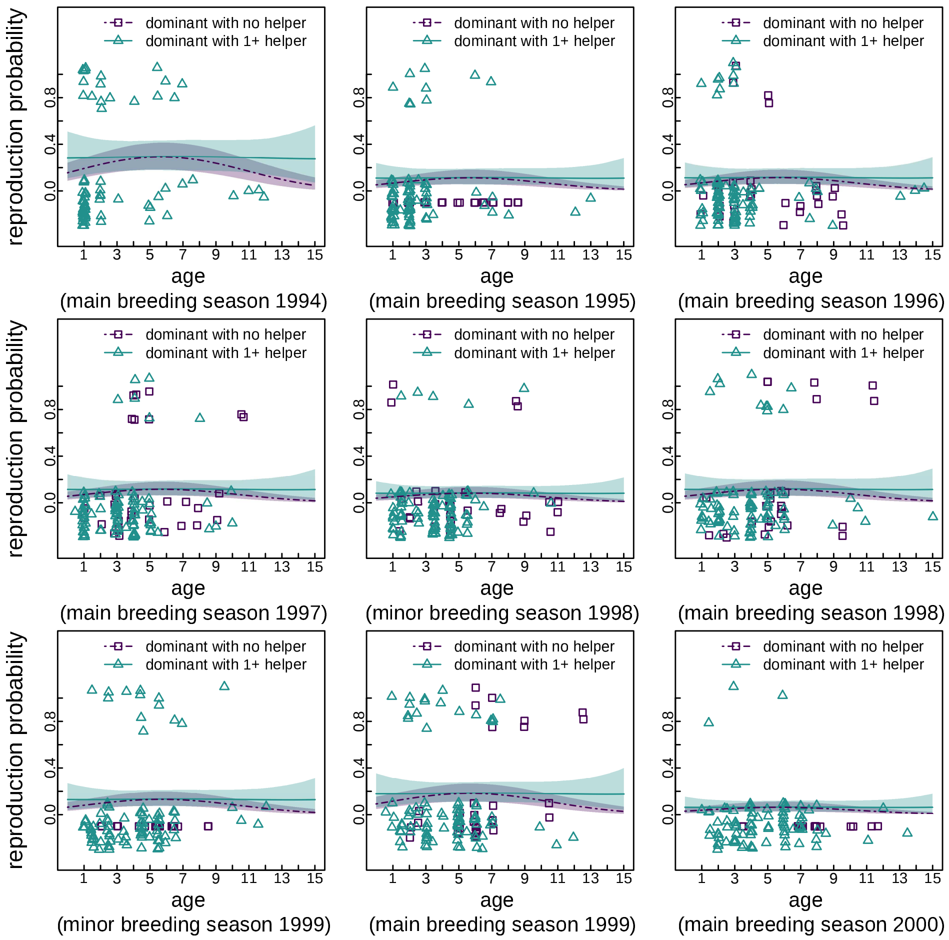

(b)

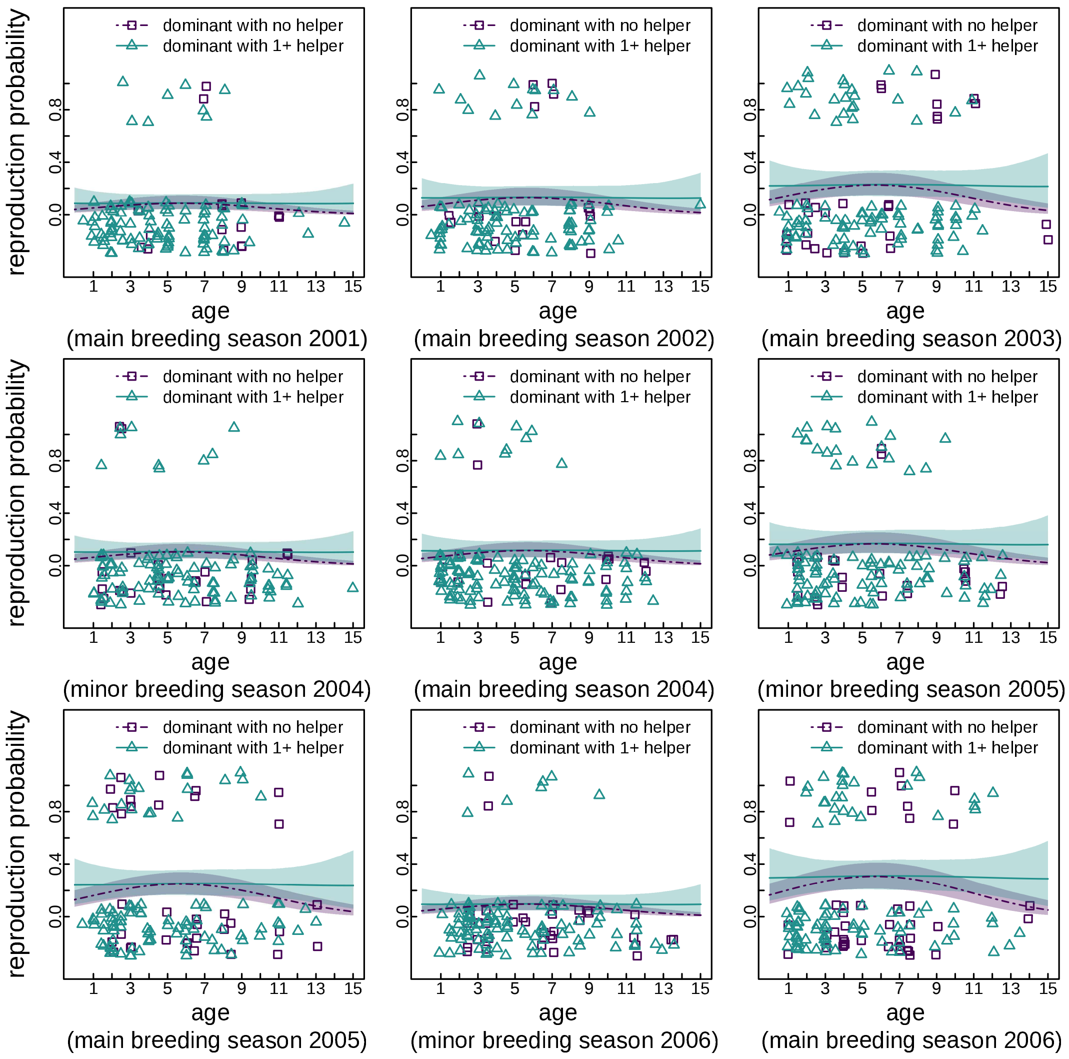
reproduction probability
dominant with no helper
dominant with 1+ helper
0.0
0.4
0.8
1
3
5
7
9
11
13
15
age
(main breeding season 2001)
(main breeding season 2002)
(main breeding season 2003)
(minor breeding season 2004)
(main breeding season 2004)
(minor breeding season 2005)
(main breeding season 2005)
(minor breeding season 2006)
(main breeding season 2006)

(c)

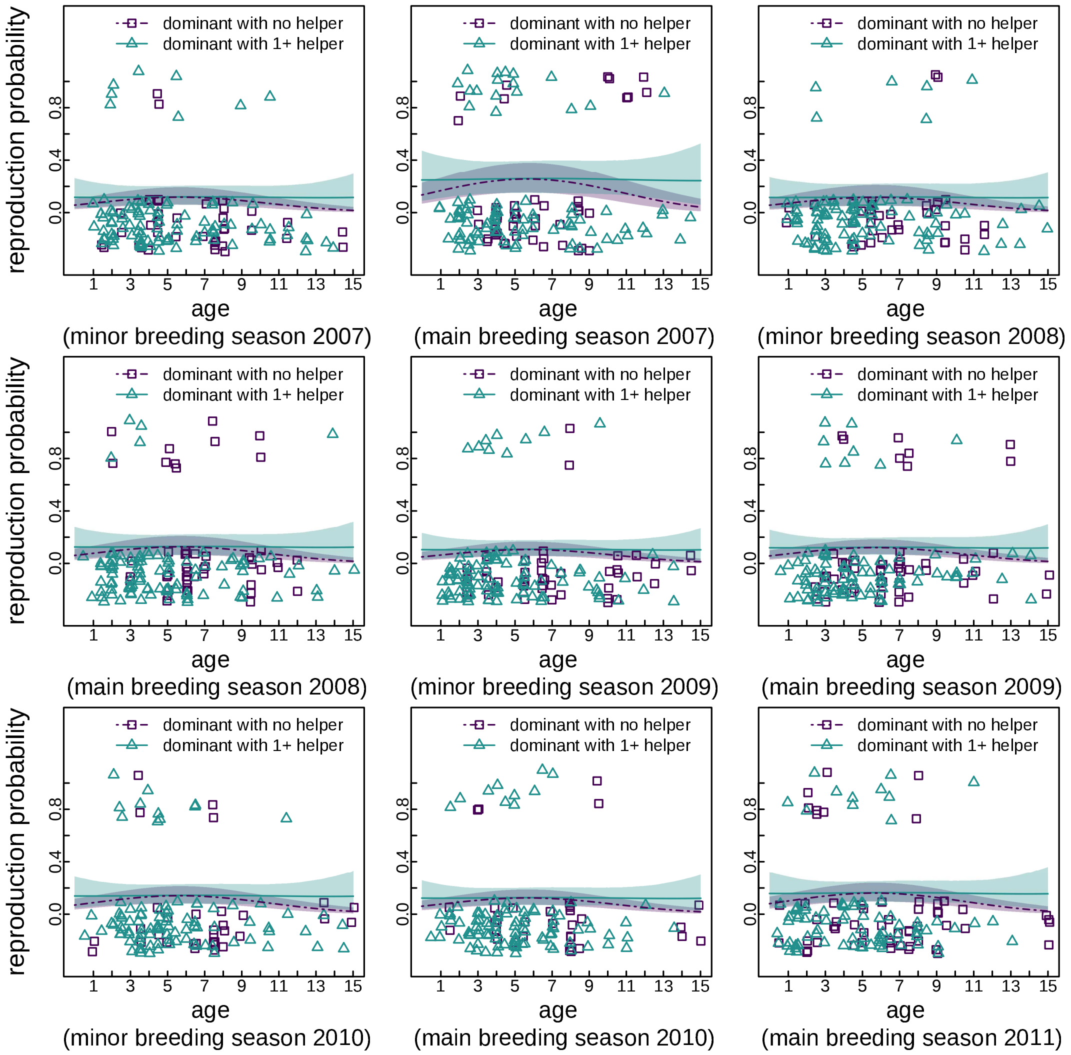

dominant with no helper
dominant with 1+ helper
reproduction probability
0.0
0.4
0.8
1 3 5 7 9 11 13 15
age
(minor breeding season 2007)
(main breeding season 2007)
(minor breeding season 2008)
(main breeding season 2008)
(minor breeding season 2009)
(main breeding season 2009)
(minor breeding season 2010)
(main breeding season 2010)
(main breeding season 2011)

(d)

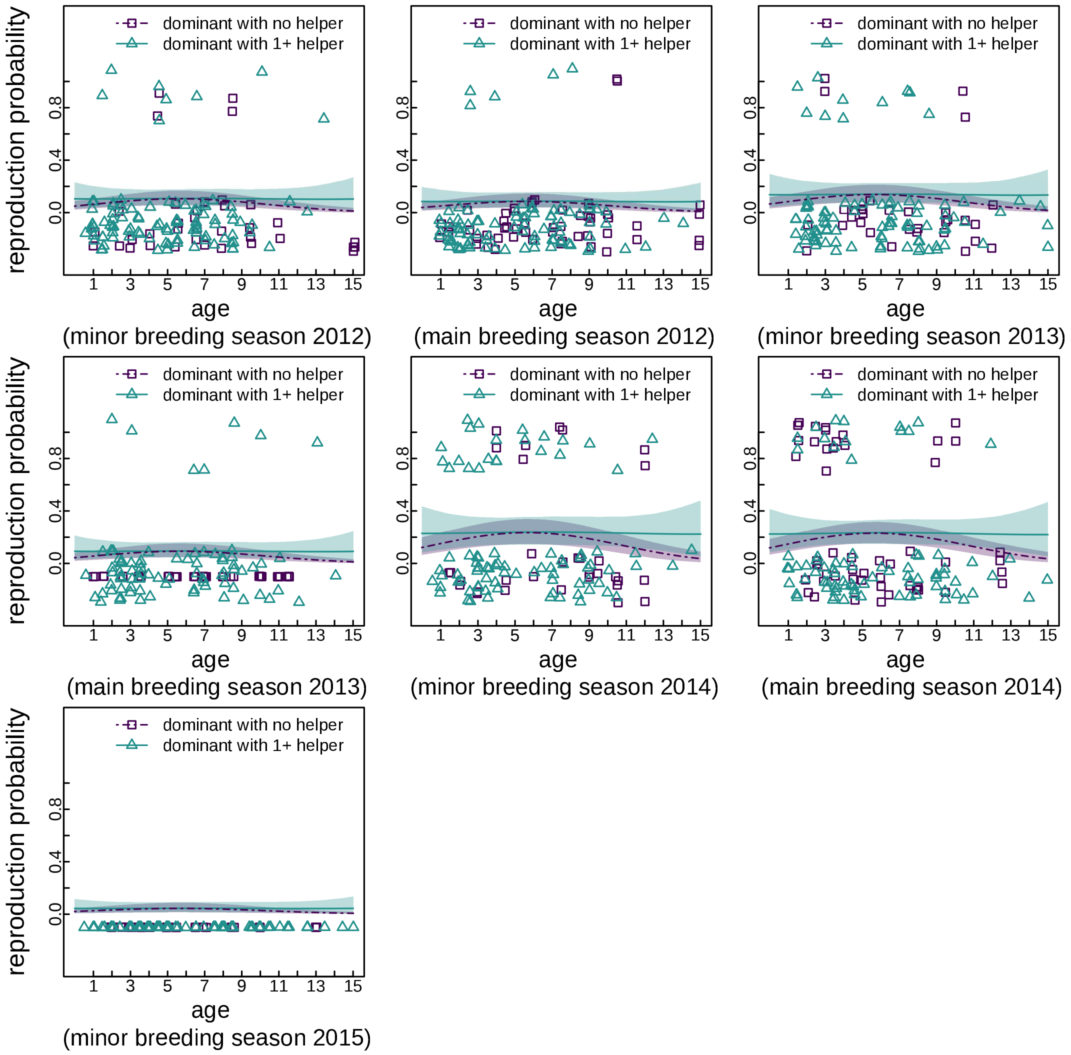
reproduction probability
dominant with no helper
dominant with 1+ helper
0.0
0.4
0.8
1
3
5
7
9
11
13
15
age
(minor breeding season 2012)
(main breeding season 2012)
(minor breeding season 2013)
(main breeding season 2013)
(minor breeding season 2014)
(main breeding season 2014)
(minor breeding season 2015)

Figure A8. Raw data and predictions of the reproduction probability of dominants as a function of the standardized population size. Solid lines represent the maximum a posteriori estimate of the mean reproduction probability in different seasons. The age was fixed at 2. The left-hand side panel represents the reproduction probability of dominants with no helpers (purple), while the right-hand side panel represents the reproduction probability of dominants with one or more helpers (light blue). Please note that we used the function jitter (from base R Core Team, 2020) to add a little random vertical noise to the data to see individual data points more clearly. All observed values equal 0 or 1 with respect to the y-axis, even if they appear larger or smaller.

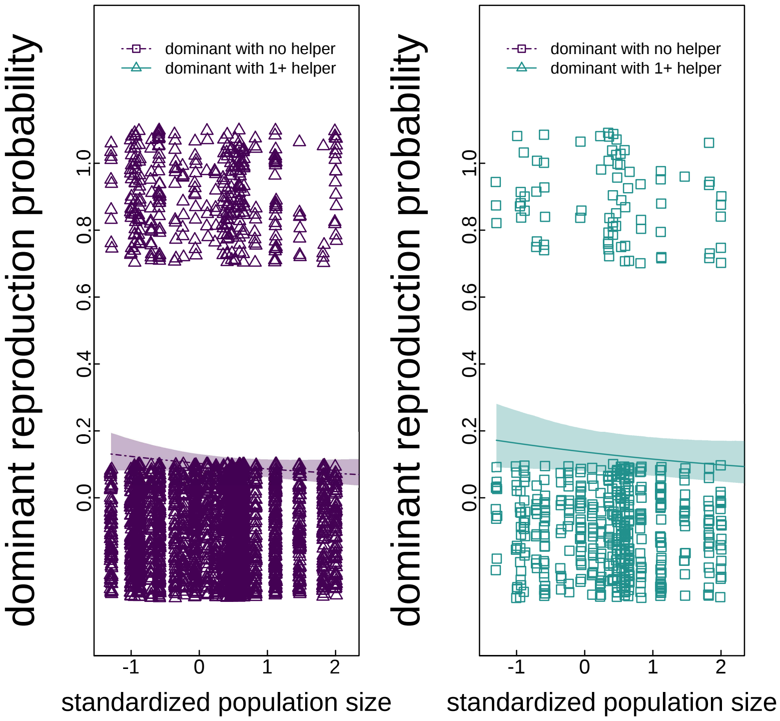

Figure A9. Raw data and predictions of the reproduction probability of helpers as a function of age. Solid lines represent the maximum a posteriori estimate of the mean reproduction probability. The shaded regions represent 89% prediction interval in the population at each age. The standardized population size was fixed at 0. Please note that we used the function jitter (from base R Core Team, 2020) to add a little random vertical noise to the data to see individual data points more clearly. All observed values equal 0 or 1 with respect to the y-axis, even if they appear larger or smaller.

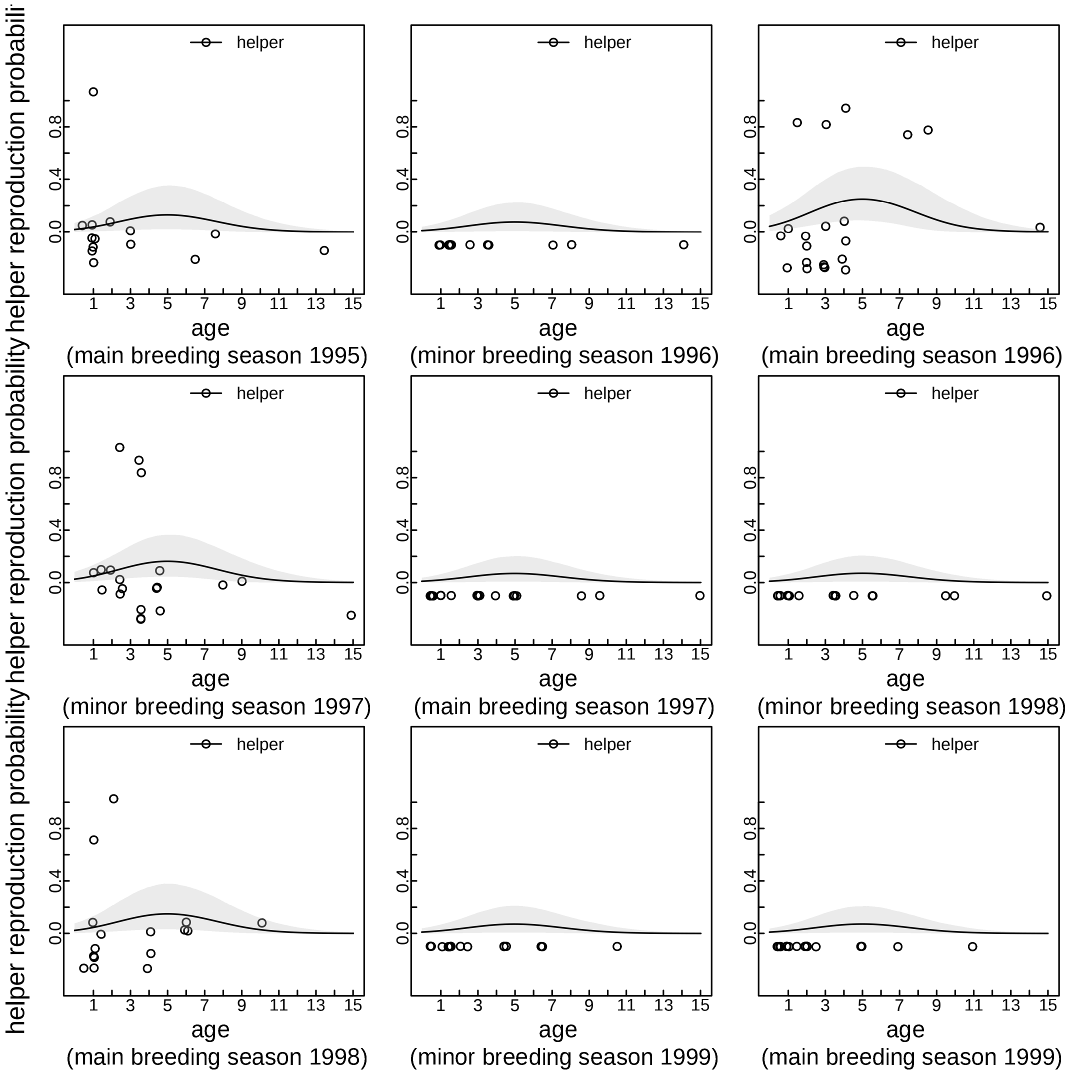

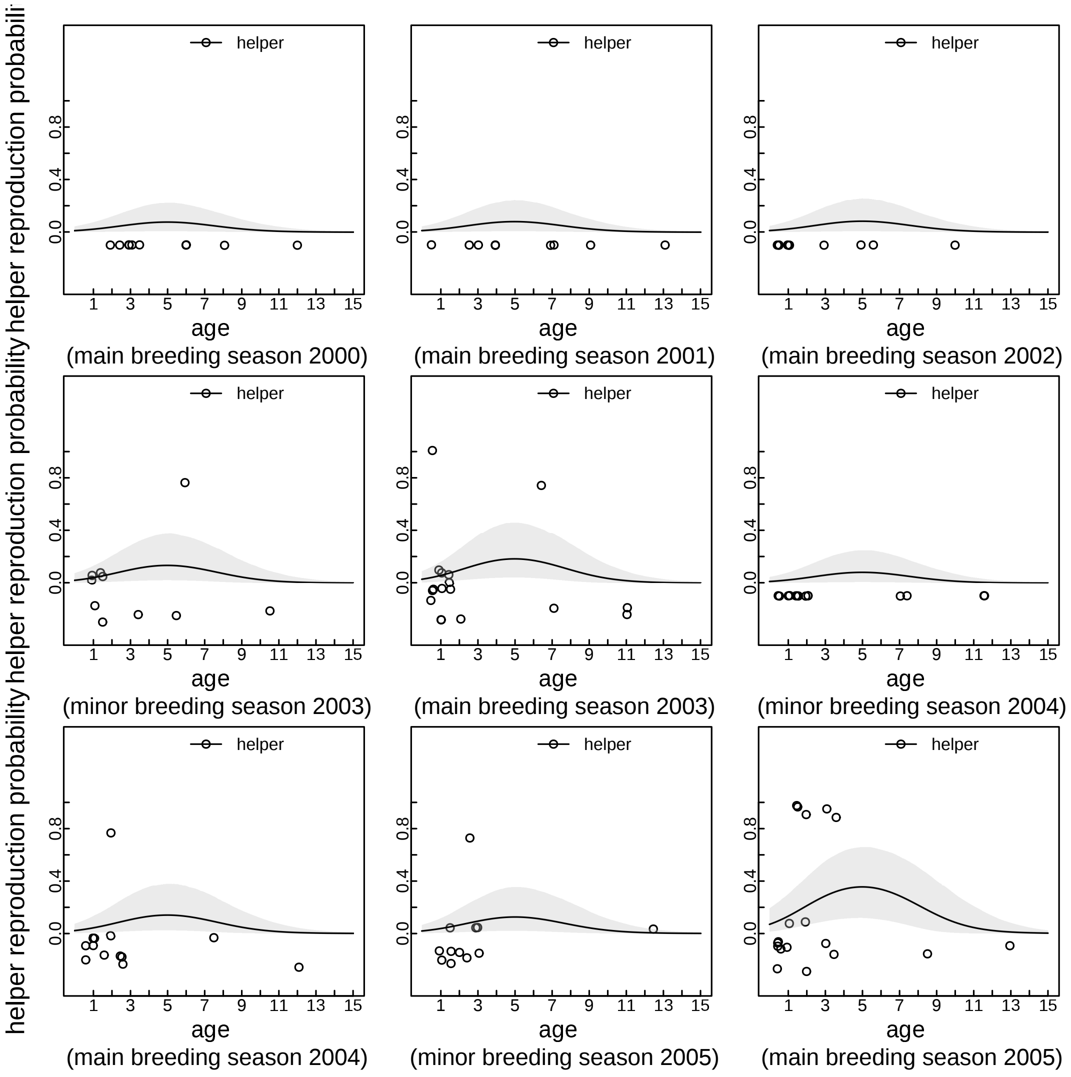
helper reproduction probability
helper
0.0
0.4
0.8
1 3 5 7 9 11 13 15
age
(main breeding season 2000)
(main breeding season 2001)
(main breeding season 2002)
(minor breeding season 2003)
(main breeding season 2003)
(minor breeding season 2004)
(main breeding season 2004)
(minor breeding season 2005)
(main breeding season 2005)

(a)

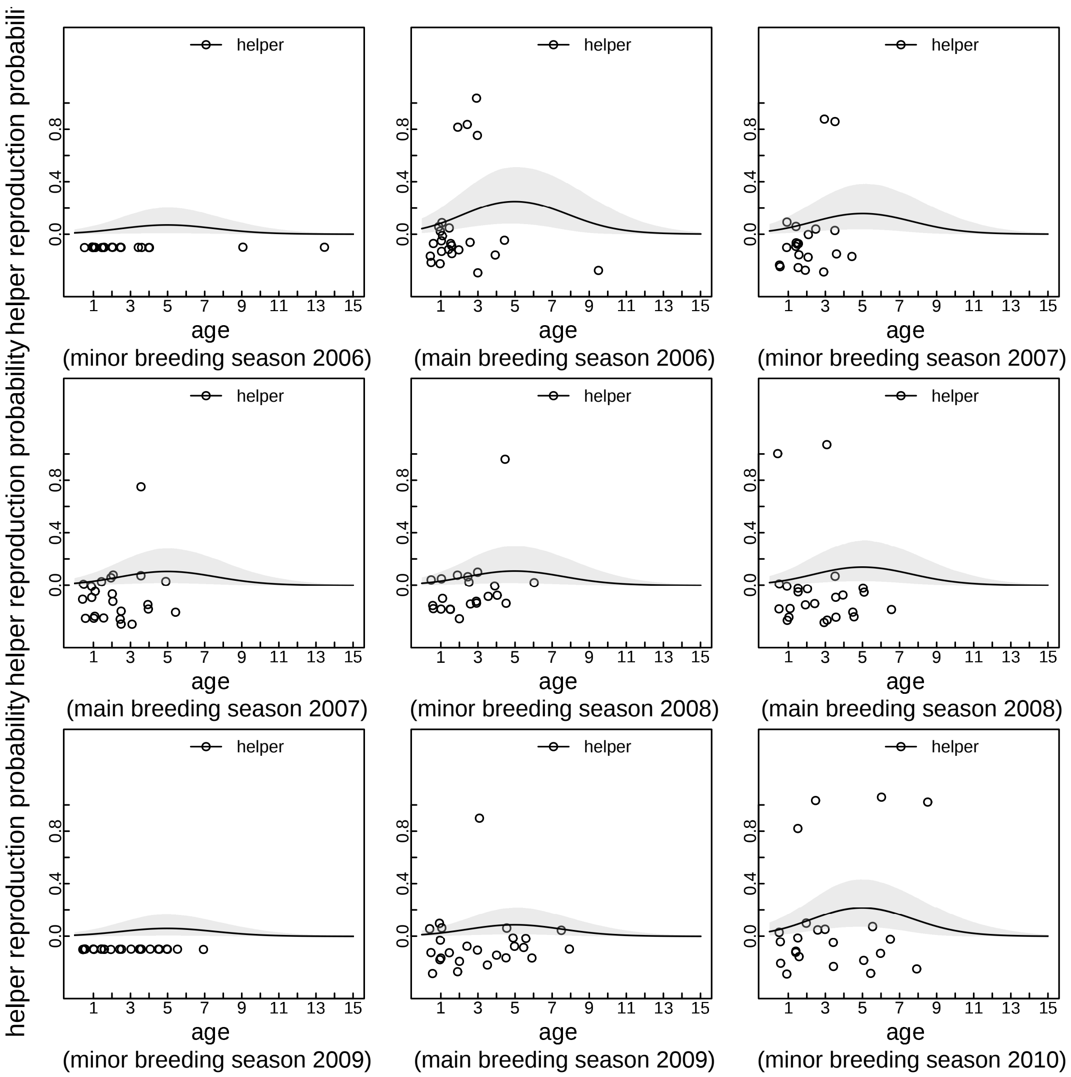
helper reproduction probability
helper
0.0
0.4
0.8
1
3
5
7
9
11
13
15
age
(minor breeding season 2006)
(main breeding season 2006)
(minor breeding season 2007)
(main breeding season 2007)
(minor breeding season 2008)
(main breeding season 2008)
(minor breeding season 2009)
(main breeding season 2009)
(minor breeding season 2010)

(b)

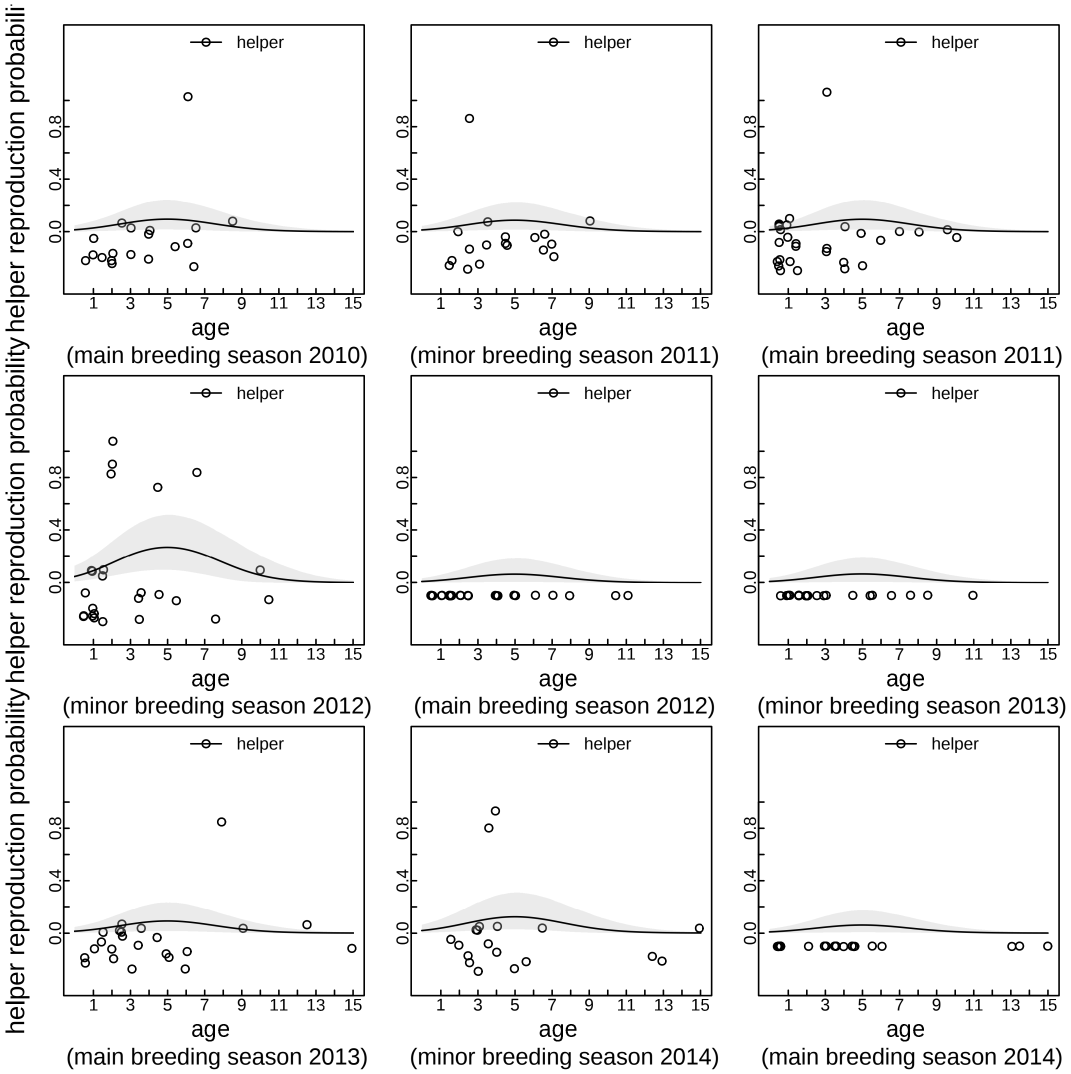
helper
0.0
0.4
0.8
1 3 5 7 9 11 13 15
age
(main breeding season 2010)
(minor breeding season 2011)
(main breeding season 2011)
(minor breeding season 2012)
(main breeding season 2012)
(minor breeding season 2013)
(main breeding season 2013)
(minor breeding season 2014)
(main breeding season 2014)
helper reproduction probability
helper reproduction probability
helper reproduction probabili

(c)

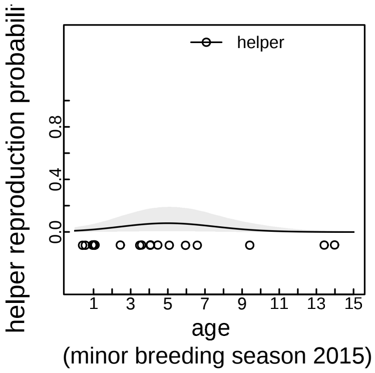

helper
helper reproduction probabili
0.0
0.4
0.8
1
3
5
7
9
11
13
15
age
(minor breeding season 2015)

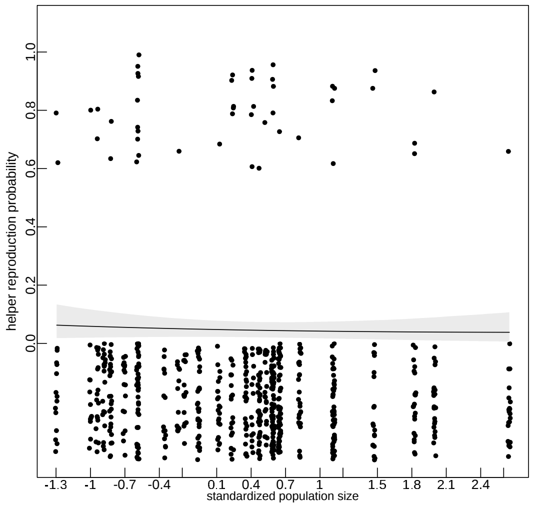

Figure A10. Raw data and predictions of the reproduction probability of helpers as a function of the standardized population size. Solid lines represent the maximum a posteriori estimate of the mean reproduction probability in different seasons. The age was fixed at two. Please note that we used the function jitter (from base R Core Team, 2020) to add a little random vertical noise to the data to see individual data points more clearly. All observed values equal 0 or 1 with respect to the y-axis, even if they appear larger or smaller.

Figure A11. Raw data and predictions of the probability of an offspring becoming a helper as a function of the age of its mother. Solid lines represent the maximum a posteriori estimate of the mean probability. The shaded regions represent 89% prediction interval in the population at each age. In the plots representing the probability as a function of age the standardized population size was fixed at 0. Multiple reasons might explain why the probability of becoming a helper depends on the mother's age. One possibility is that dominant females are more likely to successfully reproduce as time passes (just because the number of reproductive attempts increases). If helpers are the offspring produced in the previous season then it would follow that older dominant females have higher chances of having a helper in the territory than younger dominants. Additionally, if the benefits of receiving help increase as a mother ages, then older mothers should recruit and retain helpers (Hammers *et al.*, 2019a,b). However, it is hard to speculate because 5% of individuals that disperse become helpers in a non-natal territory where they are not related to the dominant female (Groenewoud *et al.*, 2018). Another possibility for non-related helpers is that older dominant females had a longer bond with the dominant male. Possibly well-established couples can hold larger or higher quality territories, which could attract helpers. To make this claim it is necessary to look also at the breeding male and at territory quality. Please note that we used the function jitter (from base R Core Team, 2020) to add a little random vertical noise to the data to see individual data points more clearly. All observed values equal 0 or 1 with respect to the y-axis, even if they appear larger or smaller.

(a)

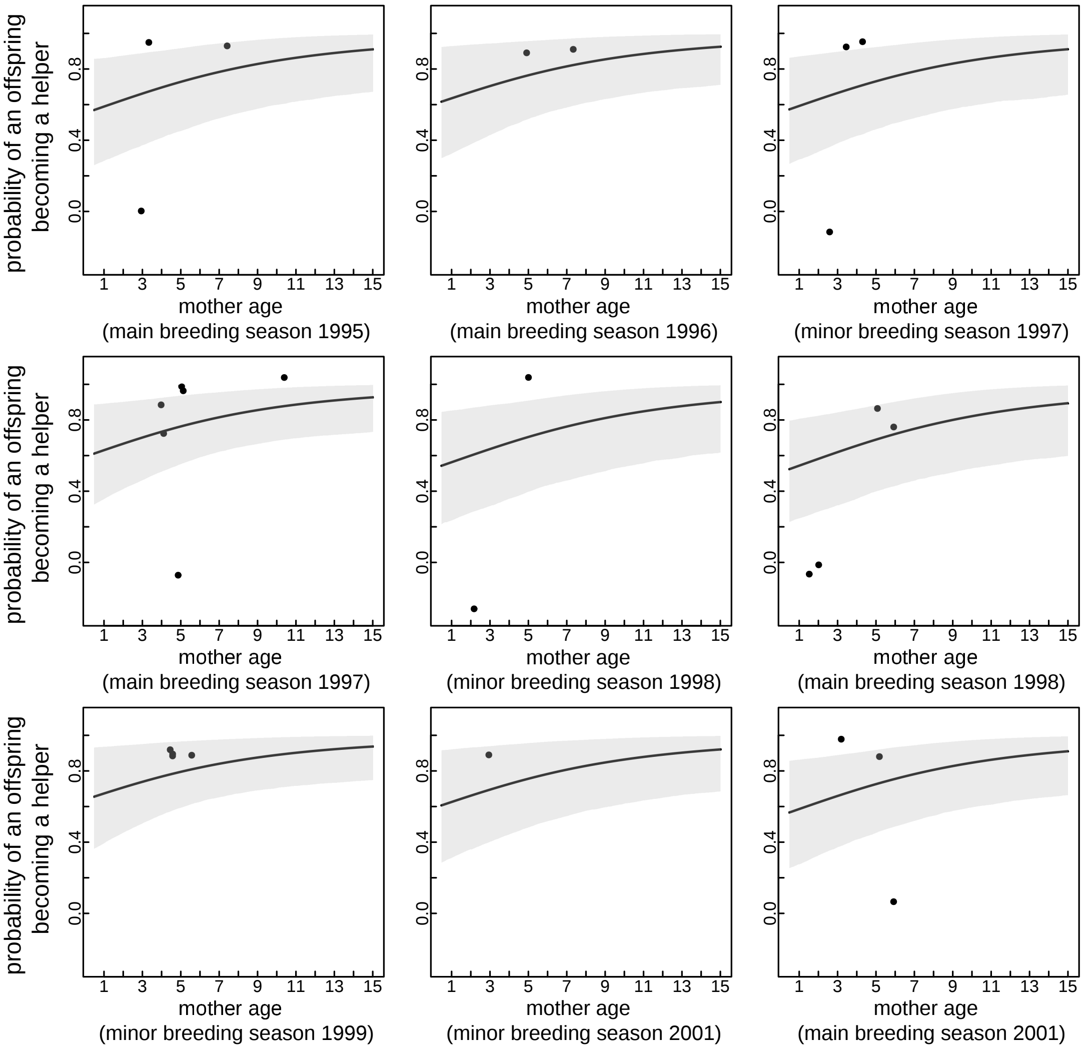

(b)

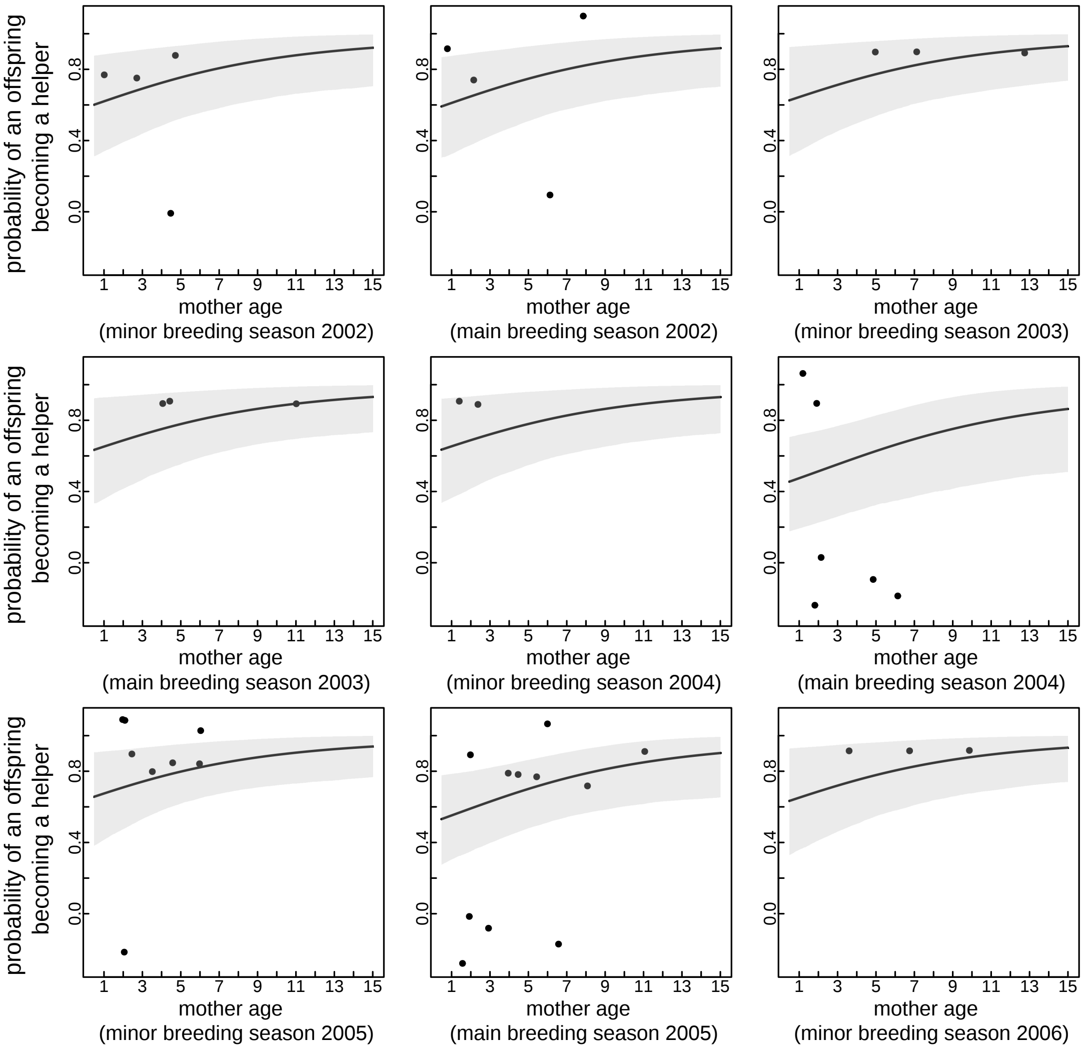
probability of an offspring becoming a helper
0.0
0.4
0.8
1
3
5
7
9
11
13
15
mother age
(minor breeding season 2002)
(main breeding season 2002)
(minor breeding season 2003)
(main breeding season 2003)
(minor breeding season 2004)
(main breeding season 2004)
(minor breeding season 2005)
(main breeding season 2005)
(minor breeding season 2006)

(c)

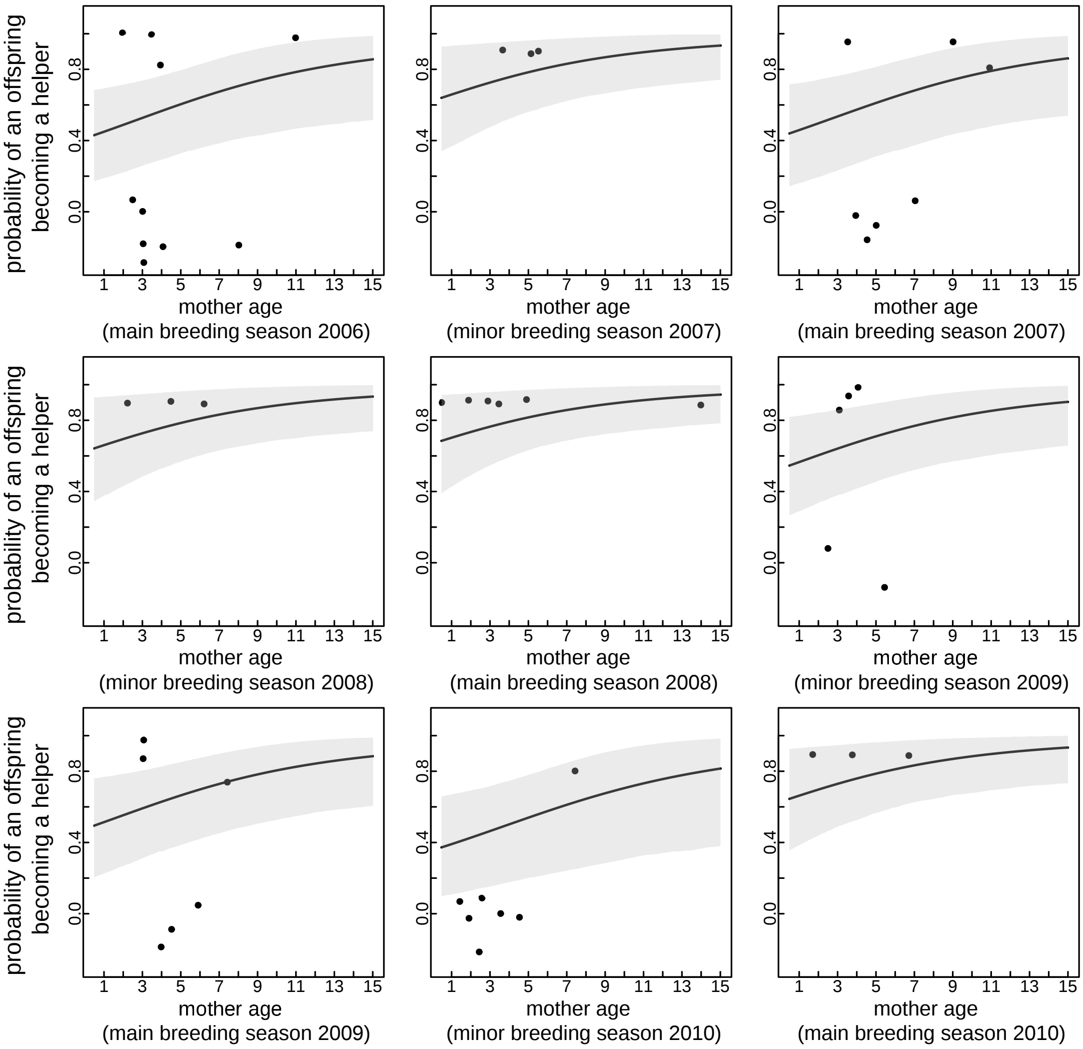
probability of an offspring becoming a helper
0.0
0.4
0.8
1
3
5
7
9
11
13
15
mother age
(main breeding season 2006)
(minor breeding season 2007)
(main breeding season 2007)
(minor breeding season 2008)
(main breeding season 2008)
(minor breeding season 2009)
(main breeding season 2009)
(minor breeding season 2010)
(main breeding season 2010)

(d)

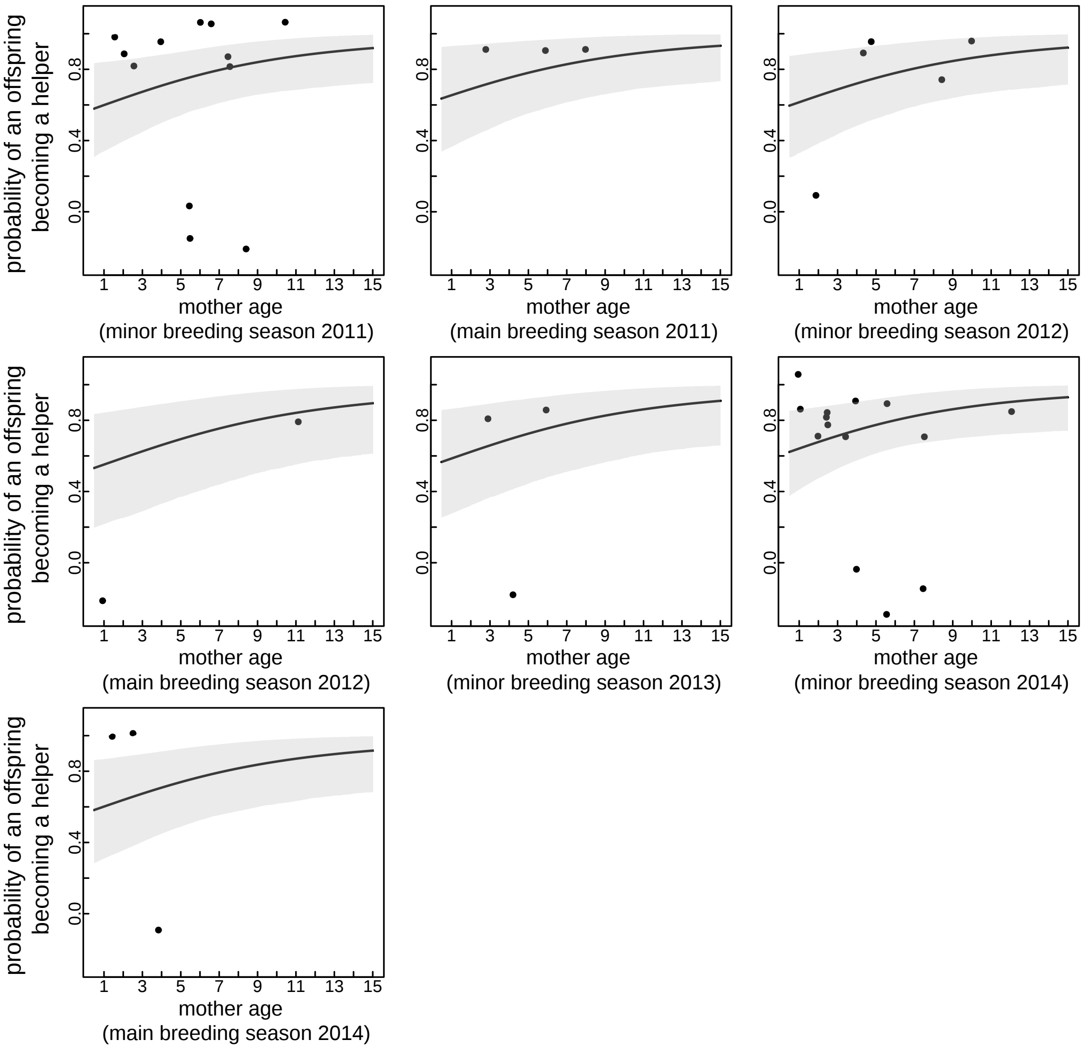

probability of an offspring becoming a helper
0.0
0.4
0.8
1 3 5 7 9 11 13 15
mother age
(minor breeding season 2011)
(main breeding season 2011)
(minor breeding season 2012)
(main breeding season 2012)
(minor breeding season 2013)
(minor breeding season 2014)
(main breeding season 2014)

Figure A12. Raw data and predictions of the probability that a helper is present in a territory. Solid lines represent the maximum a posteriori estimate of the mean probability. The shaded regions represent 89% prediction interval in the population at each age. Please note that we used the function jitter (from base R Core Team, 2020) to add a little random vertical noise to the data to see individual data points more clearly. All observed values equal 0 or 1 with respect to the y-axis, even if they appear larger or smaller.

(a) In the plot representing the probability as a function of age, the ratio between the number of subordinates helpers and non-helpers over the number of dominants was fixed at 0.

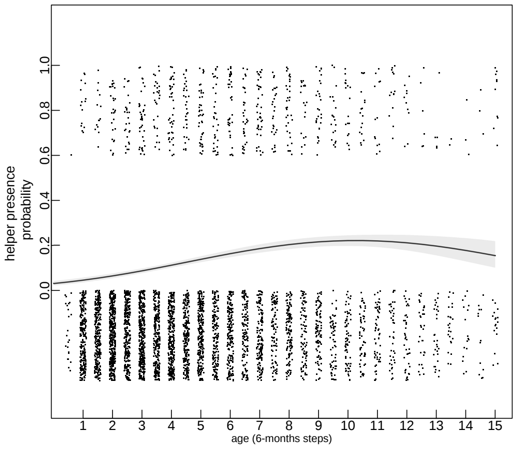

(b) In the plot representing the probability as a function of the ratio between the number of subordinates helpers and non-helpers over the number of dominants, age was fixed at 2.

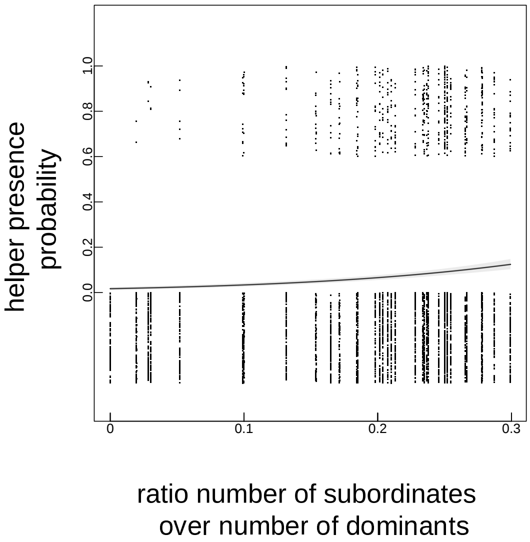

A9 POSTERIOR PREDICTIVE CHECKS: DENSITY PLOTS

We assessed the fit of the statistical models checking the posterior distribution for all the parameter estimates (McElreath, 2020). The following graphs represent the density of the posterior prediction for all the parameters to represent the uncertainty around their mean estimates. These plots refer to the analysis of the observed data (see Table A3).

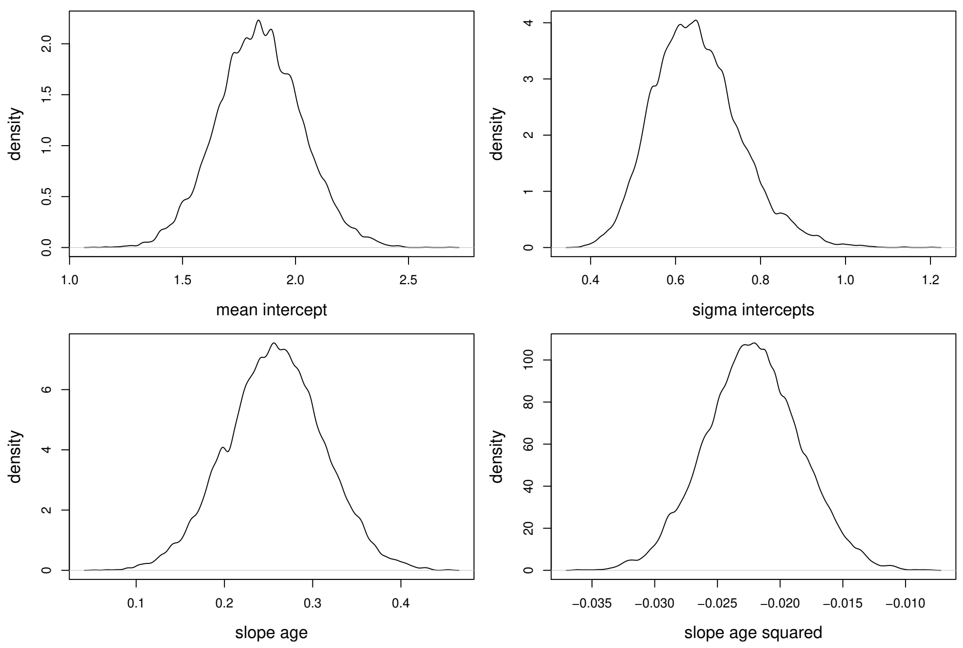

Figure A13. Quadratic approximate posterior distribution of the parameter estimates of the survival probability of dominants. Here the intercept is represented by its mean and standard deviation.

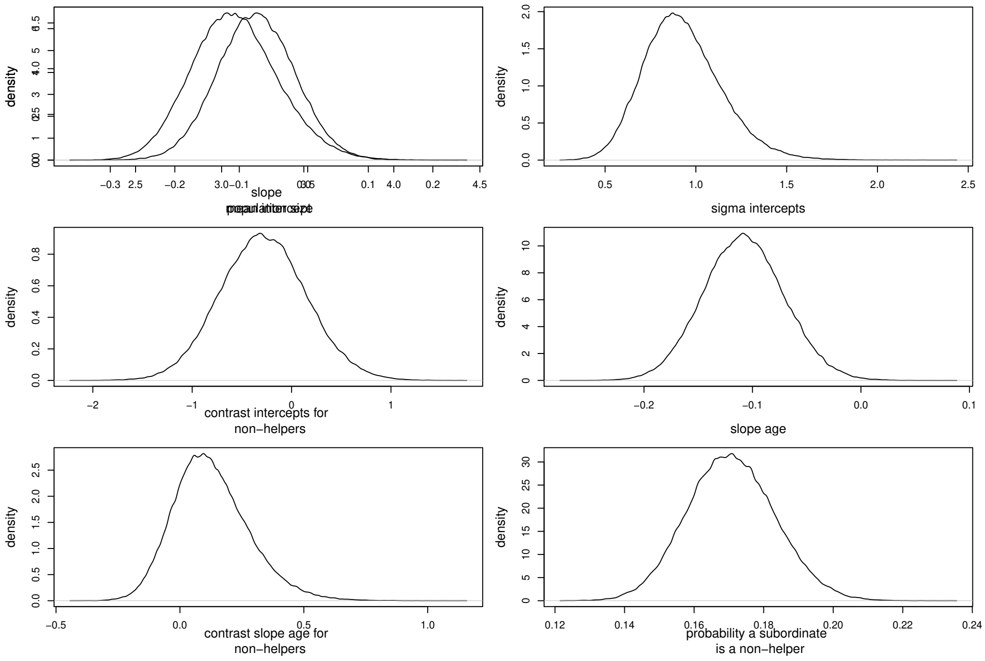

Figure A14. Quadratic approximate posterior distribution of the parameter estimates of the survival probability of helpers and non-helpers. Here the intercept is represented by its mean and standard deviation.

Figure A15. Quadratic approximate posterior distribution of the varying intercepts of the survival probability

(a)

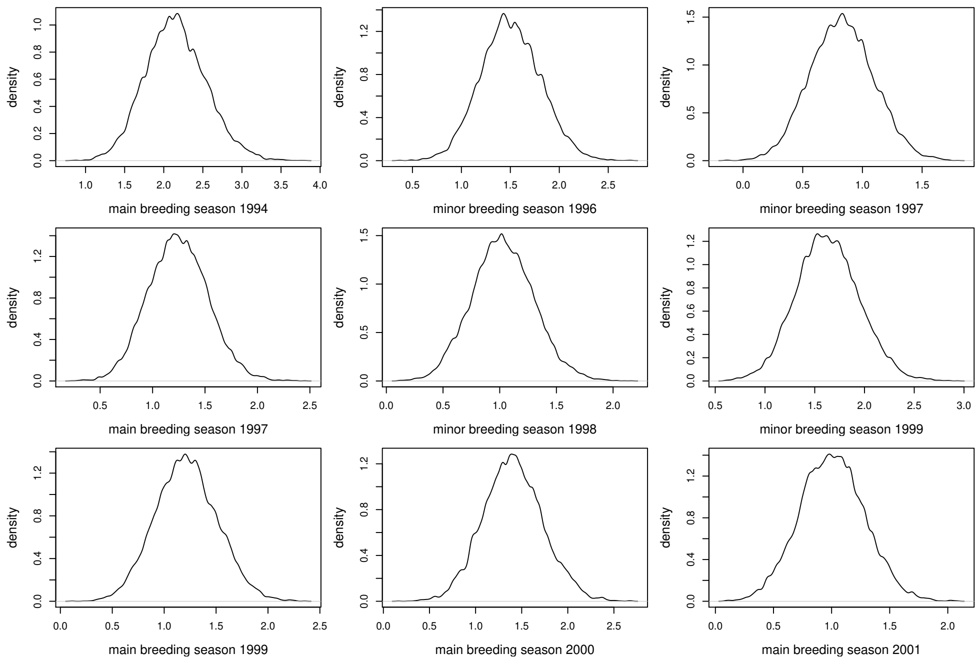

(b)

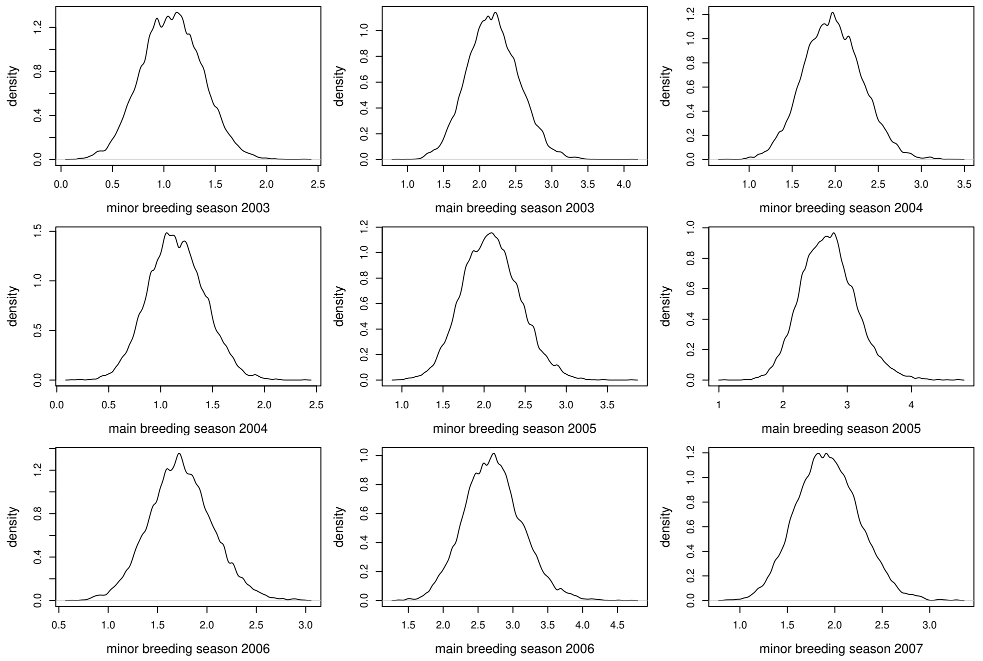
density
minor breeding season 2003
main breeding season 2003
minor breeding season 2004
main breeding season 2004
minor breeding season 2005
main breeding season 2005
minor breeding season 2006
main breeding season 2006
minor breeding season 2007

(c)

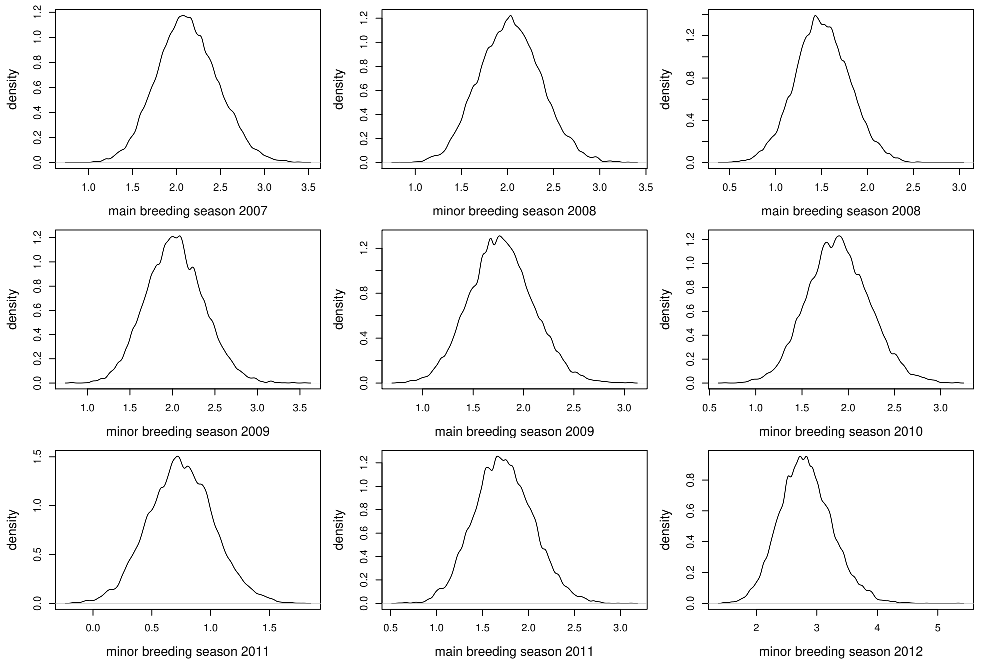
density
main breeding season 2007
minor breeding season 2008
main breeding season 2008
minor breeding season 2009
main breeding season 2009
minor breeding season 2010
minor breeding season 2011
main breeding season 2011
minor breeding season 2012

(d)

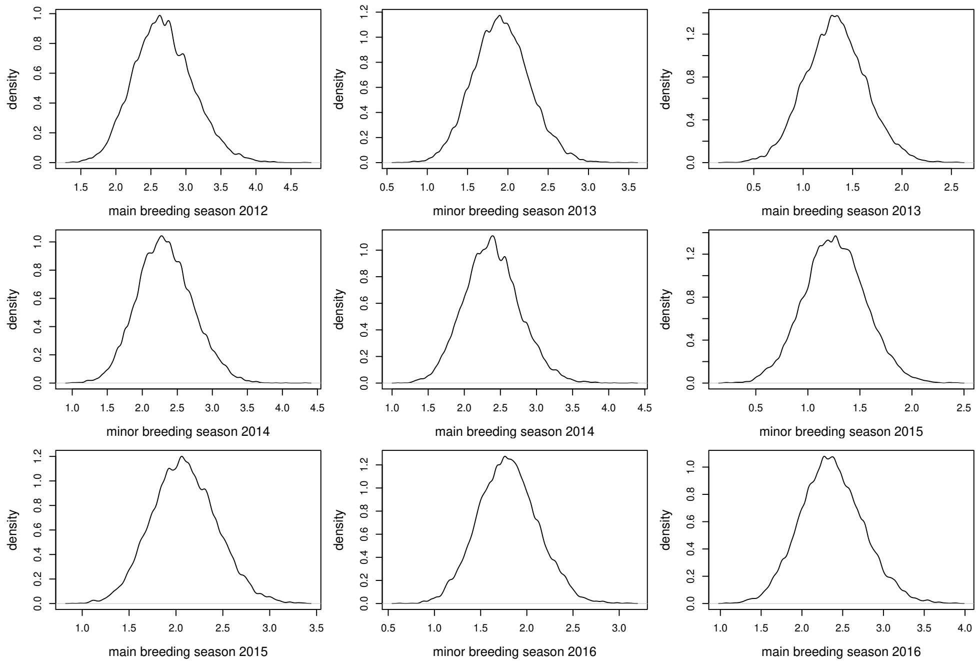
density
main breeding season 2012
minor breeding season 2013
main breeding season 2013
minor breeding season 2014
main breeding season 2014
minor breeding season 2015
main breeding season 2015
minor breeding season 2016
main breeding season 2016

(e)

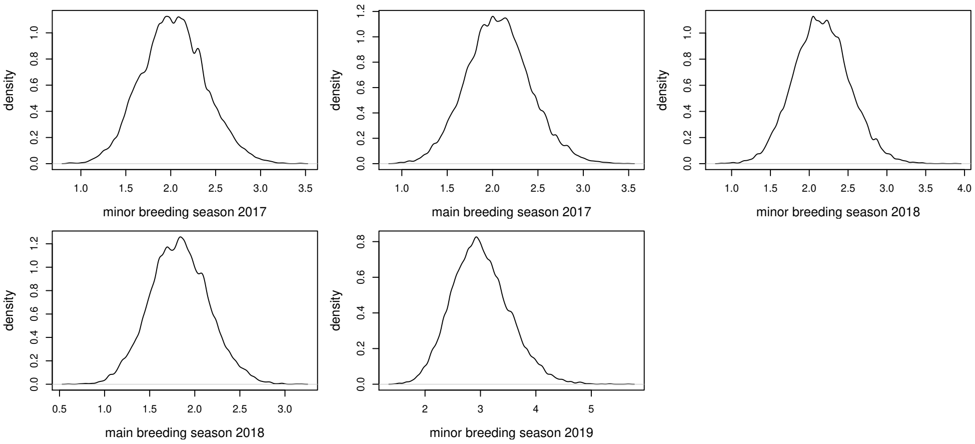
density
minor breeding season 2017
density
main breeding season 2017
density
minor breeding season 2018
density
main breeding season 2018
density
minor breeding season 2019

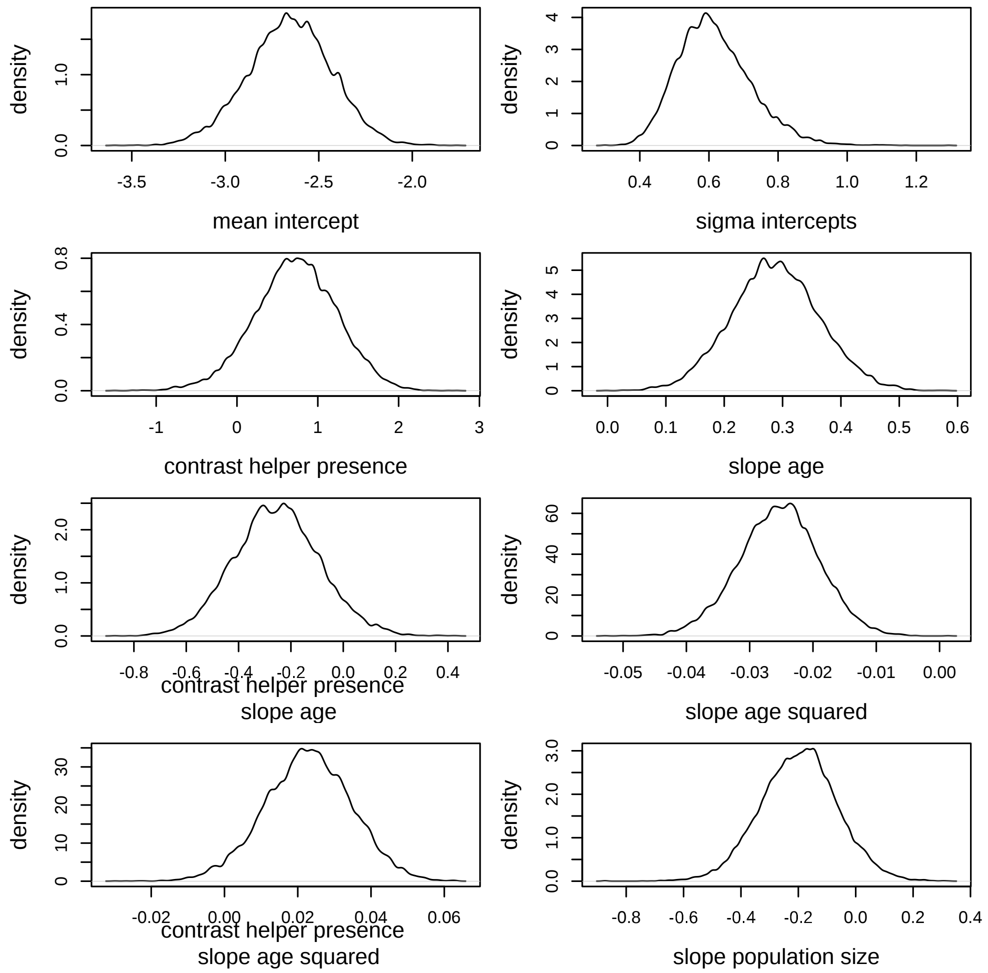

Figure A16. Quadratic approximate posterior distribution of the parameter estimates of the reproduction probability of dominants. Here the intercept is represented by its mean and standard deviation.

Figure A17. Quadratic approximate posterior distribution of the varying intercepts of the reproduction probability of dominants.

(a)

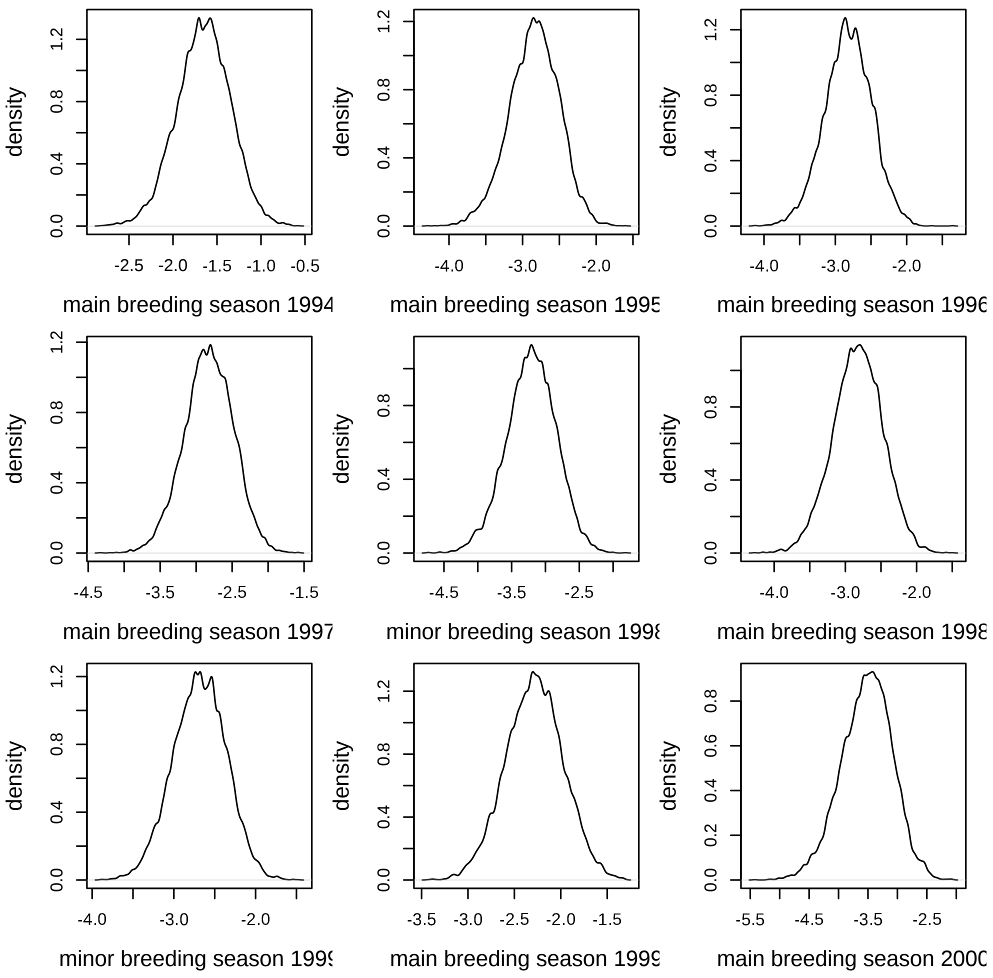

(b)

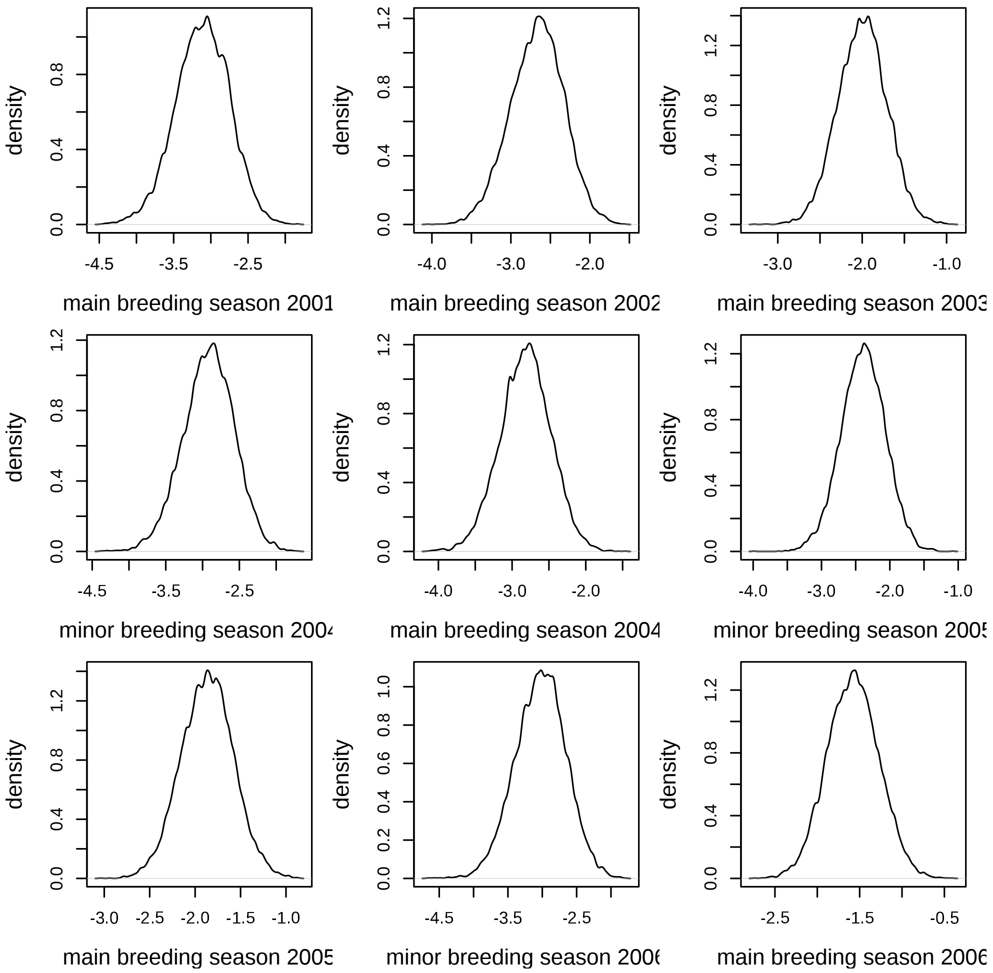
density
main breeding season 2001
main breeding season 2002
main breeding season 2003
minor breeding season 2004
main breeding season 2004
minor breeding season 2005
main breeding season 2005
minor breeding season 2006
main breeding season 2006

(c)

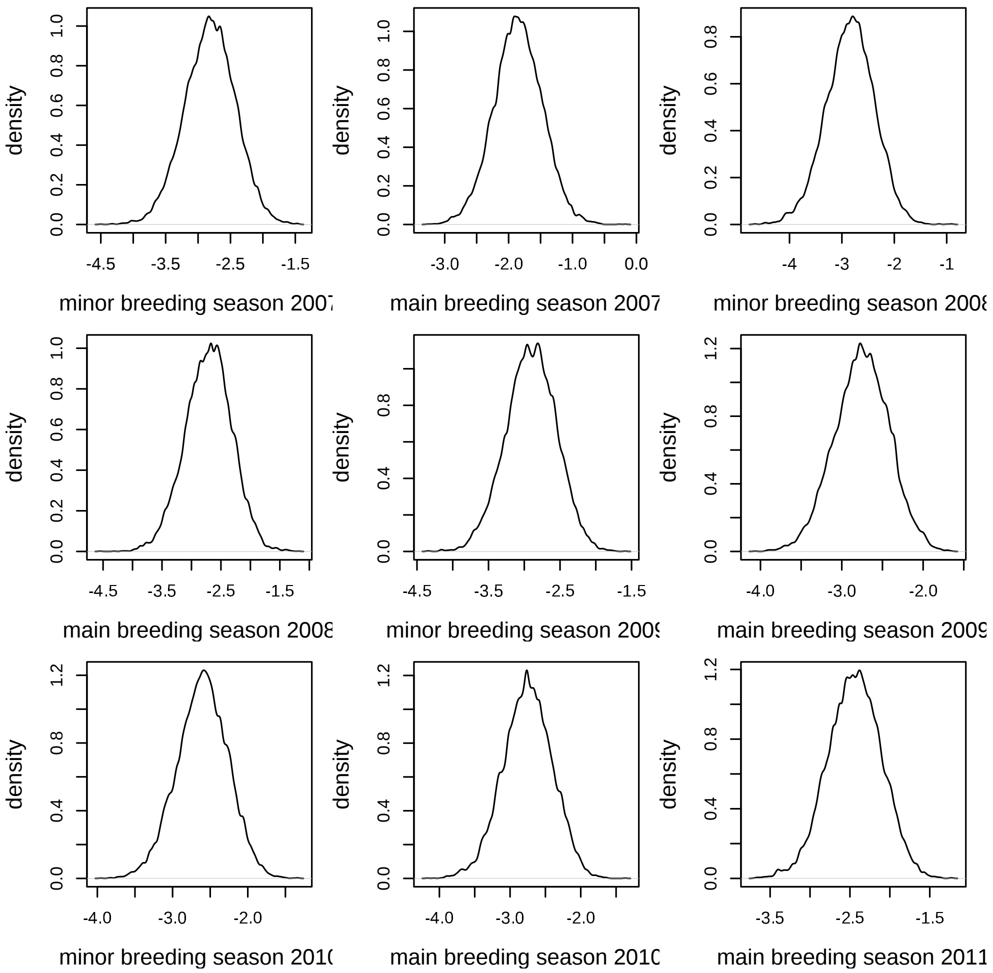

density
minor breeding season 2007
main breeding season 2007
minor breeding season 2008
main breeding season 2008
minor breeding season 2009
main breeding season 2009
minor breeding season 2010
main breeding season 2010
main breeding season 2011

(d)

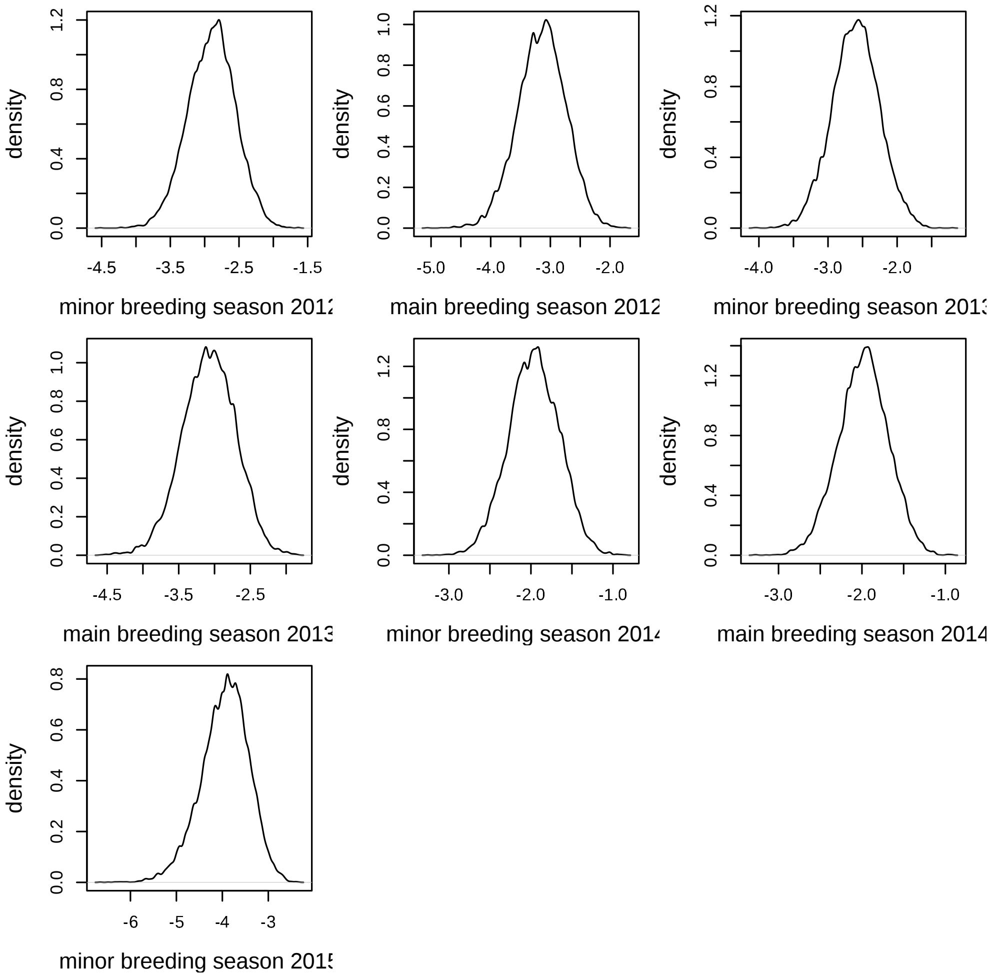
density
minor breeding season 201
main breeding season 201
minor breeding season 201
main breeding season 201
minor breeding season 201
main breeding season 201
minor breeding season 201

Figure A18. Quadratic approximate posterior distribution of the parameter estimates of the reproduction probability of helpers. Here the intercept is represented by its mean and standard deviation.

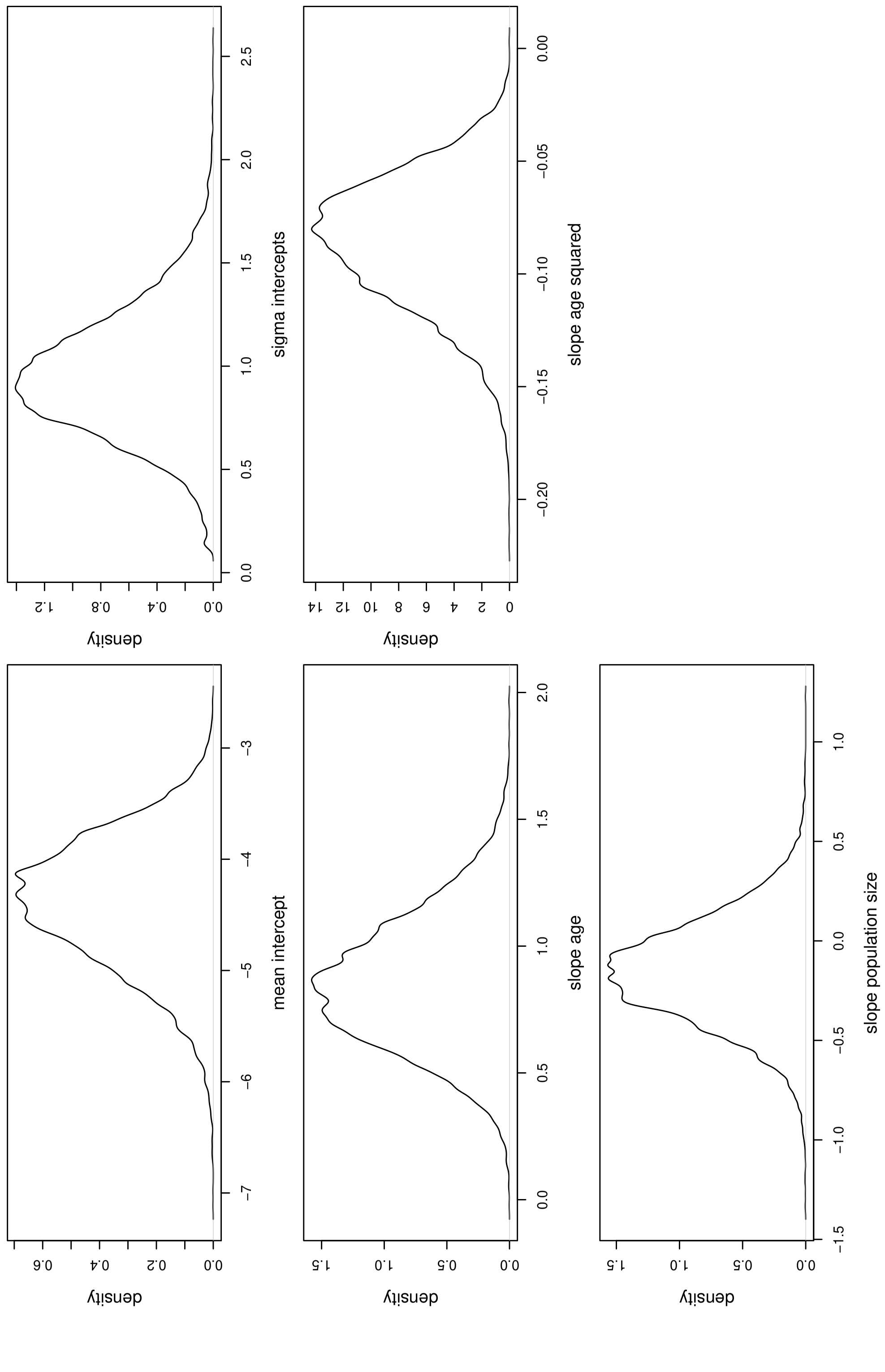

Figure A19. Quadratic approximate posterior distribution of the varying intercepts of the reproduction probability of helpers.

(a)

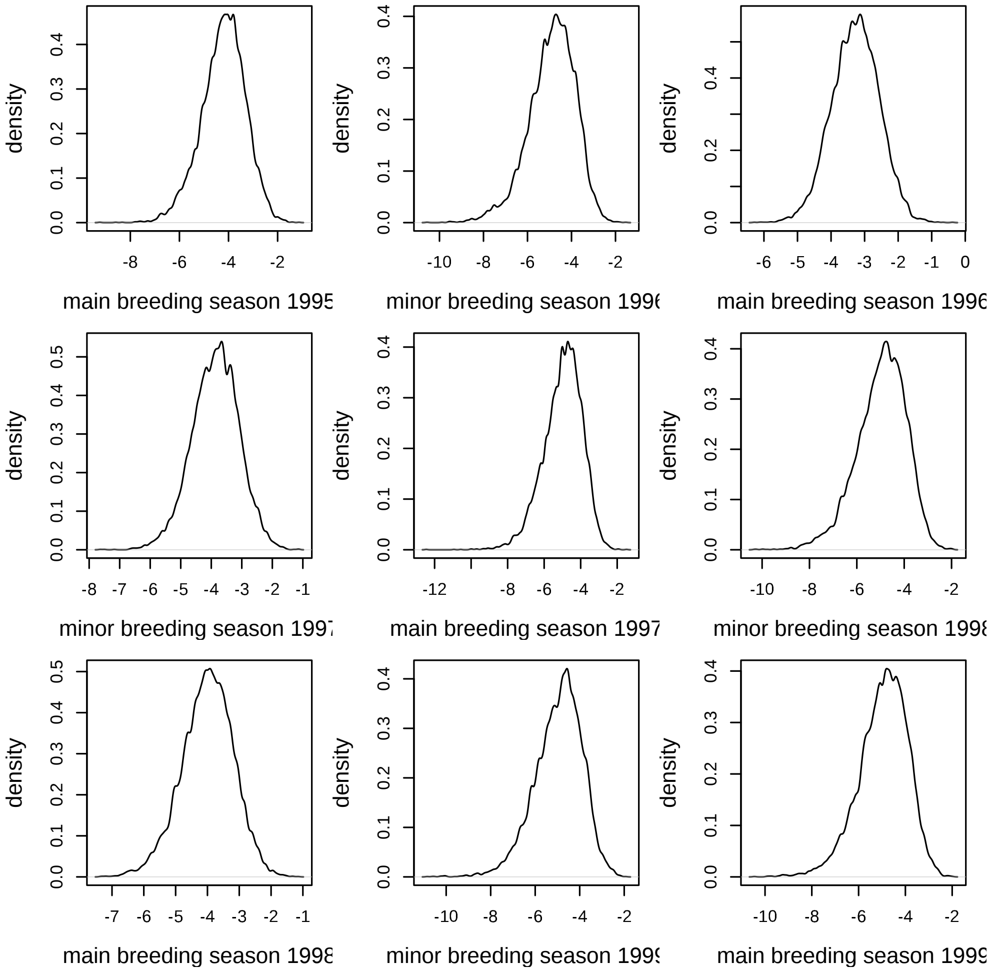

(b)

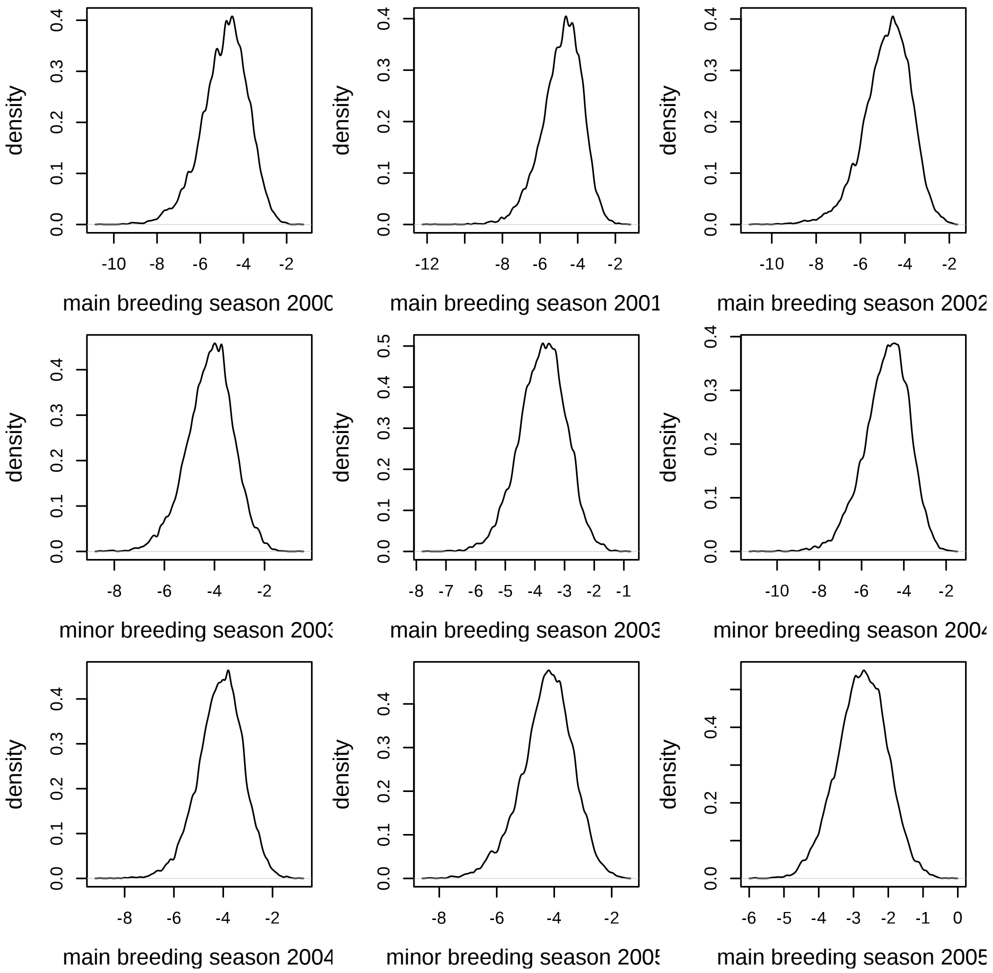
density
main breeding season 2000
main breeding season 2001
main breeding season 2002
minor breeding season 2003
main breeding season 2003
minor breeding season 2004
main breeding season 2004
minor breeding season 2005
main breeding season 2005

(c)

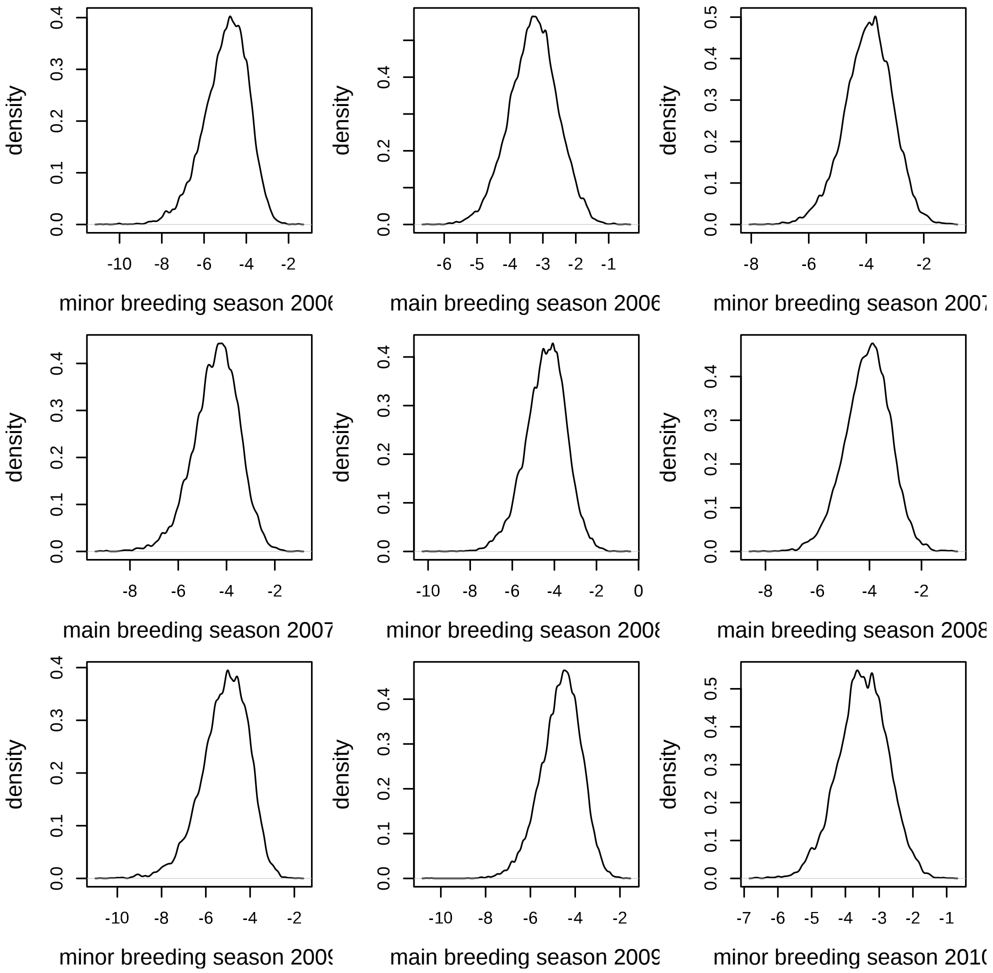
density
minor breeding season 200
main breeding season 200
minor breeding season 200
main breeding season 200
minor breeding season 200
main breeding season 200
minor breeding season 200
main breeding season 200
minor breeding season 201

(d)

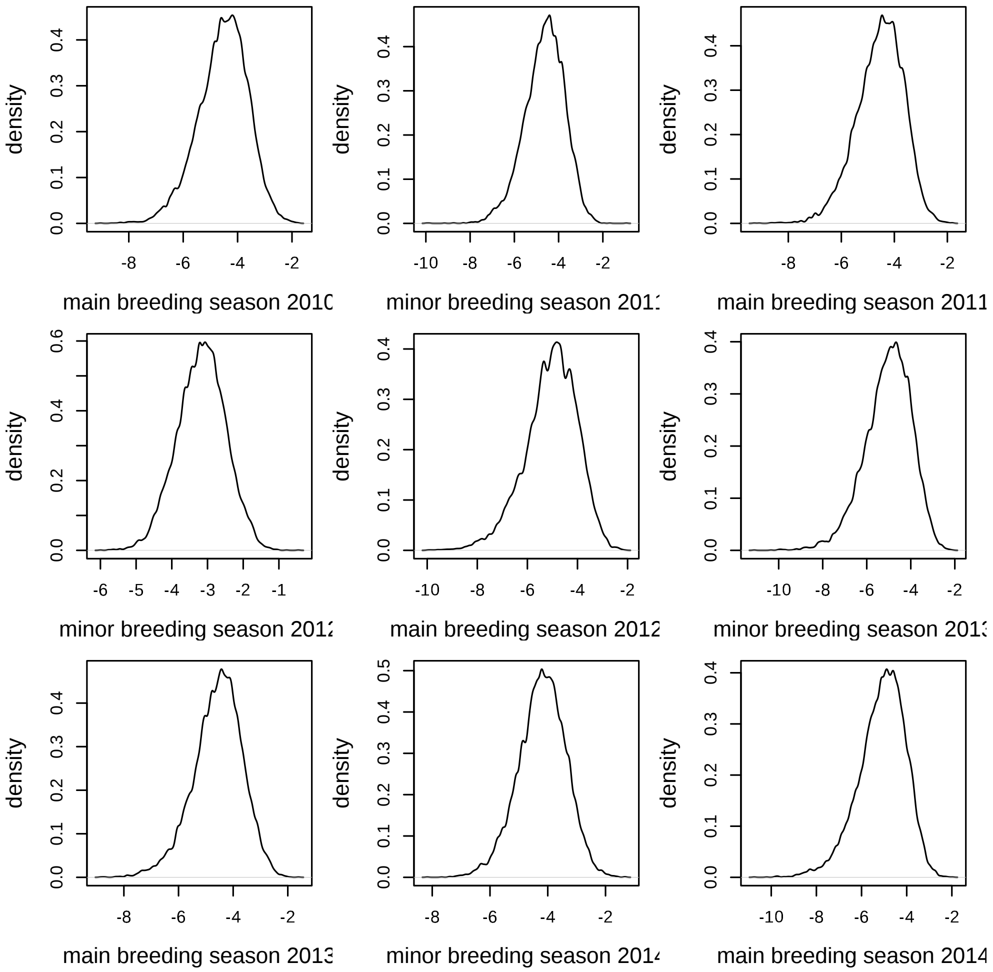

density
main breeding season 2010
minor breeding season 2011
main breeding season 2011
minor breeding season 2012
main breeding season 2012
minor breeding season 2013
main breeding season 2013
minor breeding season 2014
main breeding season 2014

(e)

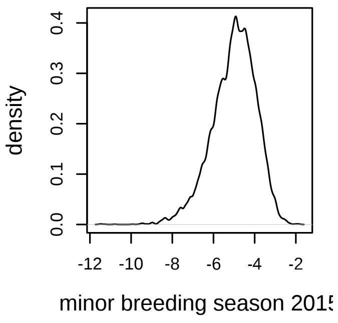

Figure A20. Quadratic approximate posterior distribution of the parameter estimates of the probability of an offspring becoming a helper. Here the intercept is represented by its mean and standard deviation

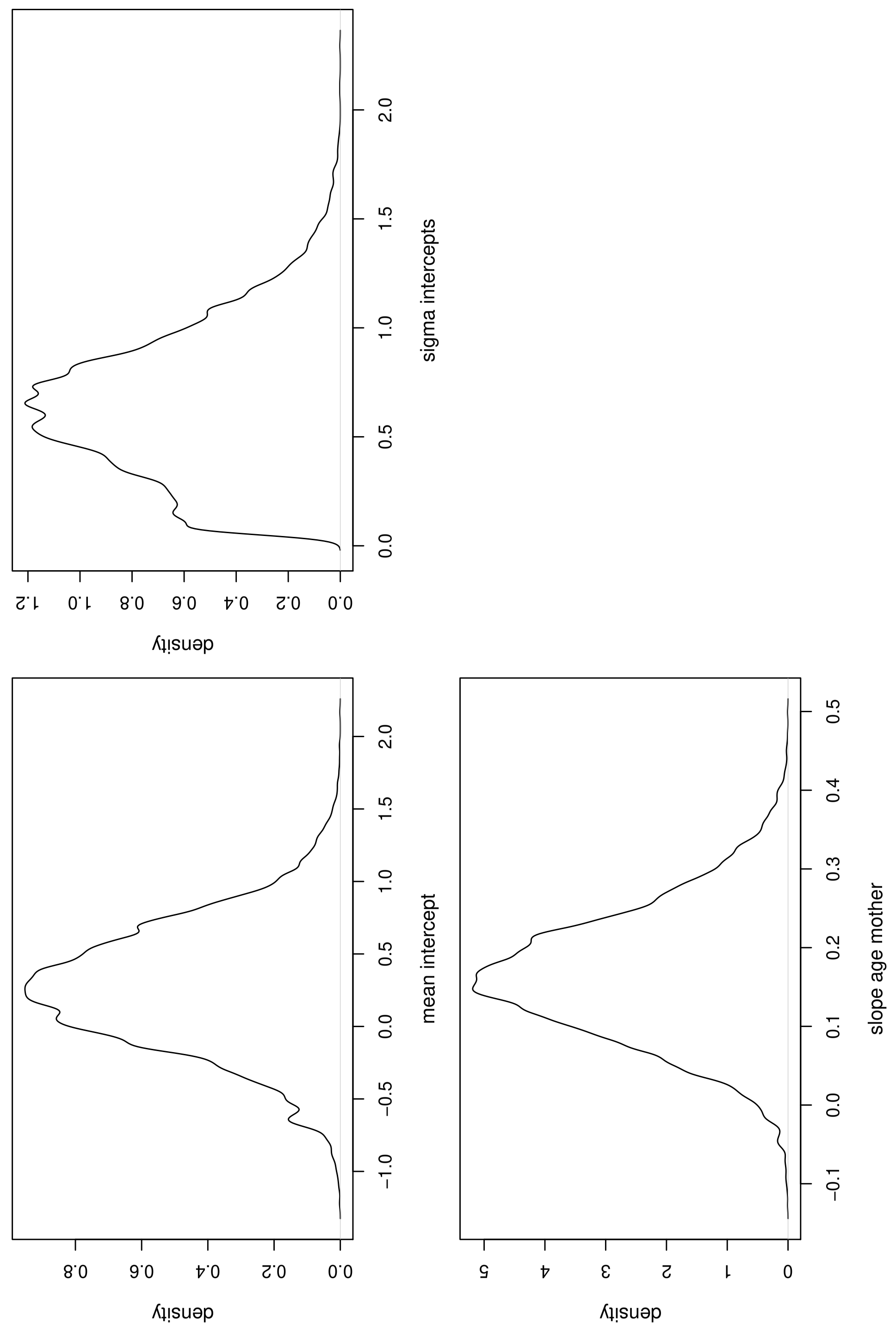

Figure A21. Quadratic approximate posterior distribution of the varying intercepts of the probability of an offspring becoming a helper.

(a)

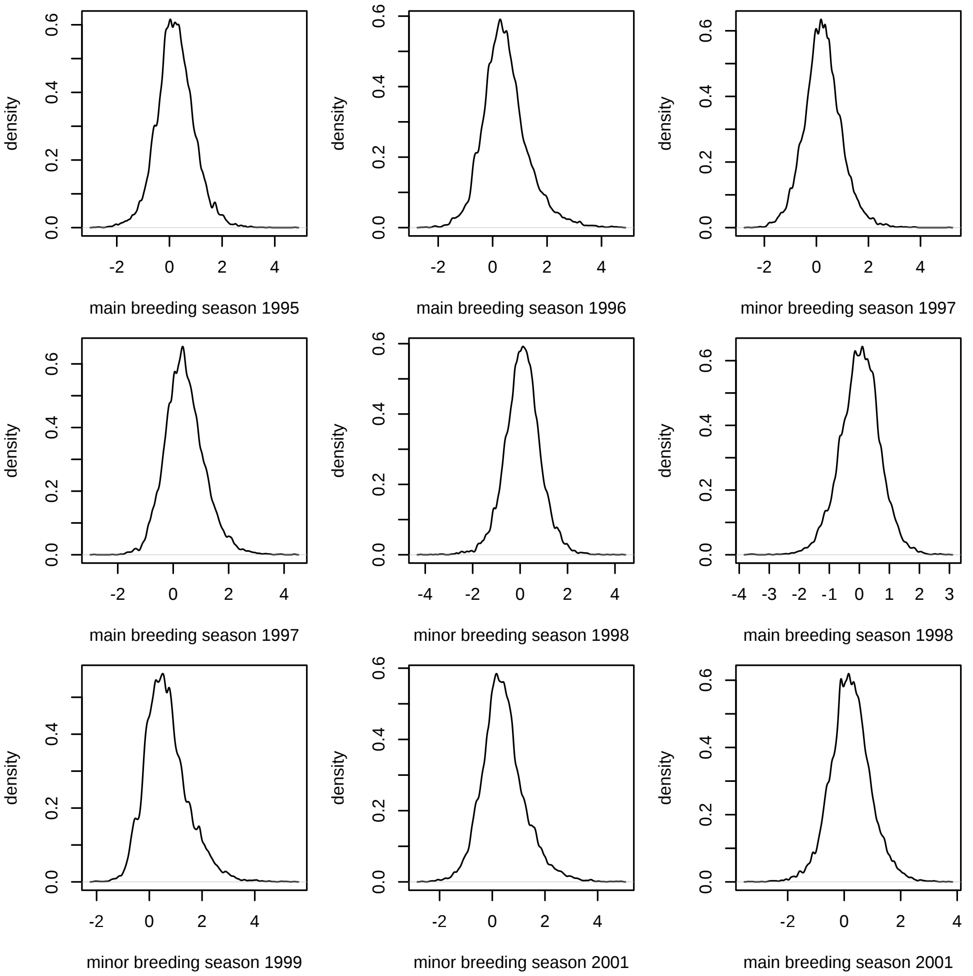

(b)

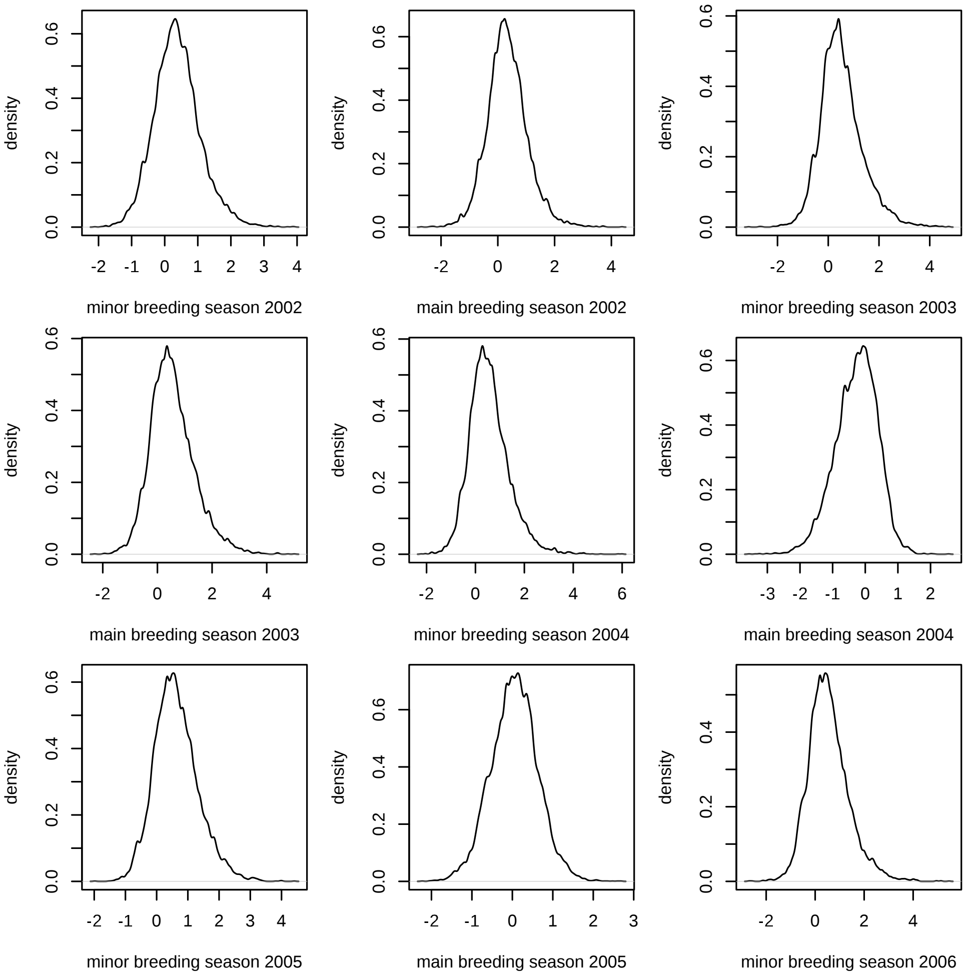
density
minor breeding season 2002
main breeding season 2002
minor breeding season 2003
main breeding season 2003
minor breeding season 2004
main breeding season 2004
minor breeding season 2005
main breeding season 2005
minor breeding season 2006

(c)

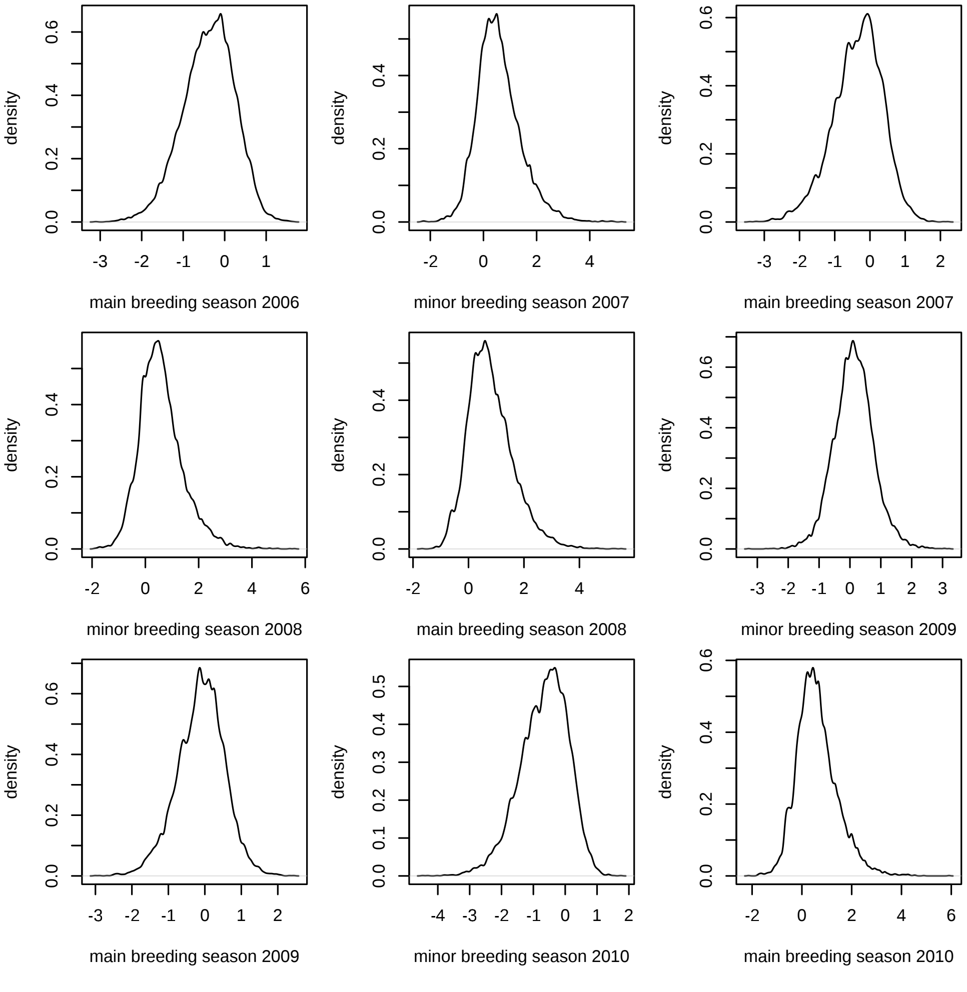
density
main breeding season 2006
minor breeding season 2007
main breeding season 2007
minor breeding season 2008
main breeding season 2008
minor breeding season 2009
main breeding season 2009
minor breeding season 2010
main breeding season 2010

(d)

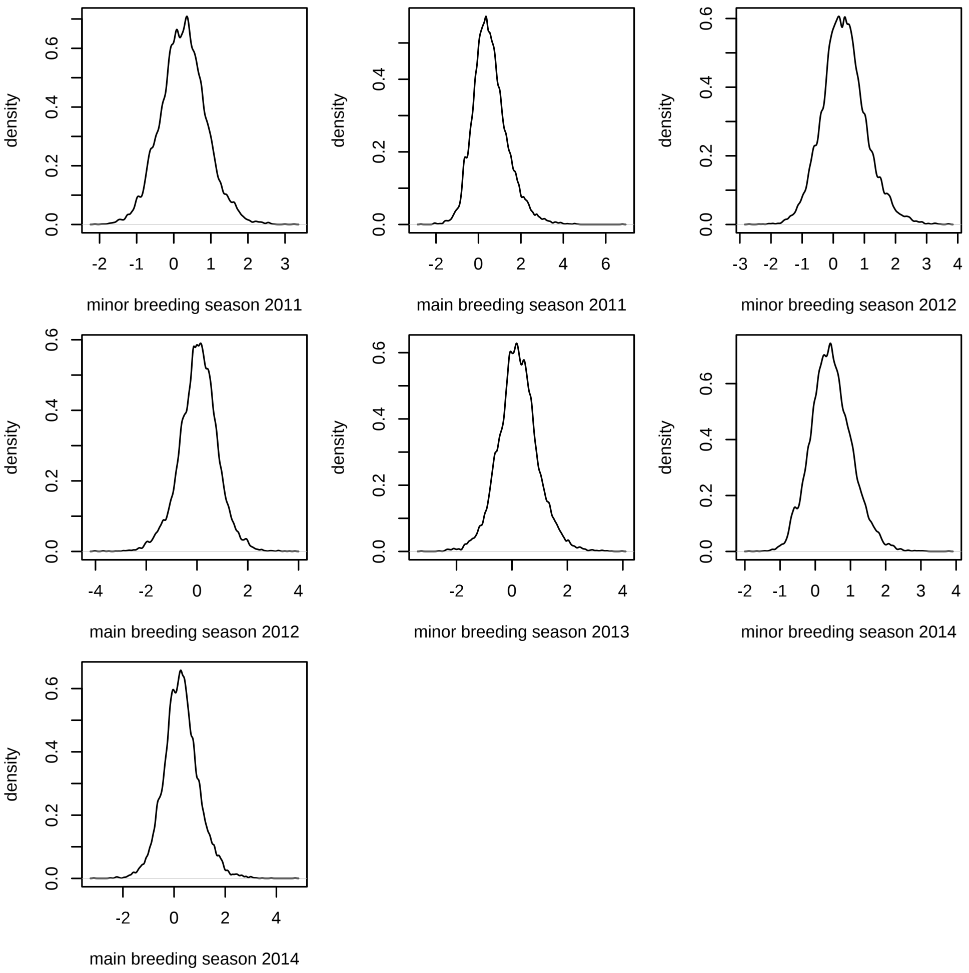
density
minor breeding season 2011
main breeding season 2011
minor breeding season 2012
main breeding season 2012
minor breeding season 2013
minor breeding season 2014
main breeding season 2014

A10 LIFE TABLE RESPONSE EXPERIMENT

The goal of a random life table response experiment (LTRE) is to decompose the variance in the $\hat{y}$ into contributions from the variability of each vital rate (Caswell, 2001). The word random implies that the process in which the vital rates are perturbed during the computation is randomized. The calculation of the $Var(\hat{y})$ requires solving (or finding an approximation of) the partial derivative of $\hat{y}$ both over the mean and the variance of a specific time-varying vital rate. This is because the contribution of a specific vital rate depends on how much the vital rate itself varied and how much $\hat{y}$ is sensitive to that variation (Grant & Benton, 2000). Additional details to calculate the LTRE can be found in Caswell (2010) and Ellner, Childs & Rees (2016).

A11 RESULTS INDIVIDUAL BASED MODEL

The individual-based model (IBM) was used to estimate the uncertainty caused by missing data in the form of a posterior predictive check (Gabry *et al.*, 2019; Gelman *et al.*, 2013). We expected that the IBM would simulate very different populations over the 1 000 simulations and/or very different from the observed data if the parameter estimates calculated with the statistical analyses were biased. The predicted number of dominants, helpers and non-helpers was similar between the 1 000 simulations (Fig. A22). There was also a good overlap between observations and predictions from the IBM (Fig. A23). Therefore, we could conclude that potential biases due to missing data were negligible.

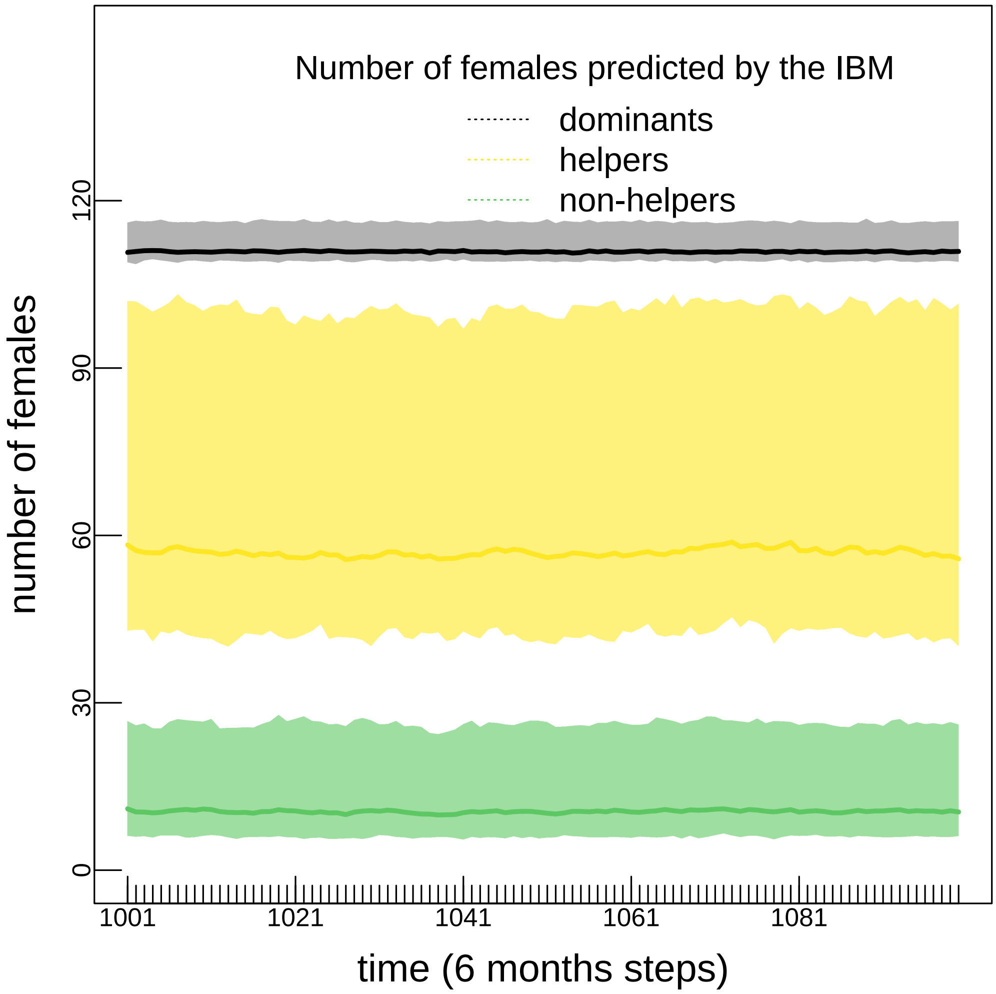

Figure A22. Predicted number of dominants (black), helpers (yellow), and non-helpers (green) in the 1 000 populations simulated from the individual-based model (IBM). Lines represent the mean, while shaded areas represent the interquartile range.

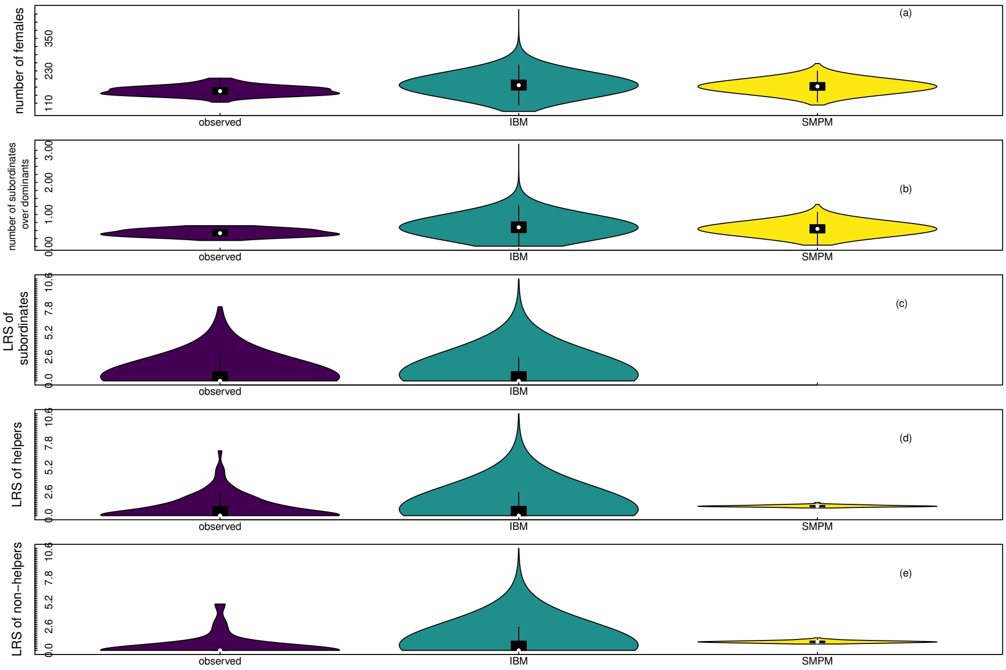

Figure A23. Comparison between observed population parameters (purple) for female Seychelles warblers on Cousin island, with those predicted by 1 000 simulations of the individual based model (IBM, blue-green) and the stochastic matrix population model (SMPM, yellow). The lifetime reproductive success (LRS) of subordinates is the LRS of all subordinates, including helpers, non-helpers and subordinates without behavioural data to classify them as either helpers or non-helpers. The observed numbers of females and subordinates were calculated over 40 field seasons; while the sample sizes available to calculate the observed LRS were based on 570 subordinates, 114 helpers and 28 non-helpers. Data are plotted as a boxplot (with median and interquartile range) combined with a kernel density plot (R package vioplot 0.5).

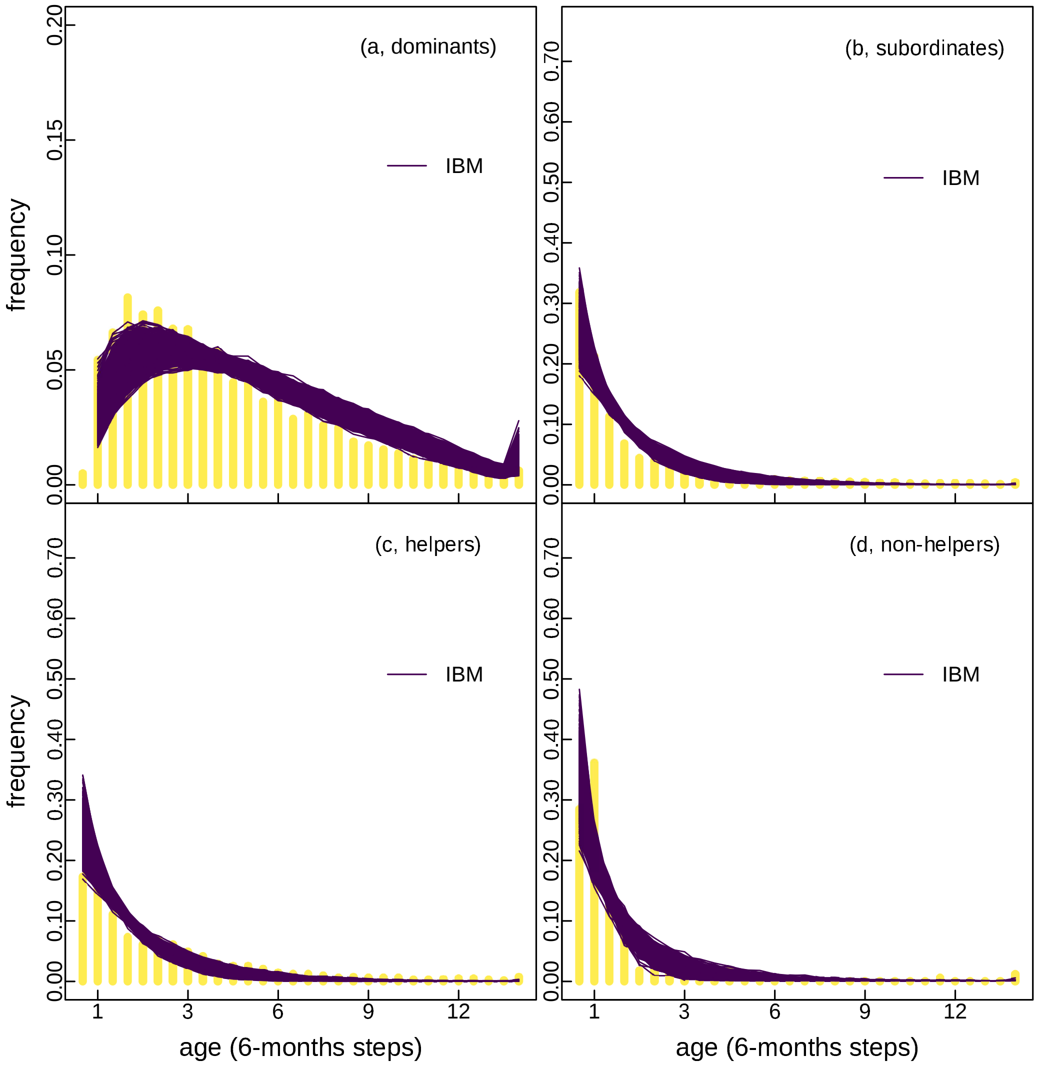

Figure A24. Mean age-distributions (1 000 purple lines) for dominants (a), subordinates (b), helpers (c) and non-helpers (d) derived from the 1 000 simulations of the individual based model (IBM). Yellow vertical bars indicate the observed distribution of age over the entire study. Subordinates (b) include different groups in the IBM *versus* the observed data. In the IBM they represent helpers and non-helpers, while in the observations they include helpers, non-helpers and those subordinates that could not be classified as either helpers or non-helpers because of a lack of behavioural observations. Therefore, the observed age distribution of the helpers and the non-helpers combined differs from the age-distribution of all the observed subordinates (Fig. A19). Sample size differs in the four graphs ($n = 5\,200$ for dominant, $n = 2\,341$ for subordinates, $n = 822$ for helpers, $n = 169$ for non-helpers). The range of the $y-$axis differs in plot (a) (0.00-0.20) *versus* the other plots (0.00-0.50).

A12 ADDITIONAL RESULTS FOR FITNESS COMPONENTS

The inclusion of indirect fitness benefits into the SMPM would have allowed to calculate more precise life-history differences between helpers and non-helpers. We expect that the inclusion of indirect fitness benefits could increase the difference in fitness of helpers and non-helpers. However we expect this difference to be marginally important because direct fitness benefits are about six times larger than indirect fitness benefits in Seychelles warblers (Richardson, Burke & Komdeur, 2002).

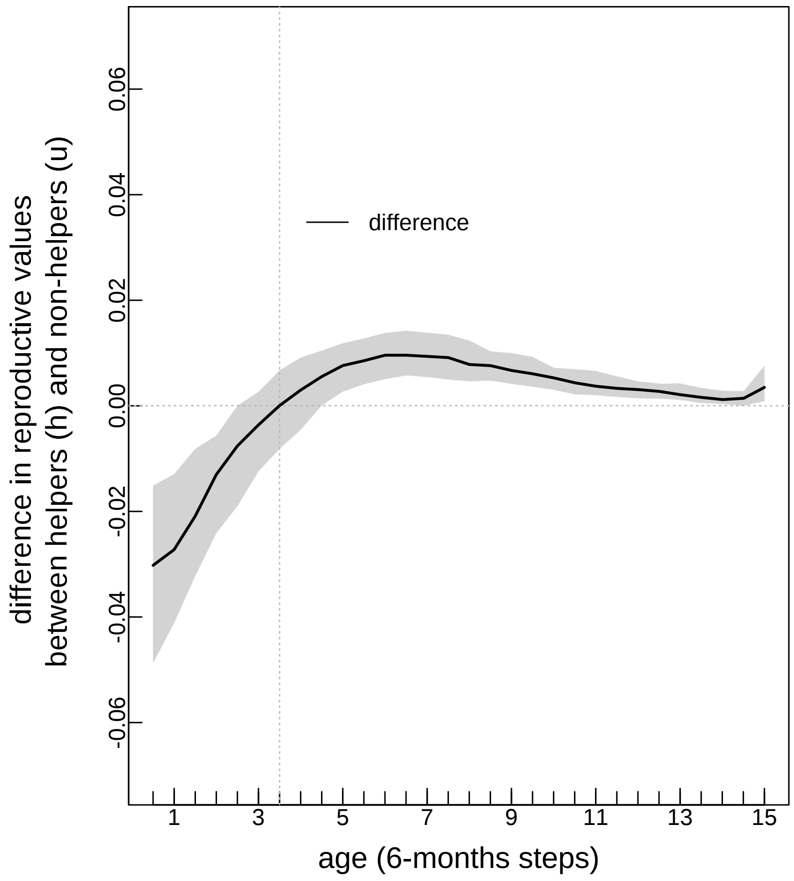

Figure A25. Difference in the reproductive values of helpers and non-helpers (black line). The shaded area represents the interquartile range. The dotted grey line represents the zero line, which corresponds to no difference in the reproductive values of helpers and non-helpers. The reproductive value of helper is lower than the reproductive values of non-helpers up to age 4. Afterwards, the reproductive values are higher for helpers than non-helpers. The reproductive values are a measure of the relative contribution to future generations and across environments.

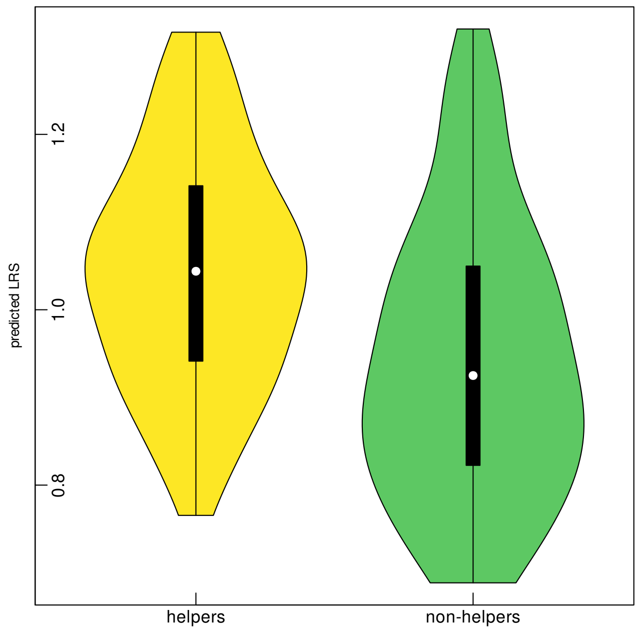

Figure A26. Comparison between predicted lifetime reproductive success (LRS) of helpers (yellow) and non-helpers (green) calculated by the stochastic matrix population model (SMPM). The median value is higher for helpers than non-helpers, but the interquartile ranges (range between the first and third quartile) overlap. Data are plotted as a boxplot (with median and interquartile range) combined with a kernel density plot (R package vioplot 0.5).

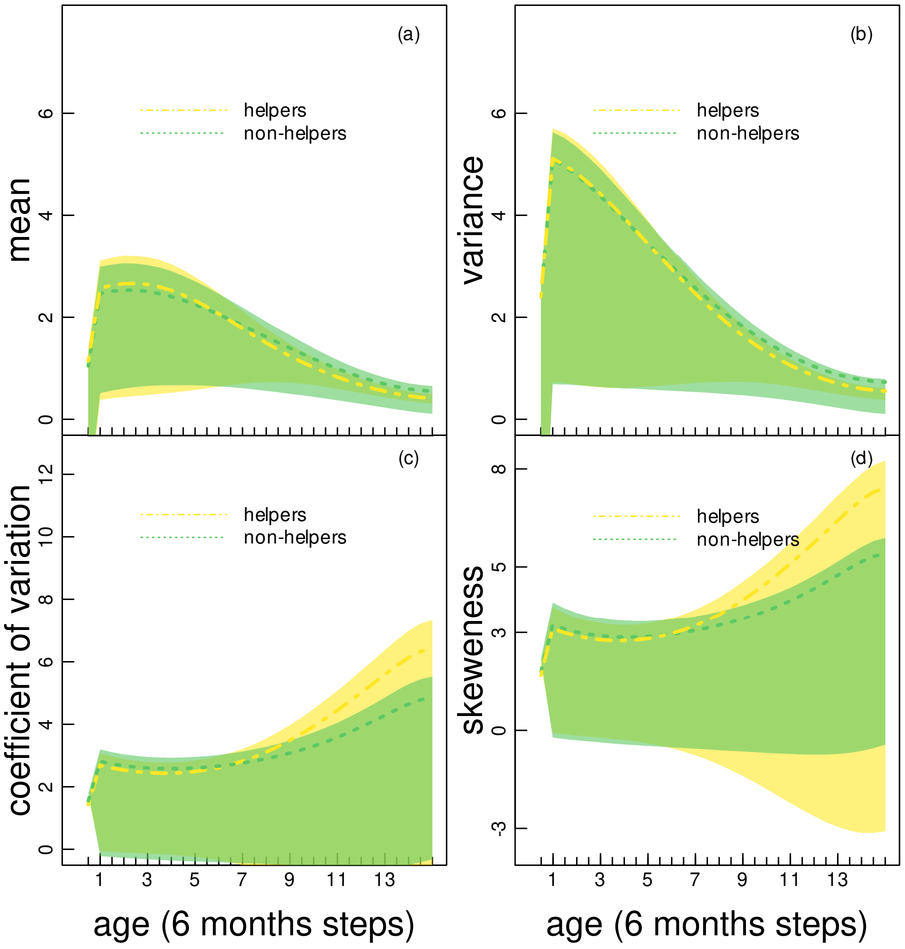

Figure A27. Mean (a), variance (b), coefficient of variation (c) and skewness (d) of remaining lifetime reproductive success of helpers (yellow lines) and non-helpers (green lines) as a function of age. Values were calculated from the equations presented in Caswell (2011); van Daalen & Caswell (2017) to account for the stochasticity in demographic events during an individual's lifetime. The shaded area represents the interquartile ranges around the lines. Mean (a) and variance (b) in reproduction decline with age and are very similar for helpers and non-helpers. The coefficient of variation (c) expresses the relative variability of reproductive output with age, and it increases with age for both helpers and non-helpers. The skewness (d) also increases with age for both helper and non-helpers.

BIBLIOGRAPHY

Brouwer, L., Richardson, D.S., Eikenaar, C. & Komdeur, J. (2006) The role of group size and environmental factors on survival in a cooperatively breeding tropical passerine. *Journal of Animal Ecology*, **75**, 1321–1329.

Caswell, H. (2001) *Matrix population models*. Wiley Online Library.

Caswell, H. (2010) Life table response experiment analysis of the stochastic growth rate. *Journal of Ecology*, **98**, 324–333.

Caswell, H. (2011) Beyond R0: demographic models for variability of lifetime reproductive output. *PloS one*, **6**, e20809.

Crick, H.Q. (1992) Load-lightening in cooperatively breeding birds and the cost of reproduction. *Ibis*, **134**, 56–61.

van Daalen, S.F. & Caswell, H. (2017) Lifetime reproductive output: individual stochasticity, variance, and sensitivity analysis. *Theoretical Ecology*, **10**, 355–374.

Edwards, H.A., Burke, T. & Dugdale, H.L. (2017) Repeatable and heritable behavioural variation in a wild cooperative breeder. *Behavioral ecology*, **28**, 668–676.

Ellner, S.P., Childs, D.Z. & Rees, M. (2016) *Data-driven modelling of structured populations*. Springer.

Flegal, J.M., Hughes, J., Vats, D. & Dai, N. (2020) *mcmcse: Monte Carlo Standard Errors for MCMC*. Riverside, CA, Denver, CO, Coventry, UK, and Minneapolis, MN. R package version 1.4-1.

Gabry, J., Simpson, D., Vehtari, A., Betancourt, M. & Gelman, A. (2019) Visualization in Bayesian workflow. *Journal of the Royal Statistical Society: Series A (Statistics in Society)*, **182**, 389–402.

Gelman, A., Carlin, J.B., Stern, H.S., Dunson, D.B., Vehtari, A. & Rubin, D.B. (2013) *Bayesian data analysis*. CRC press.

Grant, A. & Benton, T.G. (2000) Elasticity analysis for density-dependent populations in stochastic environments. *Ecology*, **81**, 680–693.

Groenewoud, F., Kingma, S.A., Hammers, M., Dugdale, H.L., Burke, T., Richardson, D.S. & Komdeur, J. (2018) Subordinate females in the cooperatively breeding Seychelles warbler obtain direct benefits by joining unrelated groups. *Journal of Animal Ecology*, **87**, 1251–1263.

Hadfield, J., Richardson, D. & Burke, T. (2006) Towards unbiased parentage assignment: combining genetic, behavioural and spatial data in a Bayesian framework. *Molecular Ecology*, **15**, 3715–3730.

Hammers, M., Richardson, D.S., Burke, T. & Komdeur, J. (2013) The impact of reproductive investment and early-life environmental conditions on senescence: Support for the disposable soma hypothesis. *Journal of Evolutionary Biology*, **26**, 1999–2007.

Hammers, M., Kingma, S.A., van Boheemen, L.A., Sparks, A., Burke, T., Dugdale, H., Richardson, D.S. & Komdeur, J. (2019a) Helpers compensate for age-related declines in parental care and offspring survival in a cooperatively breeding bird.

Hammers, M., Kingma, S.A., Spurgin, L.G., Bebbington, K., Dugdale, H.L., Burke, T., Komdeur, J. & Richardson, D.S. (2019b) Breeders that receive help age more slowly in a cooperatively breeding bird. *Nature communications*, **10**, 1–10.

Hammers, M., Richardson, D.S., Burke, T. & Komdeur, J. (2012) Age-dependent terminal declines in reproductive output in a wild bird. *PLoS One*, **7**, e40413.

Kingma, S.A. (2017) Direct benefits explain interspecific variation in helping behaviour among cooperatively breeding birds. *Nature communications*, **8**, 1–7.

Komdeur, J., Burke, T., Dugdale, H. & Richardson, D.S. (2016) *Seychelles warblers: complexities of the helping paradox*, pp. 197–216. Cambridge Univ. Press, Cambridge, UK.

Komdeur, J. & Pels, M.D. (2005) Rescue of the Seychelles warbler on Cousin Island, Seychelles: The role of habitat restoration. *Biological Conservation*, **124**, 15–26.

Lemoine, N.P. (2019) Moving beyond noninformative priors: why and how to choose weakly informative priors in Bayesian analyses. *Oikos*, **128**, 912–928.

McElreath, R. (2020) *Statistical rethinking: A Bayesian course with examples in R and Stan.* CRC press.

R Core Team (2020) *R: A Language and Environment for Statistical Computing*. R Foundation for Statistical Computing, Vienna, Austria.

Richardson, D.S., Jury, F.L., Blaakmeer, K., Komdeur, J. & Burke, T. (2001) Parentage assignment and extra-group paternity in a cooperative breeder: The Seychelles warbler (*Acrocephalus sechellensis*). *Molecular Ecology*, **10**, 2263–2273.

Richardson, D.S., Burke, T. & Komdeur, J. (2002) Direct benefits and the Evolution of Female-biased Cooperative Breeding in Seychelles Warblers. *Evolution*, **56**, 2313–2321.

Wright, D.J., Spurgin, L.G., Collar, N.J., Komdeur, J., Burke, T. & Richardson, D.S. (2014) The impact of translocations on neutral and functional genetic diversity within and among populations of the Seychelles warbler. *Molecular ecology*, **23**, 2165–2177.
